# Synthetic Efforts to Investigate the Effect of Planarizing the Triarylamine Geometry in Dyes for Dye-Sensitized Solar Cells

**DOI:** 10.1021/acsomega.2c03163

**Published:** 2022-06-16

**Authors:** David Moe Almenningen, Veslemøy Minge Engh, Eivind Andreas Strømsodd, Henrik Erring Hansen, Audun Formo Buene, Bård Helge Hoff, Odd Reidar Gautun

**Affiliations:** † Department of Chemistry, Norwegian University of Science and Technology, Høgskoleringen 5, 7491 Trondheim, Norway; ‡ Department of Materials Science and Engineering, Norwegian University of Science and Technology, Sem Sælands vei 12, 7491 Trondheim, Norway; § Department of Civil and Environmental Engineering, Norwegian University of Science and Technology, Høgskoleringen 7a, 7034 Trondheim, Norway

## Abstract

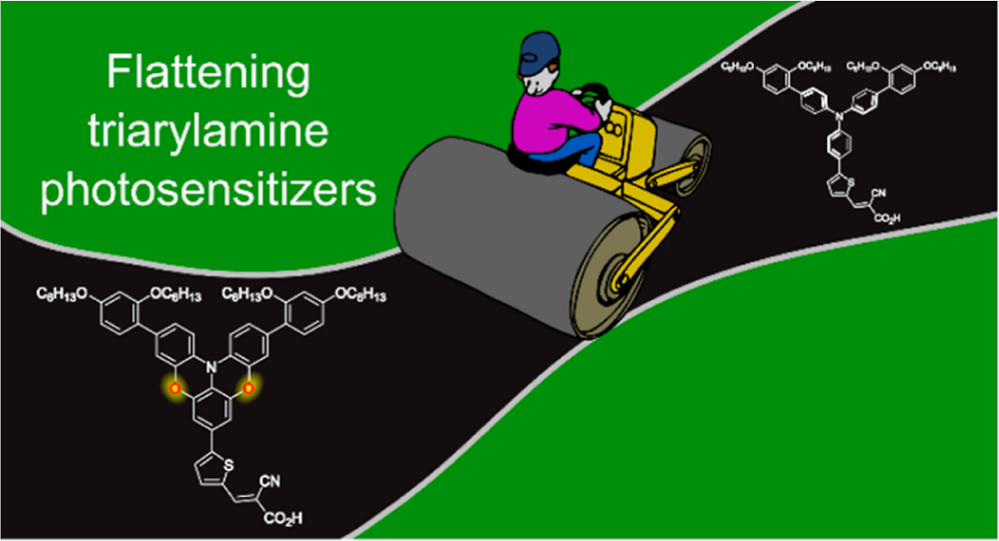

The geometry of a dye for dye-sensitized solar cells (DSSCs) has a major impact on its optical and electronic properties. The dye structure also dictates the packing properties and how well the dye insulates the metal–oxide surface from oxidants in the electrolyte. The aim of this work is to investigate the effect of planarizing the geometry of the common triarylamine donor, frequently used in dyes for DSSC. Five novel dyes were designed and prepared; two employ conventional triarylamine donors with thiophene and furan π-spacers, two dyes have had their donors planarized through one sulfur bridge (making two distinct phenothiazine motifs), and the final dye has been planarized by forming a double phenoxazine. The synthesis of these model dyes proved to be quite challenging, and each required specially designed total syntheses. We demonstrate that the planarization of the triarylamine donor can have different effects. When planarization was achieved by a 3,7-phenothiazine and double phenoxazine structures, improved absorption properties were noted, and a panchromatic absorption was achieved by the latter. However, an incorrect linking of donor and acceptor moieties has the opposite effect. Further, electrochemical impedance spectroscopy revealed clear differences in charge recombination depending on the structure of the dye. A drawback of planarized dyes in relation to DSSC is their low oxidation potentials. The best photovoltaic performance was achieved by 3,7-phenothazine with furan as a π-spacer, which produces a power conversion efficiency of 5.2% (*J*_sc_ = 8.8 mA cm^–2^, *V*_oc_ = 838 mV, FF = 0.70).

## Introduction

Ever since its original report in 1991,^1^ the dye-sensitized solar cell (DSSC) has been a keen object to study for many researchers worldwide. The DSSC enjoys a relatively simple structure with three key components, the mesoporous semiconducting metal oxide (most commonly TiO_2_), the dye which is adsorbed on the metal oxide, and the redox shuttle responsible for regenerating the dye with electrons from the counter electrode.^2^ After more than 30 years of constant research trying to improve the power conversion efficiency (PCE) of the DSSC, a laboratory-scale cell with a PCE of 14.3% has been reported.^3^ This is obviously a lot lower than that of the conventional silicon-based solar cells, but recent developments in redox shuttles and dye design have made the DSSC the most efficient technology for ambient light photovoltaics.^4,5^ Excellent indoor lighting PCE achieved by DSSCs lends itself nicely to be used to power the ever-increasing internet-of-things applications.^6−8^ The aesthetically pleasing aspect of the DSSC is another inherent strength of this technology, which could be exploited by the use of DSSC in building-integrated photovoltaics (BIPVs).^9,10^ The recent development of near-infrared absorbing dyes^11^ and photochromic dyes^12^ will even allow for transparent devices that could be used in BIPV as power-generating windows.^13^

A marked improvement in DSSC performance was seen when one-electron metal complex redox shuttles were employed in place of the I^–^/I_3_^–^ redox shuttle.^14^ These metal complexes, mainly based on Co and Cu, display redox potentials that are more closely matched to the oxidation potential of the dyes in the cell. Although this feature effectively reduced the overpotential losses in the DSSC, there were some initial problems with the fact that these complexes are easily reduced by electrons in TiO_2_.^15^ To combat this phenomenon, it became important to find a way to passivate the surface of TiO_2_, for instance, through the use of alkoxysilanes as a coating material for the mesoporous electrode.^16^ A more efficient strategy was the use of bulky organic dyes, such as the tetra-alkoxy-substituted triarylamine motif, commonly referred to as the Hagfeldt donor, that has been used in several highly successful dyes.^3,5,17−20^ The triarylamine scaffold holds a three-dimensional propeller shape that provides an umbrella effect for the surface of TiO_2_, where it prevents the electrolyte from approaching the semiconductor and recombining with the electrons there.^21^

A possible downside of this out-of-plane geometry is that the aromatic system suffers from a sub-optimal overlap between the molecular orbitals. This is detrimental to the absorption properties of the dyes and in turn the possible photocurrent that it is possible to extract from them. As a result of this, triarylamine dyes are frequently extended with large π-spacers to improve their absorption properties^19,22,23^ at the expense of complicating synthesis and increasing the cost of production. Planarization of the donor has previously been a successful design concept to improve absorption properties, most notably perhaps is the wide absorption spectra of ullazine-based dyes without any π-spacers.^24^ Planarization of the donor has also shown to boost the intramolecular charge transfer (ICT) of dyes^25^ and improve the interfacial charge-transfer processes of indoline and carbazole dyes.^26,27^

**Figure 1:**
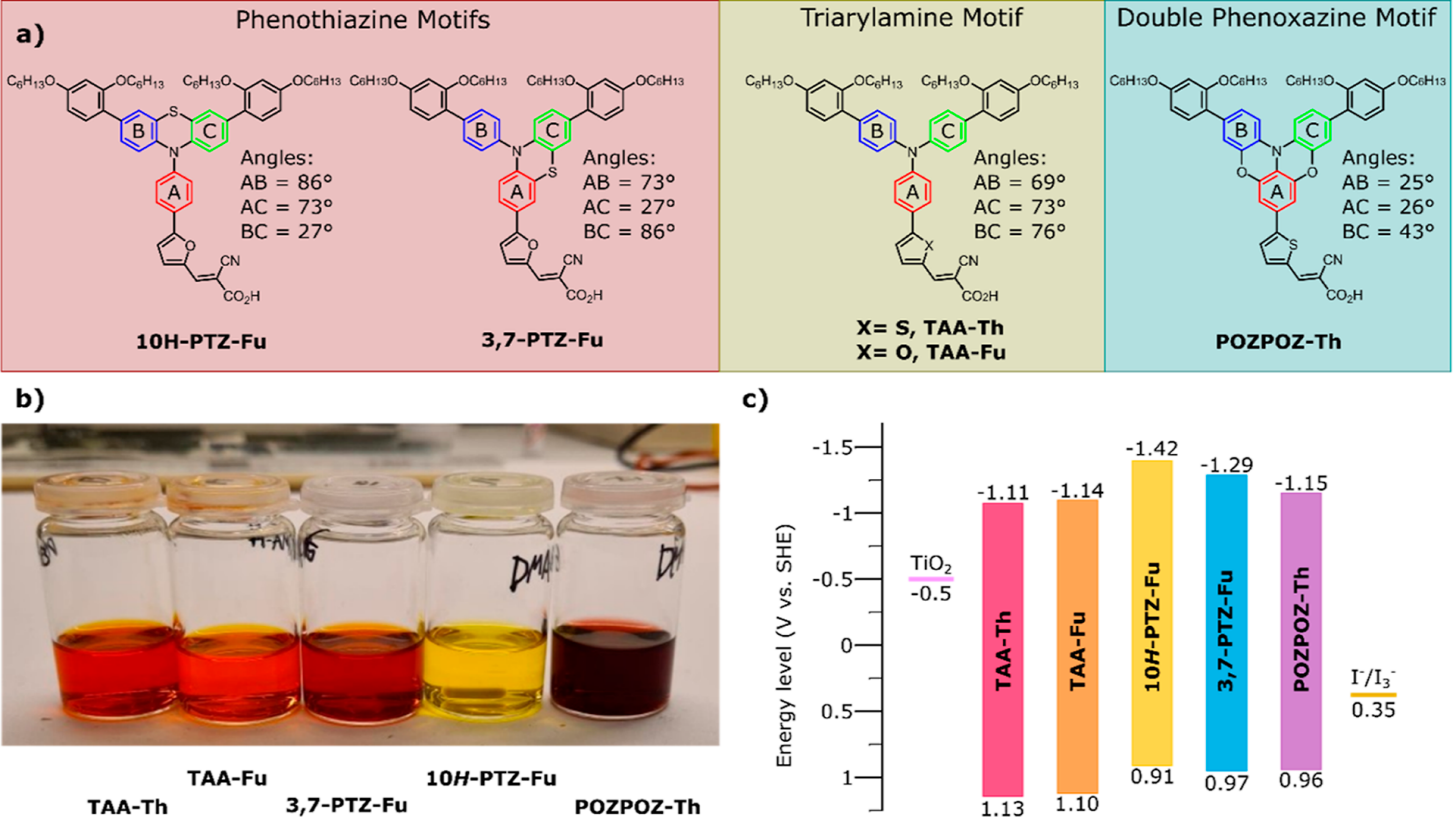
(a) Molecular structures and design concepts of the five different dyes reported in this paper. The degree of planarization is postulated from the out-of-plane angle between the three central phenyl rings based on crystallographic data of 2-chloro-10-phenylphenothiazine,^31^ triphenylamine,^32^ and benzo[5,6][1,4]oxazino[2,3,4-*kl*]phenoxazine.^33^ (b) Staining solutions of the dyes; the concentration of each dye is 0.5 mM in a mixture of acetonitrile and THF (43:57, v/v). (c) Energy levels of the frontier orbitals of the dyes, TiO_2_, and the I^–^/I_3_^–^ redox shuttle.

There are studies on planarizing the triarylamine donor through bridging with methylene^28^ and diarylmethylene^29^ compared to the “free” triarylamine. Planarizing the triarylamine donor through two sulfur bridges reported worse photovoltaic performance compared to a “free” triarylamine reference dye.^30^ To study the effect of planarizing the triarylamine donor with heteroatom bridges, we report herein five novel dyes with moderately sized π-spacers based on the common triarylamine donor motif, see Figure 1a. The degree of planarization on these donors varies from the “free” triarylamine motif to the single planarization of the phenothiazine motifs and all the way to the double phenoxazine motif. We demonstrate the possibility of tuning absorption through planarizing of the triarylamine donor, and the impact of these alterations on the color of the dyes is shown in Figure 1b. By comparing the photovoltaic performance of the “free” triarylamine dyes to the planarized ones, we aim to investigate the importance of the three-dimensional structure on solar cell performance.

## Experimental Section

### Materials

The reference dye **N719** was purchased from Dyenamo AB (Sweden), 2-bromo-5-iodophenol (**25**) was purchased from Apollo Scientific (UK), and 5-bromo-1,3-difluoro-2-nitrobenzene was purchased from abcr (Germany). The remaining chemicals and solvents used were all purchased from Merck. A full account of the synthetic procedures is given in the ESI.

### Electrochemical Characterization

Cyclic voltammetry (CV) experiments were carried out using a Versastat 3 Potentiostat; the data were acquired using the Versastudio software. A stained TiO_2_ photoanode as the working electrode, a graphite carbon counter electrode, and a Ag/AgCl reference were the components of the three-electrode system. The scan speed was 10 mV s^–1^, and the supporting electrolyte was 0.1 M LiTFSI in dry acetonitrile.

### Fabrication of DSSCs

The anodes were prepared from FTO glass (NSG10, Nippon Sheet Glass), which was cleaned in a KOH solution (150 g/L) in 70 w % ethanol under sonication for 45 min. Immersion of the glass in aqueous TiCl_4_ solution (40 mM) at 70 °C for 2 × 45 min, followed by rinsing with deionized water and ethanol, was carried out before sintering TiO_2_ for 1 h at 500 °C on a hotplate to deposit a blocking layer on the FTO sample. Pastes of TiO_2_ were screen-printed onto FTO (53 T mesh, area 0.238 cm^2^, Seritec Services S.A.); the first two active layers (18NR-T, Dyesol) were printed with 10 min heating on a hotplate at 125 °C after each layer. A scattering layer (WER2-O, Dyesol) was ultimately printed, and TiO_2_ was sintered using a programmable furnace at set temperatures of 125, 250, 375, 450, and 500 °C for 5, 5, 5, 15, and 15 min with a ramping time of 10 min. Before staining, the electrodes were annealed at 500 °C for 30 min using a hotplate. The thicknesses of the TiO_2_ layers were measured using a Veeco Dektak 150 profilometer and found to be 2 × 6 μm 18NR-T + 6 μm WER2-O.

The counter electrodes were prepared from TEC10 FTO glass supplied by Sigma-Aldrich. Holes were drilled into the electrodes from the FTO side using a diamond drill bit; this procedure was carried out under water. The glass plates were then cleaned using Deconex 21 (aq, 2 g/L), deionized water, ethanol, and acetone in an ultrasonic bath for 15 min for each. A solution of H_2_PtCl_6_ (10 mM) in 2-propanol was dropcast on FTO before heating at 400 °C for 15 min with a hot air gun, forming the catalytic layer of Pt.

The photoanodes were placed in the dye bath while still holding ∼80 °C from the annealing procedure and stored in a chamber at 30 °C overnight. The dye baths were prepared using a mixture of acetonitrile and tetrahydrofuran (THF) (43:57, v/v) to make a solution of the dye (0.5 mM) and co-adsorbent CDCA (5 mM). The staining of the reference **N719** was done similarly, but the solvent mixture used was in this case *t*-butanol and acetonitrile (1:1, v/v). Following 15 h of staining, the electrodes were rinsed in acetonitrile for 2 min and then sealed to the counter electrode using Surlyn (25 μm, Solaronix) in a drybox. A 4 × 20 s treatment of the cell using a 50 W PTC heat element was sufficient to seal the cells. The electrolyte was vacuum-backfilled into the device; the filling hole was sealed with Surlyn and a glass cover disk. To complete the devices, the electrodes were painted with silver conducting paint (Electrolube, SCP). The electrolyte employed was a previously reported electrolyte A6141, consisting of butylmethylimidazolium iodide (0.60 M), I_2_ (0.03 M), guanidinium thiocyanate (0.10 M), and 4-*t*-butylpyridine (0.60 M) dissolved in a mixture of acetonitrile and valeronitrile (85:15, v/v).^34^

### Device Characterization

*J*–*V* curves were obtained under 1 sun illumination AM 1.5G illumination provided by a Sciencetech SP300B solar simulator, calibrated with a Newport Reference Cell (91150V), connected to a Keithley 2450 SourceMeter. A mask with an active area of 0.159 cm^2^ was used on all the *J*–*V* measurements. IPCE measurements were carried out using a halogen lamp (Ocean Optics HL-2000) and a monochromator (Spectral Products CM110) connected to a Keithley 2450. The devices and the reference photodiode (Thorlabs, FDS100-CAL) were covered with a mask with a size of 0.049 cm^2^. The electrochemical impedance properties were measured under constant illumination at 479 nm (12.6 mW cm^–2^) and following the procedure we reported in our previous publication.^35^

## Results and Discussion

### Dye Design

**TAA-Th** (thieno-linker) and **TAA-Fu** (furo-linker) are novel dyes, closely related to the dye **D35** described by Hagberg et al., reaching a PCE of 6% in the initial study.^36^ The dye **D35** was one of the first dyes to demonstrate the usefulness of the alkoxy-substituted triarylamine donor to provide surface protection for the novel one-electron Co^2+/3+^ redox shuttle.^37^ It has since proven to be compatible with Cu electrolytes allowing for even higher voltages in DSSC.^38^ It is expected that some increase in recombination resistance could be achieved by changing to hexyl side chains as presented in **TAA-Th** and **TAA-Fu**. In our previous study on phenothiazine, we found that dyes with a furan π-spacer perform slightly better than the corresponding thiophene analogue.^39^ By comparing these two new triarylamine dyes, we will determine whether this holds for this dye class as well.

The dyes **10*H*-PTZ-Fu** and **3,7-PTZ-Fu** are designed in such a way that rotation and twisting of the aryl amine units are partially hindered by a covalent carbon–sulfur bond, placed symmetrically and asymmetrically respectively. The geometry of **10*H*-PTZ-Fu** is atypical for phenothiazine dyes as our recent review of the entire class of phenothiazine dyes found this motif in only 4% of all dyes reported.^40^ The molecular geometry of **3,7-PTZ-Fu** follows the most conventional of phenothiazine dye designs, where dyes decorated with the auxiliary donor and π-spacer in positions 3 and 7 on phenothiazine make up 24% of all dyes.^40^ The fully planarized double phenoxazine donor, seen for **POZPOZ-Th**, has not previously been reported. However, some double phenothiazine helicene analogues have been used in dyes for DSSC by Kim et al.^30^

To assess the planarity of the different donors reported herein, we use previously reported crystallographic data of the simpler compounds triphenylamine,^32^ 2-chloro-10-phenylphenothiazine,^31^ and benzo[5,6][1,4]oxazino[2,3,4-*kl*]phenoxazine.^33^ The three central rings of the “free” triarylamine donor (**TAA-Th** and **TAA-Fu**) are all expected to be 69–76° out of plane with each other. The sulfur-bridged compounds (**10*H*-PTZ-Fu** and **3,7-PTZ-Fu**) have the angle between two of their three central rings reduced to 27°. The double phenoxazine motif (**POZPOZ-Th**) has through the two oxygen bridges reduced the angle between the three central rings to 25 and 26°.

### Synthesis

**Scheme 1:**
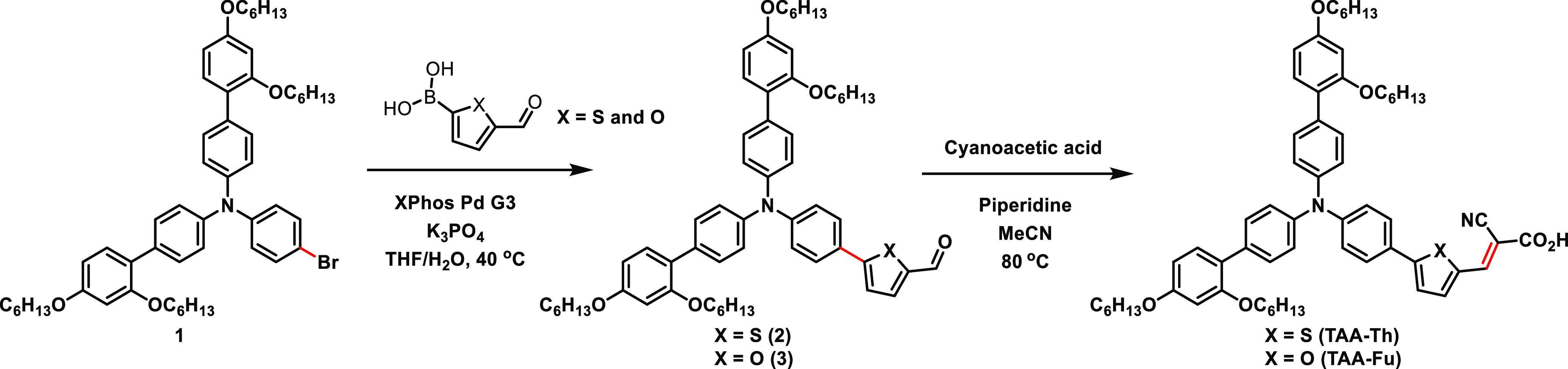
Synthesis of **TAA-Th** and **TAA-Fu**

The synthesis route of **TAA-Th** and **TAA-Fu** is shown in Scheme 1. The advanced triarylamine fragment **1** was prepared as previously described by our group.^41^ Then, a Suzuki cross-coupling with (5-formylthiophen-2-yl)boronic acid and (5-formylfuran-2-yl)boronic acid was carried out. These boronic acids are rather unstable,^42^ so we selected the very active XPhos palladium third-generation precatalyst for the transformation.^43^ Fortunately, when performed at 40 °C, the reaction gave the thiophene-containing aldehyde **2** in 40% yield and furan analogue **3** in 56% yield. Finally, a Knoevenagel condensation installed the cyanoacrylic acid anchoring group, giving **TAA-Th** and **TAA-Fu** in yields of 95 and 75%, respectively.

**Scheme 2:**
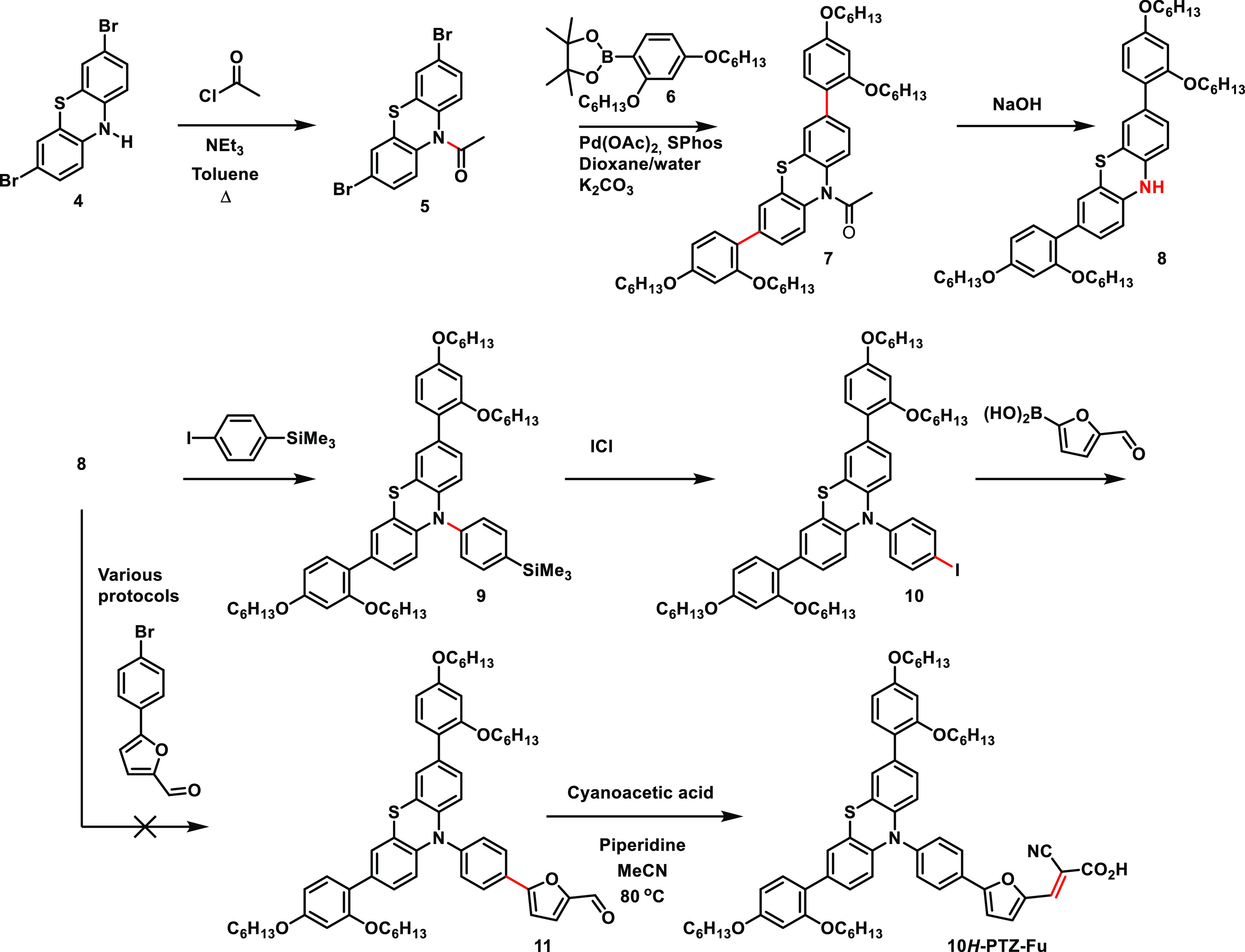
Synthesis of **10*H*-PTZ-Fu**

Synthesis of **10*H*-PTZ-Fu** was more cumbersome. We planned to employ 3,7-diarylated phenothiazine **8** in a Buchwald–Hartwig amination, see Scheme 2. To reach this critical intermediate, a direct double Suzuki coupling on 3,7-dibromo-10*H*-phenothiazine (**4**) was attempted, but purification of the material proved very difficult. To ensure sufficient amounts of the target dye to work with, we set out to improve the first step of the synthesis route. This was done by protecting the *N*-10 position of **4** as *N*-acetyl, allowing for a decent Suzuki cross-coupling between phenothiazine **5** and pinacol boronate ester **6**,^41^ giving **7** in 65% yield. A facile deprotection then gave the key intermediate **8** (Scheme 2).

The Buchwald–Hartwig amination of **8** was first evaluated in a model reaction using 4-bromoanisole, which worked satisfactorily. However, the corresponding reaction with 5-(4-bromophenyl)furan-2-carbaldehyde, intended to give compound **11** directly, failed to convert the starting material under a number of reaction conditions.^44−48^ Instead of tuning this reaction, we went for the longer route via silylated **9**. The Buchwald–Hartwig amination utilizing 1-bromo-4-(trimethylsilyl)benzene proceeded well, giving **9** in 91% yield. Conversion of trimetylsilyl groups to the corresponding iodide has previously been performed on simpler phenothiazines in high yields.^49,50^ However, compound **9** is fairly electron-rich and we experienced the formation of byproducts, causing a difficult purification. This limited the isolated yield to 19%. The identities of the byproducts were indicated by mass spectroscopy to be the protodesilylated derivative, a di-iodinated product, and a tri-iodinated derivative. The structure of the latter compound was confirmed by NMR spectroscopic studies of a purified sample; the identity and the corresponding NMR spectra are shown in the Supporting Information. The iodinated derivative **10** was then subjected to Suzuki cross-coupling with 2-formylfuran-5-boronic acid. When employing the PdCl2(dppf) catalyst at 80 °C, no consumption of the starting material was observed. However, upon changing the catalyst to XPhos Pd G3 at 40 °C and using 1.6 equivalents of boronic acid allowed for isolation of the furan-functionalized **11** in 43% yield after purification. A Knoevenagel condensation concluded the synthesis and gave **10*H*-PTZ-Fu** in a yield of 85%.

**Scheme 3:**
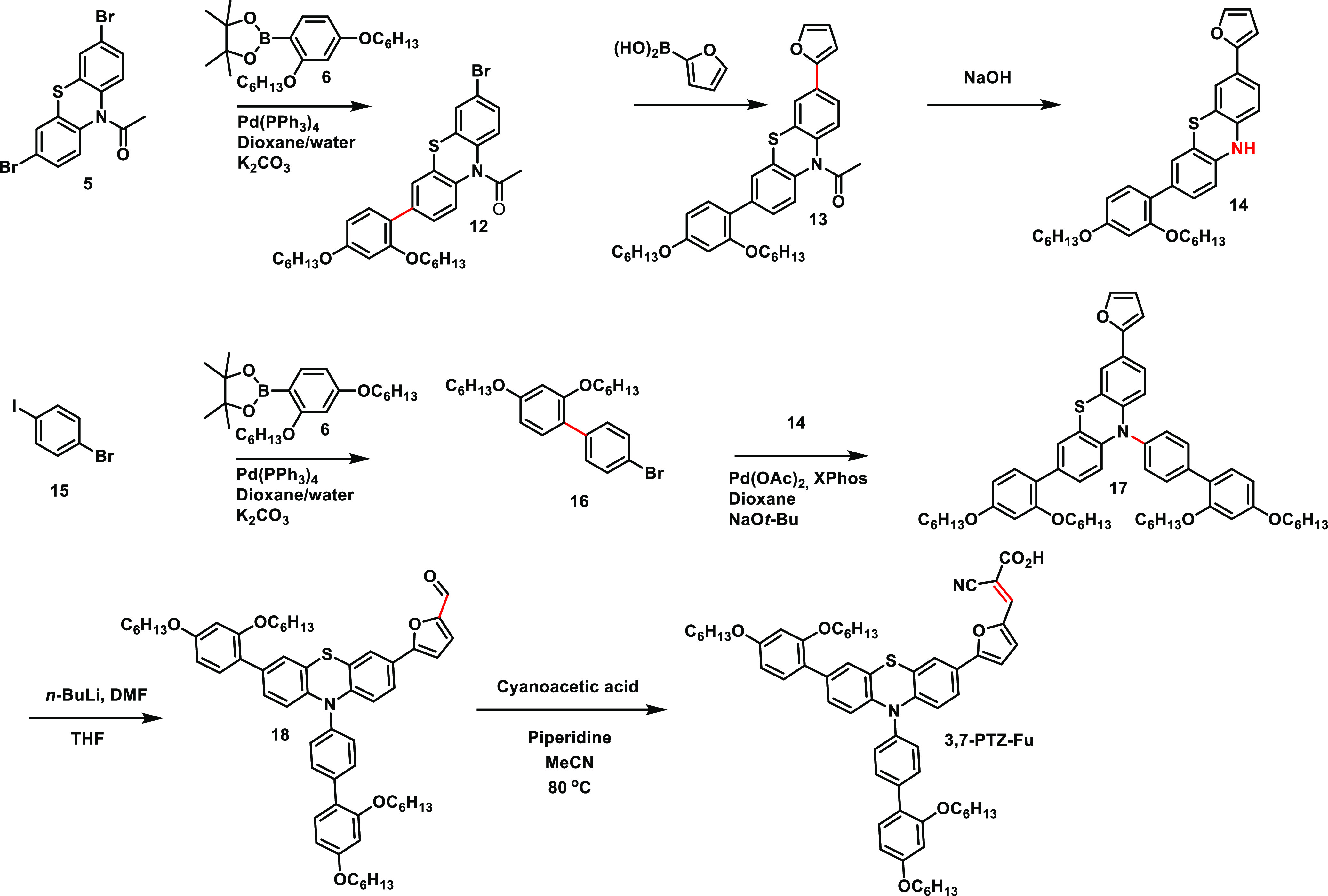
Synthesis of **3,7-PTZ-Fu**

The synthesis of **3,7-PTZ-Fu** borrowed several of the successful concepts from the synthesis route of **10*H*-PTZ-Fu**; however, special precautions were taken to ensure the asymmetric substitution on the phenothiazine donor. Starting with the *N*-acetyl-protected building block **5** (Scheme 3), we coupled it with 1.2 equivalents of pinacol boronate ester **6** in a Suzuki–Miyaura reaction. We have demonstrated on another set of phenothiazine sensitizers that these reaction conditions give an approximate 1:2:1 distribution of the starting material, monocoupled product, and dicoupled product.^39^ Following purification by column chromatography, intermediate **12** was isolated in a yield of 45%. The furanyl π-spacer was introduced through a subsequent Suzuki reaction catalyzed by PdCl_2_(dppf), and compound **13** was obtained in a yield of 64%. Compound **14** was obtained from a simple hydrolysis of the protection group, and the coupling partner, **16**, was prepared from a Suzuki coupling on the iodide of 1-bromo-4-iodobenzene with pinacol boronate ester **6**. A Buchwald–Hartwig reaction between **14** and **16** produced the donor part of the dye, and intermediate **17** was obtained in a yield of 65%. To introduce the aldehyde functionality, the furan moiety was lithiated using *n*-BuLi and then quenched with DMF to produce the advanced intermediate **18** in a yield of 34%. A Knoevenagel reaction was again used to complete the finished dye, giving **3,7-PTZ-Fu** in a yield of 65%.

**Scheme 4:**
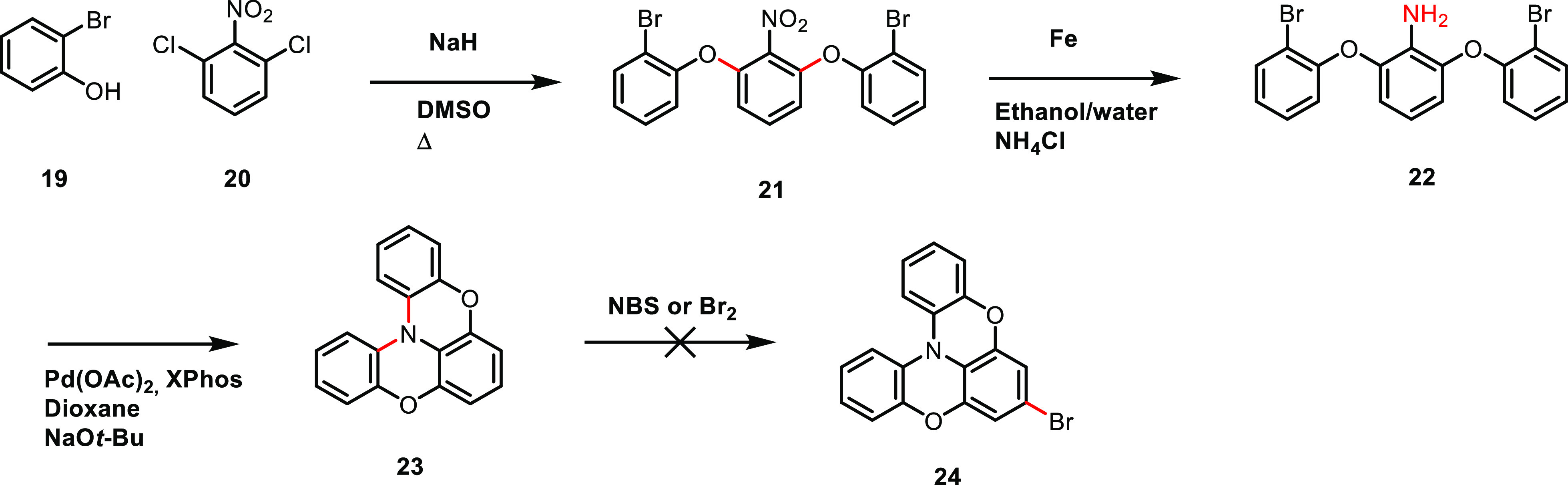
Our Original Synthesis of Key Intermediate **24**

The synthesis of **POZPOZ-Th** proved to be the most laborious of the dyes in this series as our original strategy, shown in Scheme 4, was unsuccessful. This synthesis is based on the route reported by Kuratsu et al.^33^ for the synthesis of **23**. The first step is a nucleophilic aromatic substitution using the dimsyl anion as a base, which afforded **21** in a yield of 56%. We chose a Fe/NH_4_Cl reduction procedure instead of using the hydrazine and Pd/C conditions reported in the original synthesis to avoid reducing the aromatic bromide. This approach worked as intended with no signs of a competing reduction of the bromides. Aniline **22** was used in the next step without the need for any purification beyond removing the inorganic solids. The final step in preparing helicene **23** involved a double intramolecular Buchwald–Hartwig amination catalyzed by Pd(OAc)_2_ and XPhos, and **23** was isolated in a yield of 50% over two steps from **21**. Unfortunately, we were unsuccessful in selectively brominating **23**, even though two patents report the preparation of **24** by bromination.^51,52^ This led us to devise a synthesis strategy which would not rely on a selective electrophilic aromatic substitution (EAS).

**Scheme 5:**
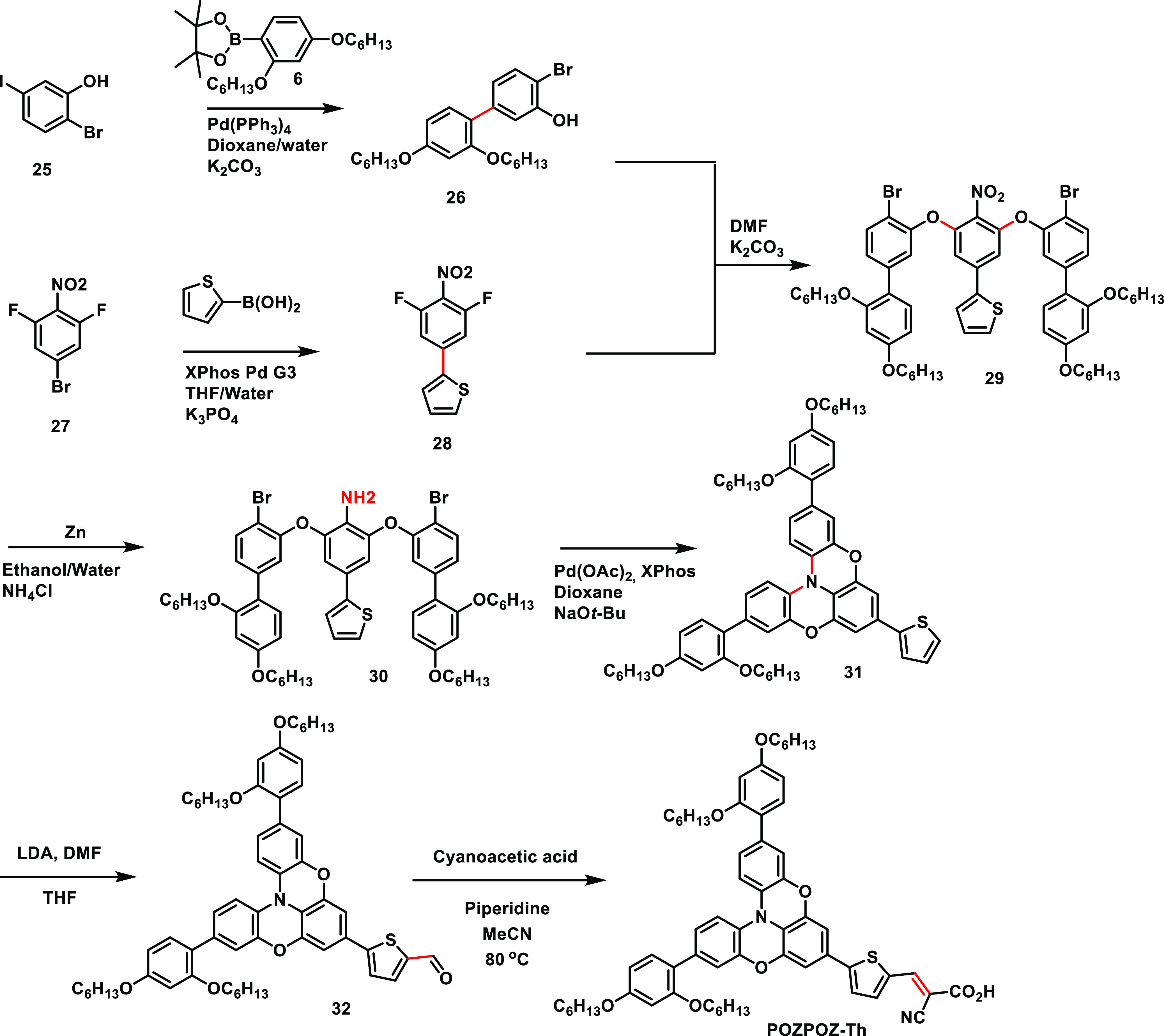
Synthesis of **POZPOZ-Th**

The final synthesis route for the successful preparation of **POZPOZ-Th** is shown in Scheme 5. First, phenol **26** was prepared in a similar manner to the previously mentioned intermediate **16**. In parallel, biaryl **28** was prepared in 74% yield using once more the conditions and precatalyst reported by Bruno et al.^43^ Phenol **26** was then coupled to the central ring fragment **28** in a nucleophilic aromatic substitution reaction and afforded intermediate **29** in a yield of 59%. *o*-Fluoro-nitrophenyl is a common motif for directing nucleophilic aromatic substitutions, as exemplified by the total synthesis of vancomycin recently reported by Moore et al.,^53^ where this motif is present on multiple occasions in the complex synthesis. The reduction of **29** to **30** went smoothly using Zn, and the only purification needed was the removal of inorganic solids. Using the previous conditions for intramolecular Buchwald–Hartwig coupling, we prepared the cyclized intermediate **31** in a yield of 39% over two steps from **29**. Because we chose a thiophene π-spacer for **POZPOZ-Th**, which is slightly more acidic than furan, we were able to generate the lithiated thiophene using LDA instead of *n*-BuLi. The lithiated intermediate of **31** was quenched with DMF, affording aldehyde **32** in a yield of 48%. To complete the synthesis of the double phenoxazine dye, we carried out a Knoevenagel condensation, isolating **POZPOZ-Th** in a yield of 84%.

### Photophysical Properties

**Figure 2:**
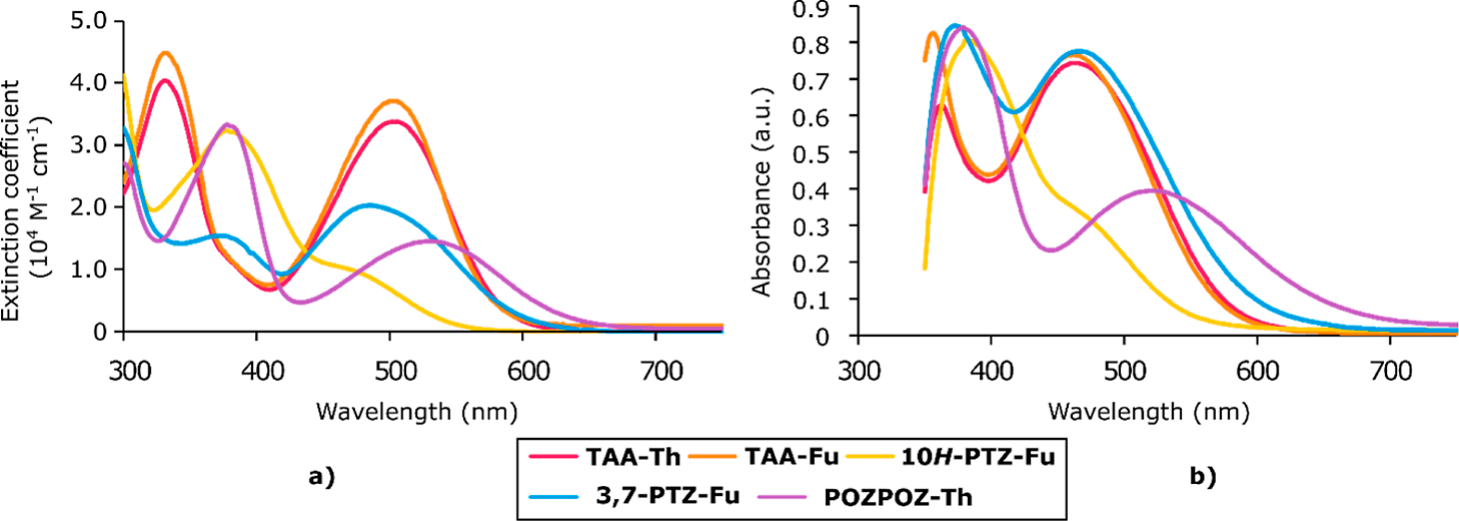
(a) UV/vis spectra of the dyes in dichloromethane (2 × 10^–5^ M). (b) UV/vis spectra of the dyes sensitized on a TiO_2_ film (2.5 μm, GreatcellSolar, 18NR-T).

The planarization of the triarylamine donor has a profound effect on the absorption properties of the dyes. To quantify this effect, we performed UV/vis measurements on the dyes in a solution of dichloromethane (2 × 10^–5^ M) and while adsorbed on a TiO_2_-film (2.5 μm). The results from these measurements are shown in Figure 2 and summarized in Table 1. The two triarylamine sensitizers, **TAA-Th** and **TAA-Fu**, display similar absorption properties in solution, albeit with a slightly higher molar extinction coefficient for the furan-linked dye. Comparing the absorption properties of the furan-linked dyes, we see that the absorption properties are adversely affected by the introduction of a sulfur bridge. The standard phenothiazine dye **3,7-PTZ-Fu**, is blue-shifted by 18 nm compared to **TAA-Fu**. For the symmetric phenothiazine dye, **10*H*-PTZ-Fu**, we assign the shoulder at ∼460 nm to stem from the charge-transfer absorption and a 42 nm blue shift, and a drastic reduction in molar extinction coefficient is observed. This blue shift is noted in several studies comparing 10*H*-phenothiazine dyes to conventional phenothiazine dyes^54^ and to triarylamine dyes.^55,56^ The absorption properties of dyes that adopt this configuration suffer from a poor orbital mixing between the donor and acceptor since the acceptor part of the conjugated system sits perpendicular to the phenothiazine donor.^56^ An example of the opposite is seen for the double phenoxazine dye, **POZPOZ-Th**, where more of the aromatic system of the donor is brought into the plane of the acceptor. The absorption maximum of this dye is red-shifted by 26 nm compared to its “free” triarylamine analogue, **TAA-Th**, proving that the double planarization of the triphenylamine donor is a suitable strategy for improving absorption properties, although the molar extinction coefficient is more than halved compared to the triarylamine analogue, **TAA-Th**.

**Table 1:** Photophysical and Electrochemical Properties of Dyes in the Series

dye	λ_abs_^a^ (nm)	ε (M^–1^ cm^–1^)	Em^b^ (nm)	λ_abs_^c^ on TiO_2_ (nm)	*E*_0-0_^d^(eV)	*E*_ox_^e^ (V vs SHE)	*E*_LUMO_^f^ (V vs SHE)
**TAA-Th**	504	33,800	639	464	2.24	1.13	–1.11
**TAA-Fu**	503	37,100	638	462	2.24	1.10	–1.14
**10*H*-PTZ-Fu**	461^g^	10,500^g^	591	461^g^	2.33	0.91	–1.42
**3,7-PTZ-Fu**	485	20,300	591	466	2.26	0.97	–1.29
**POZPOZ-Th**	530	14,500	639	522	2.11	0.96	–1.15

a Maximum of the most red-shifted peak.

b Emission when the ICT band is excited in DCM solution.

c Maximum of the most red-shifted peak on TiO_2_ (2.5 μm, GreatcellSolar 18NR-T).

d Calculated from the intersection of the absorption and normalized emission spectra.

e Measured vs F_c_^+^/F_c_ on stained TiO_2_ electrodes in acetonitrile with 0.1 M LiTFSI, converted to V vs SHE by 0.624 V. Scan rate 10 mV s^–1^.

f Calculated from *E*_ox_ – *E*_0–0_.

g Shoulder.

The absorption properties of the dyes on TiO_2_ revealed that all the dyes except **10*H*-PTZ-Fu** were blue-shifted upon absorption to TiO_2_. This blue-shift phenomenon is reported in the literature to be caused by a combination of deprotonation of the dye anchoring group upon attachment to the semiconductor and the formation of H-aggregated dye clusters on the surface of TiO_2_.^57,58^ To investigate this further, we obtained the absorption spectra of the dyes with 10 equiv anti-aggregation additive CDCA; the spectra are shown in Figure S1 in the Supporting Information. The spectra of the dyes with CDCA were fairly similar to the ones without CDCA but at a slightly lower intensity of absorption, which is likely attributed to a lower dye loading. The **10*H*-PTZ-Fu** dye displayed a similar absorption shoulder at the same wavelengths when adsorbed on TiO_2_ as when measured in solution. This is in accordance with the trend seen for the class of 10*H*-phenothiazine dyes,^40^ where this geometry is the only phenothiazine motif frequently associated with red shifts of absorption on TiO_2_ and the only geometry where the phenothiazine moieties can align to form *J*-aggregates when anchored on the surface. We also see that the conventional 3,7-phenothiazine geometry is less blue-shifted upon absorption on TiO_2_ than the triarylamine dyes, and in fact, it displays a slightly higher absorption maximum than the **TAA** dyes on TiO_2_. This could suggest that the 3,7-phenothiazine scaffold is less susceptible to form H-aggregates than triarylamines on the surface of titania.

### Electrochemical Properties

CV on stained TiO_2_ electrodes was performed for each dye in the series. The obtained voltammograms are displayed in Figure S2, and the energy levels of the frontier orbitals are shown in Figure 1c and Table 1. The properties of the two triarylamine dyes are nearly identical, and their oxidation potentials are found at 1.10–1.13 V versus SHE. Incorporating a sulfur bridge in a symmetrical manner (**10*H*-PTZ-Fu**) lowers the oxidation potential by 19 mV, and in an asymmetrical manner (**3,7-PTZ-Fu**), it is lowered by 13 mV compared to their reference dye **TAA-Fu**. The double phenoxazine dye (**POZPOZ-Th**) has a 17 mV lower oxidation potential than its triarylamine analogue, **TAA-Th**. Unfortunately, the planarization of the triarylamine donor renders the dyes incompatible with the *V*_OC_-enhancing copper-based electrolytes due to an insufficient driving force for the regeneration of the dye cations. A range of cobalt complexes has been reported with redox potentials of 0.43–0.85 V versus NHE,^37^ suggesting that a cobalt-based electrolyte could be used for the planarized dyes. However, the low molar extinction coefficients of **10H-PTZ-Fu**, **3,7-PTZ-Fu**, and **POZPOZ-Th** would not be optimal for the thin TiO_2_ layers required for the diffusion-limited cobalt redox shuttles. Hence, we opted for the traditional I^–^/I_3_^–^ redox shuttle for our photovoltaic evaluation of the sensitizers reported herein.

### Photovoltaic Properties

**Figure 3:**
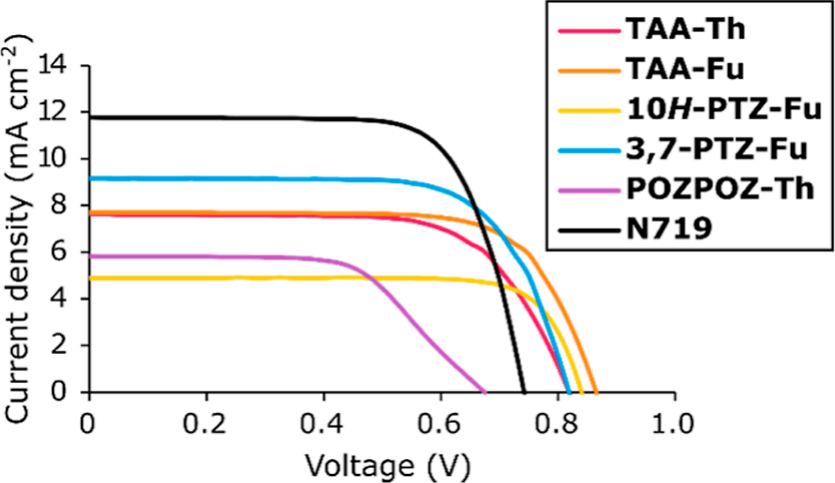
*J*–*V* curves of the best performing DSSC device for each dye and the reference sensitizer **N719**.

**Figure 4:**
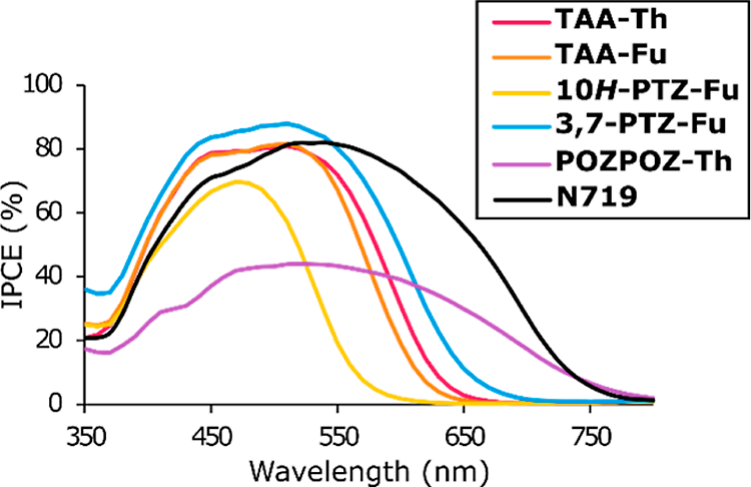
IPCE spectra of the best performing DSSC device for each dye and the reference sensitizer **N719**.

To evaluate the photovoltaic performance of the sensitizers, we prepared three DSSC devices for each dye, and the average photovoltaic parameters are presented in Table 2. The *J*–*V* curves of the best device for each dye are shown in Figure 3, and the obtained IPCE spectra from these devices are shown in Figure 4. The integrated IPCE spectra show consistently higher short-circuit currents than what was measured under 1 sun AM 1.5G illumination, meaning that the reported efficiencies do not fail our previously reported data credibility assessment.^40^ The triarylamine dyes revealed that the furan π-spacer is beneficial for boosting photovoltaic performance compared to a thiophene π-spacer. The *V*_oc_ of **TAA-Fu** was 40 mV larger than that of **TAA-Th**; this is consistent with the results from our previous study on π-spacers for phenothiazine dyes in the I^–^/I_3_^–^ electrolyte.^39^ The drop in photovoltage seen for dyes with thiophene linkers could be caused by the facilitation of recombination stemming from iodine–sulfur interactions occurring near the surface of TiO_2_.^59^ The significantly higher dye loading of **TAA-Fu** compared to **TAA-Th** could also provide better surface protection of TiO_2_, which would also retard recombination between electrons in the semiconductor and oxidants in the electrolyte. The IPCE spectrum of **TAA-Th** is in fact slightly wider than its furan counterpart and would therefore be expected to produce a larger photocurrent. Inconsistencies between IPCE and *J*–*V* measurements can be expected due to the IPCE measurements being carried out at lower intensities of irradiation and at single wavelengths.^60^

**Table 2:** Photovoltaic Performance of all Dyes under 1 sun AM 1.5G Illumination and from IPCE Measurements^a^

dye	IPCE *J*_SC_ (mA cm^–2^)^b^	*J*_SC_ (mA cm^–2^)^c^	*V*_OC_ (mV)^c^	FF^c^	PCE (%)^c^	dye loading (10^–8^ mol cm^–2^)^d^
**TAA-Th**	9.05	7.5 ± 0.2	829 ± 4	0.66 ± 0.01	4.1 ± 0.1	22 ± 1.4
**TAA-Fu**	8.43	7.7 ± 0.0	869 ± 4	0.70 ± 0.02	4.7 ± 0.1	31 ± 0.4
**10*H*-PTZ-Fu**	5.25	4.9 ± 0.0	839 ± 9	0.77 ± 0.01	3.1 ± 0.1	25 ± 0.4
**3,7-PTZ-Fu**	10.82	8.8 ± 0.2	838 ± 10	0.70 ± 0.00	5.2 ± 0.1	29 ± 0.5
**POZPOZ-Th**	7.56	5.8 ± 0.2	673 ± 0	0.59 ± 0.03	2.3 ± 0.1	33 ± 0.5
**N719**^e^	13.61	11.8	742	0.72	6.3	

a Results from dye loading experiments are also included.

b Otained by integration of the IPCE spectrum over the 1 sun AM 1.5 G spectrum.

c Average values of three separate devices.

d Values averaged of two desorbed TiO_2_ electrodes.

e Values from the best-performing device.

The effect of the sulfur bridge on photovoltaic performance is apparent when comparing the phenothiazine dyes and the furan-linked triarylamine dye. Although the **10*H*-PTZ-Fu** dye consistently produced the highest fill factors of this series, the severely reduced light harvesting ability of this dye meant that the overall performance was significantly reduced compared to the non-planarized triarylamine analogue. The sub-optimal performance of this dye was expected as this type of phenothiazine motif is associated with the worst overall performance of phenothiazine dyes.^40^ The conventional **3,7-PTZ-Fu** dye displayed improved light harvesting abilities compared to the triarylamine reference dye, **TAA-Fu**. As a result of this, the *J*_sc_ of **3,7-PTZ-Fu** was increased by 14% by planarizing the triarylamine dye with a sulfur bridge in an asymmetrical manner. Although the phenothiazine dye produced higher photocurrents, a 30 mV reduction in *V*_oc_ was also seen for the 3,7-phenothiazine dye. Still, the photovoltage of 3,7-PTZ-Fu was remarkably high for an I^–^/I_3_^–^ electrolyte and consistent with our previous report on the *N*-arylphenothiazine dye **DMA-0** achieving a similar photovoltage.^35^ The best photovoltaic performance of this series of dyes was in fact found for the conventional phenothiazine dye. This demonstrates that there is some merit to the phenothiazine donor over the triarylamine donor because of an increased light harvesting ability. It should be noted that this only holds when employing a traditional I^–^/I_3_^–^ electrolyte.

When considering the IPCE spectra shown in Figure 4, we see that the double oxygen bridge planarization of **POZPOZ-Th** leads to a panchromatic IPCE response. This dye produced an IPCE spectrum with absorption as wide as the reference sensitizer, **N719**, albeit at approximately halved intensity. The IPCE of a DSSC is a product of light harvesting efficiency, charge injection efficiency, and charge collection efficiency.^61^ Considering that the dye loading of **POZPOZ-Th** is the highest in this series and the molar extinction coefficient is superior to that of **N719** (1.3 × 10^4^ M^–1^ cm^–1^),^62^ it is likely that the fully planar dye suffers from poor charge injection or charge collection. The planar design should leave the dyes more susceptible to aggregate, which often promotes excited-state quenching and impaired charge injection.^63^ The less bulky design of **POZPOZ-Th** could also promote recombination of electrons in TiO_2_ with oxidants in the electrolyte, adversely affecting the charge collection efficiency. In any case, the fully planarized dye displayed the worst photovoltaic performance in the series, despite its excellent spectral coverage. Also worth noting is the strange behavior of the *J*–*V* curve of **POZPOZ-Th** where the slope changes around 550 mV.

### Electrochemical Impedance Spectroscopy

**Figure 5:**
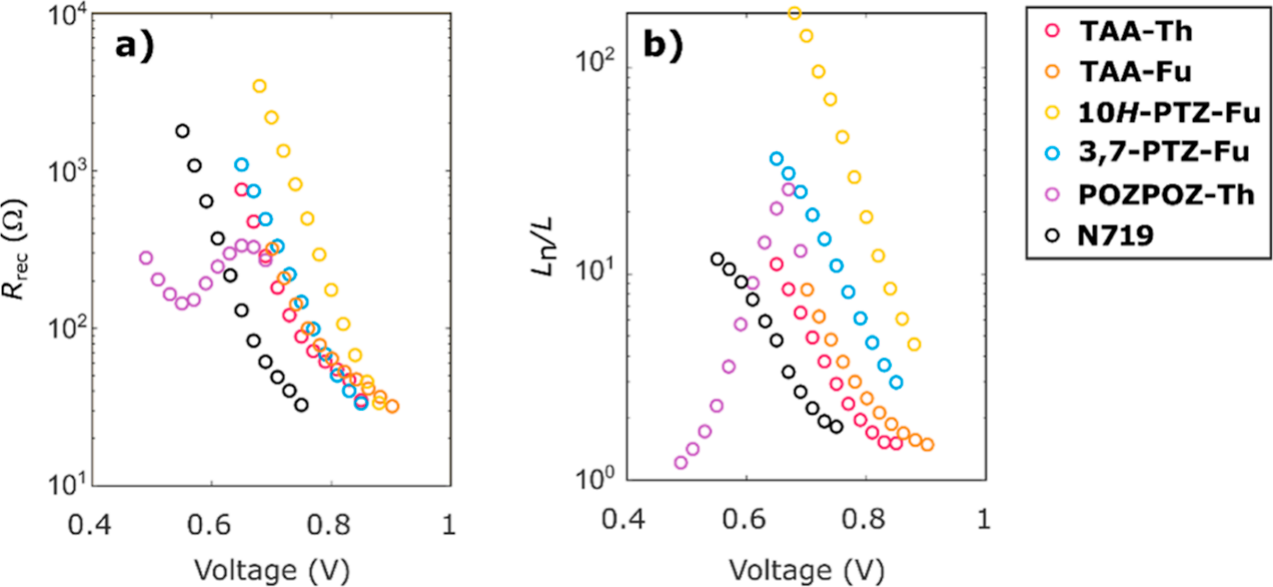
(a) Recombination resistance of the DSSC devices as a function of applied voltage. (b) Ratio of effective electron diffusion length to TiO_2_ film thickness as a function of applied voltage.

To examine the effect the planarization has on the surface passivation ability of the dyes, we performed electrochemical impedance spectroscopy (EIS) on the best-performing device fabricated for each dye. The obtained complex plane plots are shown in the Supporting Information, Figure S3, and from these, we extracted the recombination resistance, *R*_rec_, and plotted these versus applied voltage, as shown in Figure 5a. We also extracted the series resistance, *R*_s_, and the transport resistance, *R*_tr_; the plot of these as a function of applied voltage is shown in the Supporting Information, Figure S4. The recombination resistance plot, Figure 5a, shows that the lowest charge recombination is achieved by the symmetrical sulfur-bridged dye, **10*H*-PTZ-Fu**. This suggests that this geometry in fact produces a superior surface passivation compared to the triarylamine dyes. As the triarylamine class of dyes has gained much of its success from being superb blockers of electron recombination,^21^ the improved blocking ability of **10*H*-PTZ-Fu** is an impressive feature of this dye. The surface passivation properties of the 3,7-phenothiazine dye were also comparable to that of the triarylamine dyes. Meanwhile, the fully planarized double phenoxazine dye displayed an irregular and unexpected behavior, where the recombination resistance increased with increasing voltage from 0.55 V. This behavior is however in line with what we expect from the change of slope seen in the *J*–*V* curve of **POZPOZ-Th**. The relationship between recombination resistance and voltage is
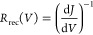
1where *V* is the voltage and *J* is the current density. The less steep slope of the *J*–*V* curve after 0.55 V is expected to affect the recombination resistance, as seen in Figure 5a. We attribute the irregular behavior and lower recombination resistance of **POZPOZ-Th** at voltages lower than *V*_OC_ to the more planar structure of this dye compared to the other dyes in the series. To further investigate the strange behavior of **POZPOZ-Th**, we looked at the effective electron diffusion length, *L*_n_, which is a measure of the competition between recombination and charge collection.^64^ The effective diffusion length is obtained readily from EIS measurements and is
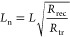
2where *L* is the film thickness, *R*_rec_ is the recombination resistance, and *R*_tr_ is the transport resistance. When considering the plot of the effective electron diffusion length in Figure 5b, we see that for all dyes except **POZPOZ-Th**, it decreases with increasing applied potential. In fact, the effective electron diffusion length of **POZPOZ-Th** approaches 1 at lower voltages, suggesting that we are approaching a device described by Gerischer impedance.^65^ This shows that for the fully planar dye, **POZPOZ-Th**, there is an unfavorable competition between charge collection and charge recombination. The incomplete electron collection likely contributes to the reduced IPCE intensity and in turn the low photocurrent produced by **POZPOZ-Th**. This is supported by the absorption intensity of the charge-transfer absorption of **POZPOZ-Th**, which is found to be slightly bigger than that of **10*H*-PTZ-Fu** when measured on TiO_2_, and the latter displays a considerably bigger IPCE intensity. Interestingly, Figure 5b also shows that both the phenothiazine dyes display longer effective electron diffusion lengths than the triarylamine analogues, showing that the sulfur bridges are useful for steering electrons toward collection instead of recombination with the redox shuttle.

## Conclusions

We have successfully demonstrated the synthesis of five novel triarylamine dyes with varying degrees of planarization in the donor moiety. We have shown that the planarization of the triarylamine donor has a profound effect on the electronic properties of the dyes, in addition to the geometrical and conformational effects of a more planar donor. A downside of this is the ambiguity when it comes to tracing changes in photovoltaic performance back to an electronic effect or a conformational effect or even a combination of the two. In any case, with evaluation of the “free” triarylamine dyes, **TAA-Th** and **TAA-Fu**, a clear performance advantage from using a furan π-spacer was noted. The furan-linked dye, **TAA-Fu**, produced an excellent photovoltage of 869 mV in an I^–^/I_3_^–^ electrolyte. When considering the sulfur-bridged dyes, **10H-PTZ-Fu** and **3,7-PTZ-Fu**, we saw some advantages associated with this type of planarization. Most notably, the dye **3,7-PTZ-Fu** displayed improved light harvesting ability and *J*_sc_ compared to its triarylamine analogue. The DSSC devices sensitized by this dye were the most efficient in this series of dyes at 5.2% (*J*_sc_ = 8.8 mA cm^–2^, *V*_oc_ = 838 mV, FF = 0.70). Moreover, EIS revealed that the symmetrical phenothiazine donor of **10H-PTZ-Fu** provided the highest recombination resistances and the longest effective diffusion lengths, which suggests that the geometry of this donor provides a better surface passivation than the propeller shape of triarylamines. Going to a fully planarized donor as in **POZPOZ-Th**, we show that this planarization produced a panchromatic IPCE spectrum. Alas, this planar donor provided a considerably worse charge collection than the other dyes in this series, as shown by the effective electron diffusion length measured by EIS. This has severe consequences for the photovoltaic performance of the dye **POZPOZ-Th**, and it proved to be the worst-performing dye in this series. Overall, we conclude that the out-of-plane geometry of “free” triarylamine is sub-optimal for light harvesting and that it is possible to improve this through planarization.

## Supporting Information Available

Cyclic voltammograms, complex plane plots from EIS, extracted transport resistance and series resistance data, experimental details related to the organic synthesis, and 1H and 13C NMR spectra of the reported compounds (PDF)
