# Supplementary material for: Synthetic Efforts to Investigate the Effect of Planarizing the Triarylamine Geometry in Dyes for Dye-Sensitized Solar Cells
Source: ACS Omega. 2022 Jun 16;7(25):22046–57. doi: 10.1021/acsomega.2c03163 (PMC9245111; doi:10.1021/acsomega.2c03163)
Supplement: Supplementary file 1 — ao2c03163_si_001.pdf [file ao2c03163_si_001.pdf]

Electronic Supplementary Information

Synthetic efforts to investigate the effect of planarizing the triarylamine geometry in dyes for dye-sensitized solar cells.

David Moe Almenningen [a], Veslemøy Minge Engh [a], Eivind Andreas Strømsodd [a], Henrik Erring Hansen [b], Audun Formo Buene [c], Bård Helge Hoff* [a], Odd Reidar Gautun* [a]

[a] Department of Chemistry, Norwegian University of Science and Technology, Høgskoleringen 5, 7491 Trondheim, Norway

* Corresponding author. E-mail address: bard.h.hoff@ntnu.no (B. H. Hoff).

* Corresponding author. E-mail address: odd.r.gautun@ntnu.no (O. R. Gautun).

[b] Department of Materials Science and Engineering, Norwegian University of Science and Technology, Sem Sælands vei 12, 7034 Trondheim, Norway

[c] Department of Civil and Environmental Engineering, Norwegian University of Science and Technology, Høgskoleringen 7a, 7491 Trondheim, Norway

List of contents

Cyclic voltammetry 2

Electrochemical impedance spectroscopy 3

Experimental 5

NMR 26

UV/Vis

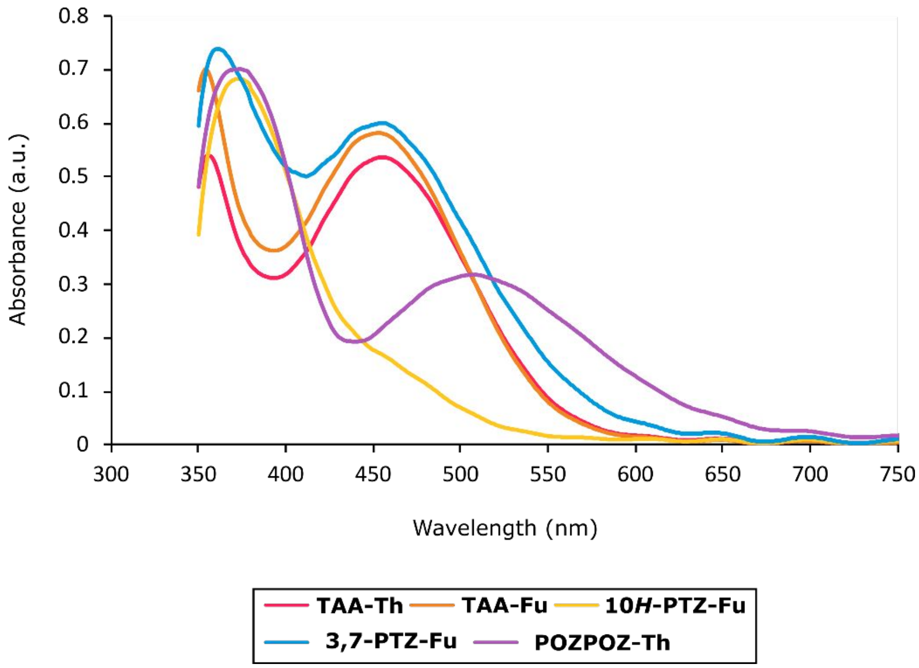

Figure S1 UV/Vis spectra of the dyes sensitized on a TiO_2 film with 10 eq. CDCA in the staining solution (2.5 µm, GreatcellSolar, 18NR-T).

Cyclic voltammetry

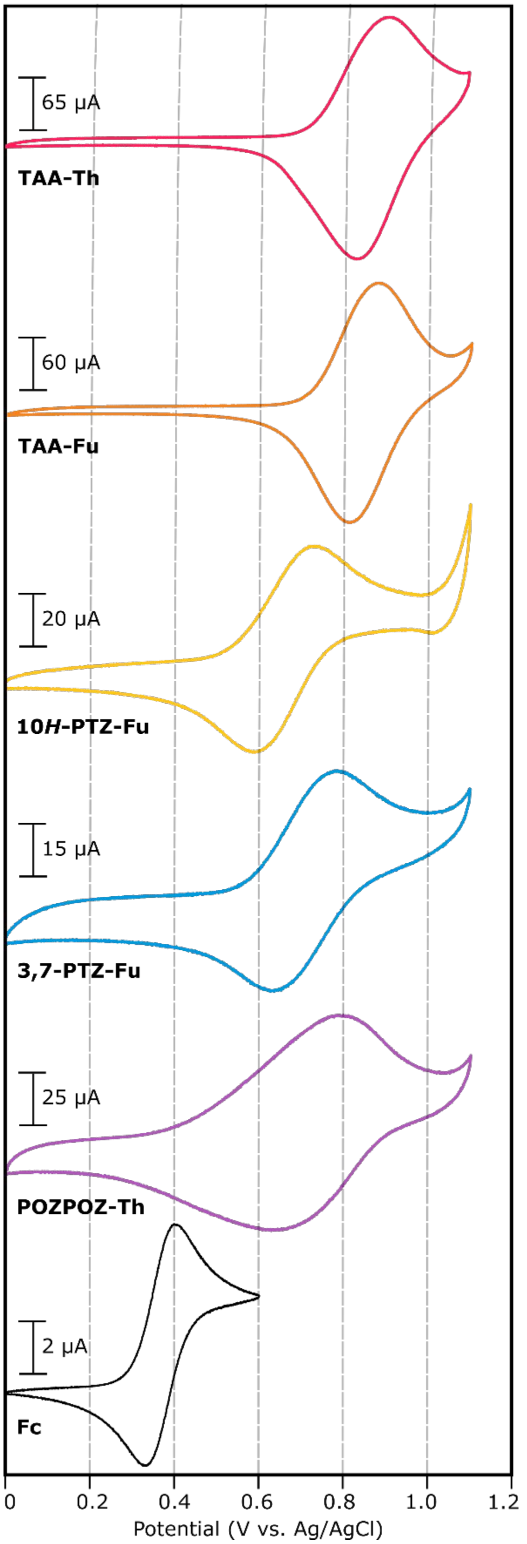

Figure S2 Cyclic voltammograms of the different dyes sensitized on TiO_2 electrodes.

Electrochemical impedance spectroscopy

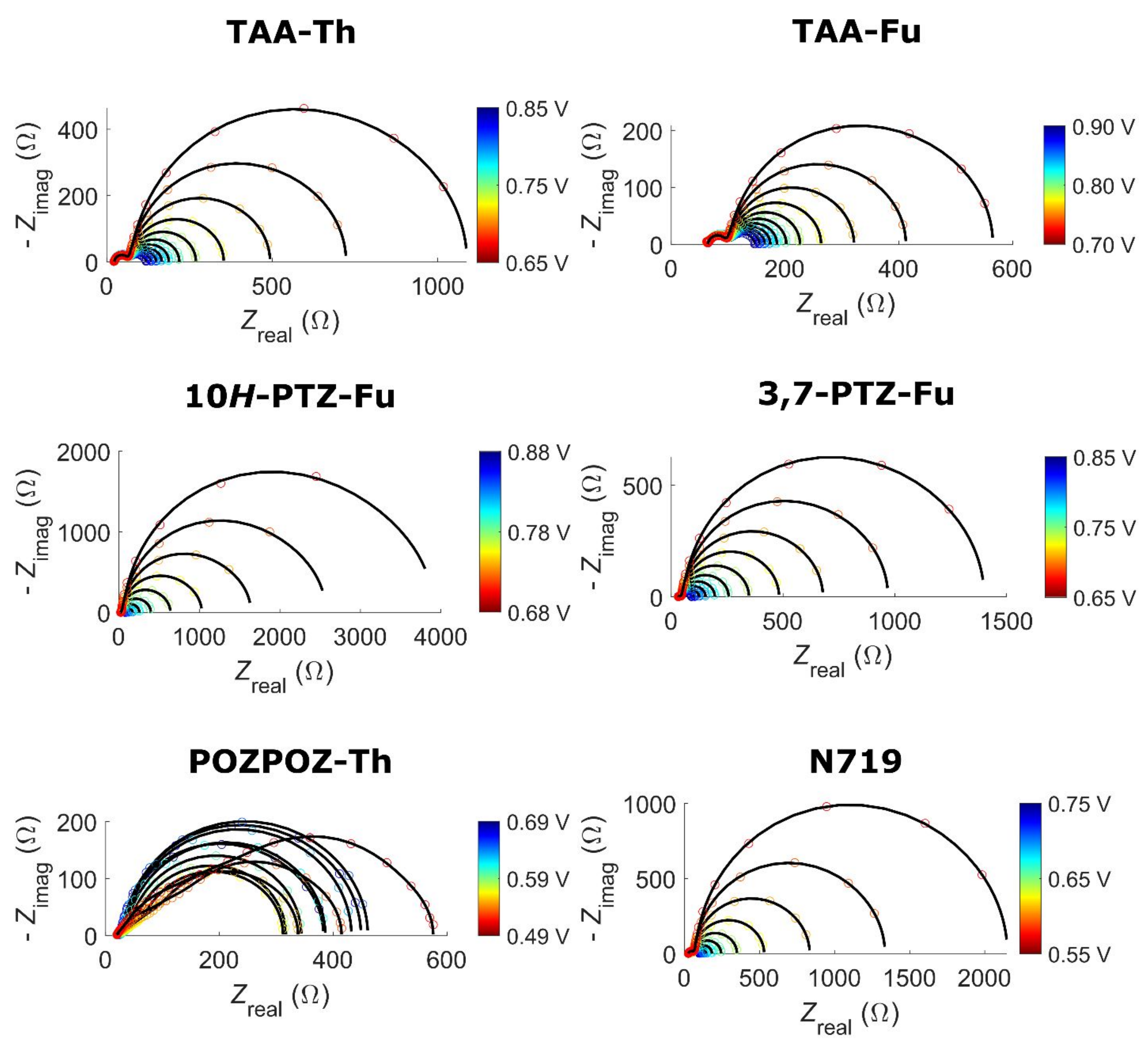

Figure S3 Complex plane diagrams obtained at different applied voltages.

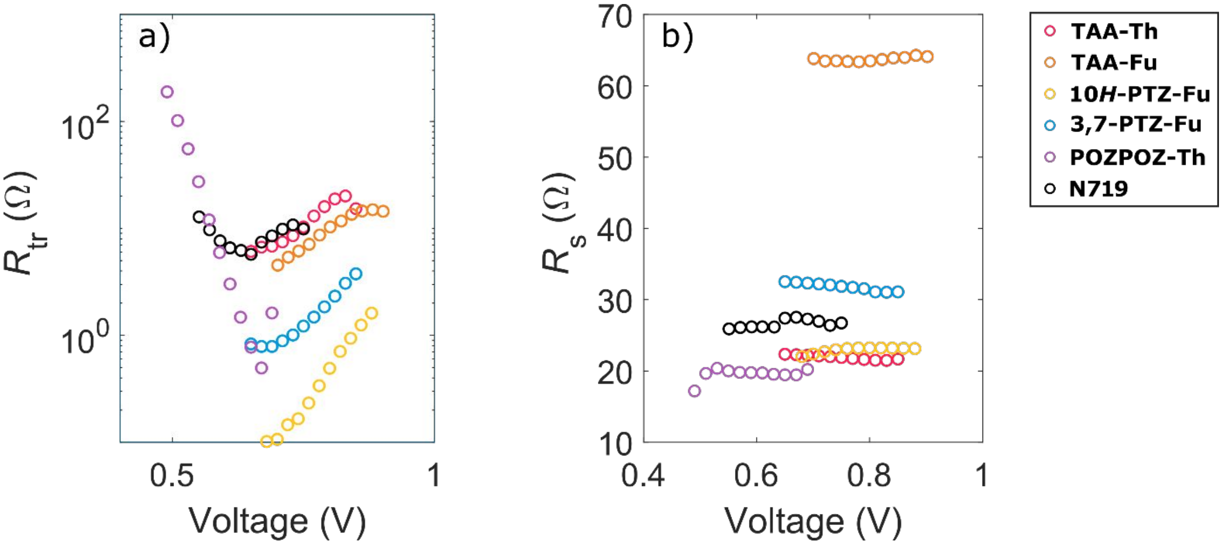

Figure S4 a) Transport resistance as a function of applied voltage. b) Series resistance as a function of applied voltage.

Experimental

Synthesis. All reactions were performed under inert N_2-atmosphere.

Analytical instruments. ^{1}H and ^{13}C NMR was performed using either a BRUKER 400 MHz or 600 MHz magnet, the spectra obtained are calibrated against residual solvent peak of $CDCl_3$ (7.26/77.16 ppm), CD_2Cl_2 (5.32/53.84 ppm), DMSO-d_6 (2.50/39.52 ppm), Acetone-d_6 (2.05/29.84 ppm) or THF-d_8 (1.72/25.31 ppm). The IR-spectra are obtained using a BRUKER Alpha Eco-ATR FTIR spectrometer, the data is reported with wavenumber and intensity of the signal. Mass determination was performed by MS-analysis in positive ionization mode on a Synapt G2-S Q-TOF-instrument for Waters, the samples were ionized by an ASAP-probe (APCI) without prior chromatographic separation. UV-Vis spectroscopy was performed using a Hitachi U-1900 spectrometer, and photoluminescence characterization was carried out on an Edinburgh Instruments FS5-spectrofluorometer.

5-(4-(bis(2',4'-bis(hexyloxy)-[1,1'-biphenyl]-4-yl)amino)phenyl)thiophene-2-carbaldehyde (2)

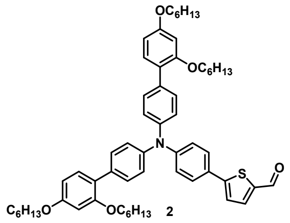

The triaryl bromide **1** (101 mg, 0.115 mmol), 5-formylthiophen-2-yl-boronic acid (28.9 mg, 0.185 mmol), XPhos Pd G3 (7.0 mg, 8.27×10^{-3} mmol) and K_3PO_4 (85.5 mg, 0.403 mmol) were mixed in a Schlenk-tube, before the reaction system was evacuated and filled with dry nitrogen gas. THF (2 mL) and water (4 mL) were degassed and added to the mixture. The reaction mixture was stirred at 40 °C for 4 h before cooling to 22 °C. Water (10 mL) and Et_2O (10 mL) were then added, and the aqueous and organic phases were separated. The aqueous phase was extracted with more Et_2O (3 × 10 mL). The combined organic phases were dried with anhydrous Na_2SO_4, filtered and the solvents removed in vacuo. The crude product (59.9 mg) was purified by silica-gel column chromatography (gradient: start: CH_2Cl_2/*n*-pentane, 1:3, R_f= 0.29, end: CH_2Cl_2) to yield **2** as yellow resin (41.8 mg, 0.0460 mmol, 40%). ^{1}H NMR (600 MHz, $CDCl_3$) δ: 9.85 (s, 1H), 7.70 (d, J = 3.9 Hz, 1H), 7.54-7.51 (m, 2H), 7.49-7.46 (m, 4H), 7.30 (d, J = 3.9 Hz, 1H), 7.27-7.25 (m,

2H), 7.19-7.14 (m, 6H), 6.56-6.52 (m, 4H), 3.99 (t, *J* = 6.6 Hz, 4H), 3.96 (t, *J* = 6.5 Hz, 4H), 1.83-1.71 (m, 8H), 1.52-1.23 (m, 24H), 0.92 (t, *J* = 7.0 Hz, 6H), 0.87 (t, *J* = 7.0 Hz, 6H); ^{13}C NMR (150 MHz, $CDCl_3$) δ: 182.6, 159.7 (2C), 157.0 (2C), 154.8, 149.3, 145.0 (2C), 141.2, 137.7, 134.1 (2C), 130.9 (2C),130.4 (4C), 127.2 (2C), 125.8, 124.5 (4C), 122.8 (2C), 122.7, 122.3 (2C), 105.4 (2C), 100.4 (2C), 68.4 (2C), 68.1 (2C), 31.6 (2C), 31.5 (2C), 29.3 (2C), 29.1 (2C), 25.78 (2C), 25.76 (2C), 22.63 (2C), 22.58 (2C), 14.1 (2C), 14.0 (2C); IR (EtOAc, cm^{-1}): 3354 (w), 3313 (w), 2924 (s), 2854 (s), 2655 (m), 2325 (m), 2051 (m), 1665 (s), 1597 (s), 1444 (s), 1291 (m), 1271 (m), 1181 (s), 1051 (s), 828 (m), 612 (s), 530 (s), 505 (s); HRMS (ASAP+, m/z): found 907.5206, calcd. for $C_{59}H_{73}NO_5S$ $[M^*]^+$ 907.5209.

5-(4-(bis(2',4'-bis(hexyloxy)-[1,1'-biphenyl]-4-yl)amino)phenyl)furan-2-carbaldehyde (3)

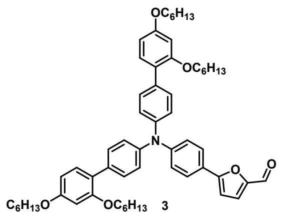

3

The synthesis of **3** was performed as described for compound **2** using 5-formylfuran-2-yl-boronic acid (27.7 mg, 0.198 mmol). Silica-gel column chromatography (gradient: start: CH_2Cl_2/*n*-pentane, 1:3, R_f= 0.26, end: CH_2Cl_2) gave (57.0 mg, 0.0639 mmol, 56%) of **3** as a yellow resin. ^{1}H NMR (600 MHz, $CDCl_3$) δ: 9.59 (s, 1H), 7.69-7.65 (m, 2H), 7.51-7.45 (m, 4H), 7.30 (d, *J* = 3.7 Hz, 1H), 7.27-7.25 (m, 2H), 7.17 (d, *J* = 8.6 Hz, 6H), 6.70 (d, *J* = 3.7 Hz, 1H), 6.56-6.52 (m, 4H), 3.99 (t, *J* = 6.6 Hz , 4H), 3.96 (t, *J* = 6.4 Hz, 4H), 1.84-1.71 (m, 8H), 1.51-1.22 (m, 24H), 0.92 (t, *J* = 7.0 Hz, 6H), 0.87 (t, *J* = 7.0 Hz, 6H); ^{13}C NMR (150 MHz, $CDCl_3$) δ: 176.7, 160.1, 159.7 (2C), 157.0 (3C), 151.6, 149.5, 145.0 (2C), 134.1 (2C), 130.9 (2C), 130.4 (4C), 126.3 (2C), 124.6 (4C), 122.8 (2C), 122.1 (2C), 121.7, 106.3, 105.4 (2C), 100.4 (2C), 68.4 (2C), 68.1 (2C), 31.6 (2C), 31.5 (2C), 29.3 (2C), 29.1 (2C), 25.8 (4C), 22.63 (2C), 22.59 (2C), 14.1 (2C), 14.0 (2C); IR (EtOAc, cm^{-1}): 2954 (s), 2926 (s), 2857 (s), 2662 (m), 1674 (s), 1601 (s), 1474 (s), 1323 (m), 1271 (m), 1182 (s), 1027 (m), 1003 (m), 835 (m), 518 (m); HRMS (ASAP+, m/z): found 892.5507, calcd. for $C_{59}H_{74}NO_6$ $[M+H]^+$ 892.5516.

(E)-3-(5-(4-(bis(2',4'-bis(hexyloxy)-[1,1'-biphenyl]-4-yl)amino)phenyl)thiophen-2-yl)-2-cyanoacrylic acid (TAA-Th)

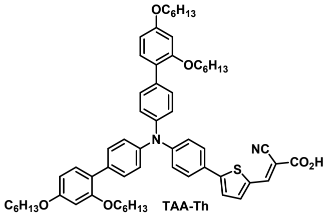

Compound **2** (36 mg, 0.0396 mmol) and cyanoacetic acid (79 mg, 0.926 mmol) were dissolved in degassed MeCN (15 mL) under nitrogen atmosphere. Piperidine (60.0 μL, 51.7 mg, 0.607 mmol) was added to the mixture, and the reaction was stirred at 80 °C for 1.5 h before cooling to 22 °C. The reaction mixture was then quenched in aqueous HCl (4M, 25 mL). CH_2Cl_2 (100 mL) was added and the organic phase was washed with water (6 × 100 mL), then dried over anhydrous Na_2SO_4, filtered and the solvents were removed in vacuo. The crude product (44.8 mg) was purified by silica-gel column chromatography (gradient: 0-20% MeOH in CH_2Cl_2, R_f = 0.00 in CH_2Cl_2). The dye-containing fractions were washed with HCl (1M, 2 × 20 mL), dried over anhydrous Na_2SO_4 and filtered to obtain (37 mg, 0.0383 mmol, 95%) of **TAA-Th** as a dark purple solid, mp. 62.1-67.5 °C. ^{1}H NMR (600 MHz, $CDCl_3$) δ: 8.32 (s, 1H), 7.78 (d, *J* = 4.0 Hz, 1H), 7.56 (d, *J* = 8.5 Hz, 2H), 7.49 (d, *J* = 8.6 Hz, 4H), 7.34 (d, *J* = 4.0 Hz, 1H), 7.28-7.24 (m, 2H), 7.18 (d, *J* = 8.6 Hz, 4H), 7.15 (d, *J* = 8.8 Hz, 2H), 6.57-6.52 (m, 4H), 3.99 (t, *J* = 6.5 Hz, 4H), 3.97 (t, *J* = 6.4 Hz, 4H), 1.84-1.71 (m, 8H), 1.52-1.22 (m, 24H), 0.92 (t, *J* = 7.0 Hz, 6H), 0.87 (t, *J* = 7.0 Hz, 6H); ^{13}C NMR (150 MHz, $CDCl_3$) δ: 168.1, 159.7 (2C), 157.0 (2C), 156.9, 149.8, 147.9, 144.8 (2C), 140.5, 134.3 (2C), 133.5, 130.9 (2C), 130.4 (4C), 127.4 (2C), 125.1, 124.7 (4C), 123.3, 122.7 (2C), 121.9 (2C), 115.9, 105.4 (2C), 100.4 (2C), 95.2, 68.4 (2C), 68.1 (2C), 31.6 (2C), 31.5 (2C), 29.3 (2C), 29.1 (2C), 25.78 (2C), 25.76 (2C), 22.62 (2C), 22.59 (2C), 14.0 (4C); IR (EtOAc, cm^{-1}): 2954 (m), 2928 (s), 2857 (m), 2321 (w), 2217 (w), 2069 (w), 1686 (m), 1576 (s), 1493 (s), 1412 (s), 1323 (m), 1281 (s), 1222 (m), 1183 (s), 1063 (m), 599 (m), 480 (m); HRMS (ASAP+, m/z): found 975.5330, calcd. for $C_{62}H_{75}N_2O_6S$ $[M+H]^+$ 975.5346.

(E)-3-(5-(4-(bis(2',4'-bis(hexyloxy)-[1,1'-biphenyl]-4-yl)amino)phenyl)furan-2-yl)-2-cyanoacrylic acid (TAA-Fu)

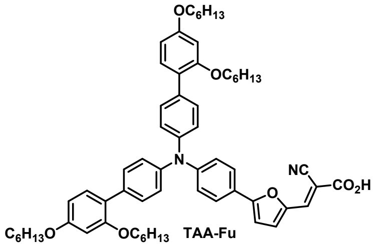

Synthesis and purification of **TAA-Fu** were performed as described for **TAA-Th** starting with intermediate **3** (52.6 mg, 0.059 mmol). This gave 42.3 mg (0.0441 mmol, 75%), of a dark purple solid, mp. 62.4-68.9 °C. ^{1}H NMR (600 MHz, $CDCl_3$) δ: 7.95 (s, 1H), 7.73 (d, *J* = 8.9 Hz, 2H), 7.51-7.47 (m, 4H), 7.28-7.24 (m, 2H), 7.21-7.16 (m, 6H), 6.81 (d, *J* = 3.9 Hz, 1H), 6.57-6.52 (m, 4H), 3.99 (t, *J* = 6.7, 4H), 3.97 (t, *J* = 6.6 Hz, 4H), 1.82-1.71 (m, 8H), 1.52-1.22 (m, 24H), 0.92 (t, *J* = 7.0 Hz, 6H), 0.86 (t, *J* = 7.0 Hz, 6H); ^{13}C NMR (150 MHz, $CDCl_3$) δ: 168.5, 161.8, 159.7 (2C), 157.0 (2C), 150.0, 147.2, 144.7 (2C), 138.6, 134.4 (2C), 130.9 (2C), 130.5 (4C), 126.8 (2C), 124.9 (4C), 122.8 (2C), 121.6 (2C), 120.8 (2C), 115.9, 108.2, 105.4 (2C), 100.4 (2C), 93.6, 68.4 (2C), 68.1 (2C), 31.6 (2C), 31.4 (2C), 29.3 (2C), 29.1 (2C), 25.77 (2C), 25.75 (2C), 22.62 (2C), 22.58 (2C), 14.1 (2C), 14.0 (2C); IR (EtOAc, cm^{-1}): 3270 (w), 3239 (w), 3087 (w), 2956 (s), 2930 (s), 2860 (m), 1591 (s), 1494 (s), 1470 (s), 1268 (m), 1182 (m), 824 (m), 792 (m); HRMS (ASAP+, m/z): found 957.5399, calcd. for $C_{62}H_{73}N_2O_7$ $[M-H]^+$ 957.5418.

1-(3,7-dibromo-10*H*-phenothiazin-10-yl)ethan-1-one (5)

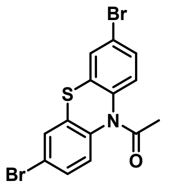

5

3,7-Dibromo-10*H*-phenothiazine (**4**) (1.05 g, 2.94 mmol) was suspended in dry toluene (30 mL). Acetyl chloride (0.461 g, 5.88 mmol) was added under a nitrogen atmosphere and the reaction mixture was then stirred for 3 h at reflux. The solvent was removed and the crude product washed with cold hexane to give **5** as a green powder (1.01 g, 2.52 mmol, 83%), mp. 138.5 °C. ^{1}H NMR (400 MHz, DMSO-d_6) δ: 7.83 (d, *J* =2.0 Hz, 2H), 7.60 (dd, *J* = 8.5, 2.1 Hz, 2H), 7.56 (d, *J* = 8.5

Hz, 2H), 2.13 (s, 3H); ^{13}C NMR (100 MHz, DMSO-d_6) δz: 168.8, 138.2 (2C), 134.4 (2C), 130.2 (2C), 139.6 (2C), 129.5 (2C), 119.9 (2C), 23.1; IR (neat, cm^{-1}): 3026, 1683, 1568, 1459, 1356, 1301, 1253, 1018 and 818; HRMS (ASAP+, m/z): found 399.8834, calcd. for $C_{14}H_{10}NOS^{81}Br_2$ $[M+H]^+$ 399.8829.

1-(3,7-bis(2,4-bis(hexyloxy)phenyl)-10*H*-phenothiazin-10-yl)ethan-1-one (7)

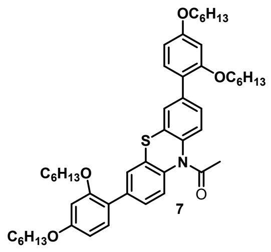

Formation of pinacol borane: A mixture of 1-bromo-2,4-bis(hexyloxy)benzene (2.67 mg, 7.52 mmol mmol), $PdCl_2(CH_3CN)_2$ (67.3 mg, 259 μmol) and SPhos (199 mg, 485 μmol) was evacuated before a nitrogen atmosphere was established. Degassed and dry 1,4-dioxane (23 mL) was then added and the reaction mixture was stirred for 20 min. Then dry triethylamine (2.46 g, 24.4 mmol) and 4,4,5,5-tetramethyl-1,3,2-dioxoborolane (1.92 mg, 15.0 mmol were added and the reaction mixture stirred at 105 °C for 90 min. The reaction mixture was then cooled down and filtered through Celite, before the solvents were removed *in vacuo* to give crude 2-(2,4-bis(hexyloxy)phenyl)-4,4,5,5-tetramethyl-1,3,2-dioxaborolane as a yellow oil, which was then used directly in the Suzuki cross-coupling reaction.

Suzuki cross-coupling: Crude **6** (1.19 g, 2.98 mmol), $Pd(OAc)_2$ (35.0 mg, 160 μmol), SPhos (126 mg, 307 μmol) and K_2CO_3 (2.06 g, 14.9 mmol) were mixed with 2-(2,4-bis(hexyloxy)phenyl)-4,4,5,5-tetramethyl-1,3,2-dioxaborolane from above, before the mixture was evacuated and a nitrogen atmosphere established. Degassed H_2O (20 mL) and 1,4-dioxane (20 mL) were added under a nitrogen atmosphere. This mixture was stirred at 80 °C for 6 h. After the mixture was cooled down to rt, H_2O (75 mL) was added and the aqueous phase extracted with CH_2Cl_2 (4 × 75 mL). The combined organic phase was washed with H_2O (2 × 50 mL), dried over Na_2SO_4 before the solvents were removed in vacuo. The crude product was purified twice with column chromatography (*n*-pentane/EtOAc, 17:3, R_f = 0.25) to yield **7** (1.54 g, 1.93 mmol, 65%) as an orange oil. ^{1}H NMR (600 MHz, DMSO-d_6, 80 °C) δ: 7.65-7.59 (m, 4H), 7.45 (dd, J = 2.0, 8.4 Hz, 2H), 7.26 (d, J = 8.4 Hz, 2H), 6.62 (d, J = 2.4 Hz, 2H), 6.60 (dd, J = 8.5, 2.3 Hz, 2H), 4.02-3.94

(m, 8H), 2.19 (s, 3H), 1.77-1.70 (m, 4H), 1.67-1.61 (m, 4H), 1.47-1.39 (m, 4H), 1.39-1.27 (m, 12H), 1.27-1.18 (m, 8H), 0.88 (t, $J = 6.7$ Hz, 6H), 0.77 (t, $J = 6.7$ Hz, 6H); ^{13}C NMR (150 MHz, DMSO-d_6, 80 °C) δ: 168.0, 159.6 (2C), 156.4 (2C), 136.6 (2C), 136.5 (2C), 131.1 (2C), 130.3 (2C), 127.6 (2C), 127.3 (2C), 126.2 (2C), 120.8 (2C), 106.3 (2C), 100.4 (2C), 68.0 (2C), 67.5 (2C), 30.50 (2C), 30.45 (2C), 28.29 (2C), 28.20 (2C), 24.9 (2C), 24.7 (2C), 22.1, 21.49 (2C), 21.45 (2C), 13.2 (2C), 13.1 (2C); IR (CH_2Cl_2, cm^{-1}): 2928, 2857, 1682, 1608, 1499, 1301, 1277, 1181; HRMS (ASAP+, m/z): found 794.4808, calcd. for $C_{50}H_{68}NO_5S$ $[M+H]^+$ 794.4818.

3,7-bis(2,4-bis(hexyloxy)phenyl)-10H-phenothiazine (8)

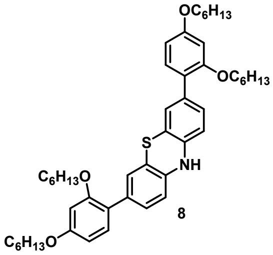

Compound **7** (1.52 g, 1.91 mmol) was dissolved in THF (8 mL) and MeOH (20 mL), before this solution was degassed and added under a nitrogen atmosphere to a flask containing NaOH (319 mg, 7.98 mmol). This mixture was then stirred for 2 h at 60 °C, before being cooled down to 22 °C. Water (50 mL) was added and the aqueous phase extracted with CH_2Cl_2 (4 × 50 mL). The organic phases were combined and washed with saturated $NaHCO_3$ (3 × 50 mL) and H_2O (4 × 50 mL) before being dried over Na_2SO_4. The solvents were removed in vacuo to give **8** (1.34 g, 1.78 mmol, 93%) as a green solid; mp. 85-89 °C. ^{1}H NMR (600 MHz, DMSO-d_6) δ: 8.62 (s, 1H), 7.13 (d, $J = 8.2$ Hz, 2H), 7.08-7.05 (m, 2H), 7.05-7.01 (m, 2H), 6.69 (d, $J = 8.2$ Hz, 2H), 6.58-6.54 (m, 2H), 6.54-6.49 (m, 2H), 4.00-3.88 (m, 8H), 1.73-1.66 (m, 4H), 1.66-1.58 (m, 4H), 1.46-1.34 (m, 8H), 1.34-1.20 (m, 16H), 0.88 (t, J = 6.7 Hz, 6H), 0.84 (t, J = 6.7 Hz, 6H); ^{13}C NMR (150 MHz, DMSO-d_6) δ: 159.0 (2C), 156.3 (2C), 140.2 (2C), 131.5 (2C), 130.0 (2C), 128.0 (2C), 126.7 (2C), 121.4 (2C), 115.6 (2C), 113.9 (2C), 105.8 (2C), 100.1 (2C), 67.7 (2C), 67.5 (2C), 31.00 (2C), 30.97 (2C), 28.7 (2C), 28.6 (2C), 25.4 (2C), 25.2 (2C), 22.10 (2C), 22.06 (2C), 13.88 (2C), 13.86 (2C); IR (CH_2Cl_2, cm^{-1}): 3374 (w), 2954 (m), 2931 (m), 2867 (m), 1604 (m), 1576 (w), 1487 (m), 1287 (m), 1269 (m), 1257 (m), 1184 (m), 1040 (w), 1019 (w), 819 (m); HRMS (ASAP+, m/z): found 752.4700, calcd. for $C_{48}H_{66}NO_4S$, $[M+H]^+$, 752.4713.

3,7-bis(2,4-bis(hexyloxy)phenyl)-10-(4-(trimethylsilyl)phenyl)-10*H*-phenothiazine (9)

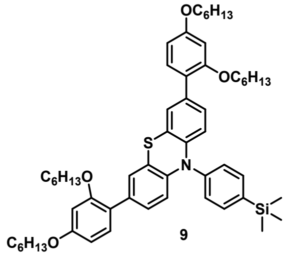

9

Compound **8** (764 mg, 1.06 mmol), NaO-*t*Bu (137 mg, 1.42 mmol), XPhos (48.4 mg, 102 mmol) and $Pd(OAc)_2$ (11.4 mg, 50.8 mmol) were mixed and degassed 1,4-dioxane (5 mL) was added under nitrogen atmosphere, before this mixture was left for stirring at rt for a couple of minutes. 1-Bromo-4(trimethylsilyl)benzene (704 mg, 3.07 mmol) was then added, and the reaction mixture was heated to 110 °C and left stirring for 15 h. The reaction mixture was then cooled down to rt, H_2O (100 mL) was added and the crude product extracted with CH_2Cl2 (4 × 75 mL). The organic phases were combined, washed with H_2O (2 × 100 mL), dried over Na2SO4 and filtrated before the solvents were removed *in vacuo*. Purification by silica-gel column chromatography (*n*-pentane/CH_2Cl_2, 1:3, R*f* = 0.20) gave **9** (828 mg, 0.920 mmol, 87%) as a green oil. ^{1}H NMR (600 MHz, CD_2Cl_2) δ: 7.78 (d, *J* = 8.0 Hz, 2H), 7.40 (d, *J* = 8.1 Hz, 2H), 7.22 (d, *J* = 1.9 Hz, 2H), 7.15 (d, *J* = 8.1 Hz, 2H), 7.02 (dd, *J* = 8.4, 1.9 Hz, 2H), 6.52-6.48 (m, 4H), 6.24 (d, *J* = 8.6 Hz, 2H), 3.96 (t, *J* = 6.7 Hz, 4H), 3.93 (t, *J* = 6.4 Hz, 4H), 1.81-1.69 (m, 8H), 1.50-1.40 (m, 8H), 1.40-1.28 (m, 16H), 0.93 (t, *J* = 6.9 Hz, 6H), 0.88 (t, *J* = 6.8 Hz, 6H), 0.36 (s, 9H); ^{13}C NMR (150 MHz, CD_2Cl_2) δ: 160.1 (2C), 157.3 (2C), 142.8 (2C), 142.2, 141.0, 136.1 (2C), 133.3 (2C), 130.8 (2C), 129.8 (2C), 128.1 (2C), 127.8 (2C), 122.3 (2C), 120.2 (2C), 116.2 (2C), 105.8 (2C), 100.5 (2C), 68.8 (2C), 68.6 (2C), 32.03 (2C), 31.97 (2C), 29.7 (2C), 29.6 (2C), 26.3 (2C), 26.1 (2C), 23.0 (4C), 14.3 (2C), 14.2 (2C), -1.0 (3C); IR (CH_2Cl_2, cm^{-1}): 2954, 2930, 2859, 1608, 1466, 1297, 1248, 1179, 1135, 1077, 1017, 832, 662, 602, 537; HRMS (ASAP+, m/z): found 899.5332, calcd. for $C_{57}H_{77}NO_4SiS$, $[M^*]^+$ 899.5343

3,7-bis(2,4-bis(hexyloxy)phenyl)-10-(4-iodophenyl)-10*H*-phenothiazine (10)

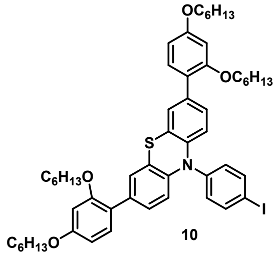

10

Compound **9** (693 mg, 0.770 mmol) was dissolved in dry CH_2Cl_2 (16 mL) and dry acetonitrile (16 mL) and left stirring at 0 °C for 5 min. Then a solution of ICl in CH_2Cl_2 (1.88 mL, 0.6 M) was added at 0 °C and the reaction was left stirring at 22°C for 20 h. The reaction was then quenched with an aqueous solution of $Na_2S_2O_3$ (5 wt%, 40 mL) and the mixture was extracted with CH_2Cl_2 (2 × 60 mL). The combined organic phases were washed with H_2O (30 mL) , dried over Na_2SO_4, filtered and the solvents removed *in vacuo*. The product was purified by silica-gel column chromatography (*n*-pentane/acetone, 20:1, R_f = 0.15) to yield **10** (137 mg, 0.144 mmol, 19%) as a yellow oil. ^{1}H NMR (600 MHz, acetone-d_6) δ: 8.01 (d, *J* = 8.6 Hz, 2H), 7.30 (d, *J* = 2.0 Hz, 2H), 7.26 (d, *J* = 8.5 Hz, 2H), 7.18 (d, *J* = 8.4 Hz, 2H), 7.10 (dd, *J* = 8.5, 2.0 Hz, 2H), 6.59 (d, *J* = 2.2 Hz, 2H), 6.54 (dd, *J* = 8.5, 2.3 Hz, 2H), 6.33 (d, *J* = 8.5 Hz, 2H), 4.02-3.96 (m, 8H), 1.81-1.67 (m, 8H), 1.52-1.42 (m, 8H), 1.40-1.27 (m, 16H), 0.90 (t, *J* = 7.1 Hz, 6H), 0.87 (t, *J* = 6.9 Hz, 6H); ^{13}C NMR (150 MHz, acetone-d_6) δ: 160.9 (2C), 157.8 (2C), 142.7 (2C), 142.5, 140.9 (2C), 134.5 (2C), 132.9 (2C), 131.2 (2C), 128.7 (2C), 128.5 (2C), 122.5 (2C), 121.4 (2C), 117.4 (2C), 106.7 (2C), 101.0 (2C), 93.2, 69.0 (2C), 68.6 (2C), 32.4 (4C), 30.04 (2C), 30.00 (2C), 26.8 (2C), 26.5 (2C), 23.4 (2C), 23.3 (2C), 14.4 (2C), 14.3 (2C); IR (CH_2Cl_2, cm^{-1}): 2953, 2929, 2868, 2858, 1608, 1455, 1388, 1298, 1181, 1135, 1053, 1010, 833, 816, 602, 532; HRMS (ASAP+, m/z): found 953.3906, calcd. for $C_{54}H_{68}NO_4SI$, $[M^*]^+$ 953.3914.

3,7-bis(2,4-bis(hexyloxy)-5-iodophenyl)-10-(4-iodophenyl)-10H-phenothiazine (10-by)

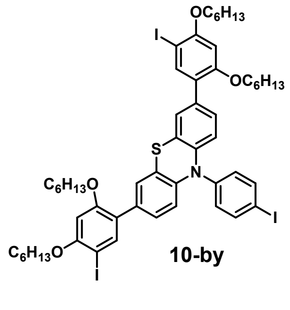

10-by

The tri-iodinated by-product **10-by** was isolated in a synthesis as described for compound **10**, but starting with **9** (897 mg, 0.996 mmol) and more ICl (485 mg, 2.99 mmol). Two purifications using silica-gel column chromatography (*n*-pentane/ CH_2Cl_2, 1:2, $R_f = 0.35$) gave **10-by** (193 mg, 0.160 mmol, 16%) as a yellow solid, mp. 145 °C. ^{1}H NMR (600 MHz, CD_2Cl_2) δ: 7.95 (d, J = 8.5 Hz, 2H), 7.60 (s, 2H), 7.23-7.16 (m, 4H), 7.00 (br.d, J = 5.2 Hz, 2H), 6.47 (s, 2H), 6.25 (br.d, J = 5.8 Hz, 2H), 4.03 (t, J = 6.5 Hz, 4H), 3.95 (t, J = 6.4 Hz, 4H), 1.87-1.79 (m, 4H), 1.76-1.68 (m, 4H), 1.58-1.50 (m, 4H), 1.46-1.28 (m, 20H), 0.92 (t, J = 7.2 Hz, 6H), 0.88 (t, J = 7.0 Hz, 6H); ^{13}C NMR (150 MHz, CD_2Cl_2) δ: 158.3 (2C), 157.8 (2C), 140.5 (2C), 139.59 (2C), 139.57, 132.6 (2C), 128.1 (2C), 127.8 (2C), 124.3 (2C), 116.4 (2C), 115.8 (2C), 98.7 (2C), 93.3, 75.3 (2C), 70.0 (2C), 69.2 (2C), 31.92 (2C), 31.90 (2C), 29.51 (2C), 29.50 (2C), 26.23 (2C), 26.13 (2C), 14.25 (2C), 14.22 (2C); IR (CH_2Cl_2, cm^{-1}): 2950, 2925, 2867, 2856, 1590, 1460, 1359, 1302, 1244, 1187, 1040, 1009, 814, 538; HRMS (ASAP+, m/z): found 1205.1830 calc for $C_{54}H_{66}I_3NO_4S$, $[M^*]^+$ 1205.1847.

5-(4-(3,7-bis(2,4-bis(hexyloxy)phenyl)-10*H*-phenothiazin-10-yl)phenyl)furan-2-carbaldehyde (11)

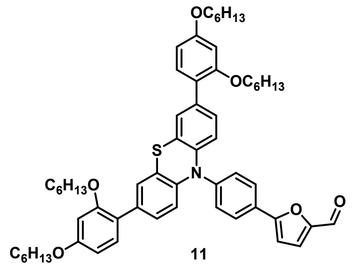

11

(5-Formylfuran-2-yl)boronic acid (31.0 mg, 0.221 mmol, 1.6 equiv), K_3PO_4 (59.0 mg, 0.278 mmol), and XPhos Pd G3 (2.30 mg, 2.73 μmol) were mixed in a Schlenk tube. Then compound **10** (132 mg, 0.138 mmol) was dissolved in degassed THF (2.5 mL) and added to the mixture, followed by degassed H_2O (5 mL). The reaction mixture was stirred at 40 °C for 23 h before being cooled down to rt, followed by addition of H_2O (20 mL).The mixture was then extracted using CH_2Cl_2 (4 × 20 mL), and the organic phases washed with H_2O (40 mL). After drying over Na_2SO_4, and concentration in vacuo, the material was purified by silica-gel column chromotography (n-pentane/acetone, 1:8, $R_f = 0.15$) to yield **11** (55.0 mg, 0.0596 mmol, 43%) as a yellow oil. ^{1}H NMR (600 MHz, acetone-*d*$_6$) δ: 9.70 (s, 1H), 8.13-8.11 (m, 2H), 7.56 (d, J = 3.7 Hz, 1H), 7.56-7.53 (m, 2H), 7.37 (d, J = 2.1 Hz, 2H), 7.24 (d, J = 3.8 Hz, 1H), 7.20 (d, J = 8.4 Hz, 2H), 7.16 (dd, J = 8.5, 2.1 Hz, 2H), 6.61 (d, J = 2.2 Hz, 2H), 6.57-6.52 (m, 4H), 4.03-3.98 (m, 8H), 1.80-1.68 (m, 8H),

1.57-1.43 (m, 8H), 1.38-1.27 (m, 16H), 0.90 (t, J = 7.1 Hz, 6H), 0.86 (t, J = 7.0 Hz, 6H); ^{13}C NMR (150 MHz, acetone-d_6) δ: 178.0, 160.9 (2C), 158.9, 157.9 (2C), 153.5, 144.1, 142.5 (2C), 134.9 (2C), 131.3 (2C), 129.5 (2C), 128.76 (2C), 128.71, 128.60 (2C), 128.23 (2C), 124.8, 122.94 (2C), 122.45 (2C), 118.6 (2C), 109.3, 106.7 (2C), 101.0 (2C), 69.0 (2C), 68.6 (2C), 32.4 (4C), 30.0 (4C)*, 26.8 (2C), 26.5 (2C), 23.35 (2C), 23.30 (2C), 14.40 (2C), 14.32 (2C); IR (CH_2Cl_2, cm^{-1}): 2954, 2930, 2867, 2856, 1679, 1608, 1467, 1158, 1137, 1121, 1077, 1016, 822, 625, 603, 512; HRMS (ASAP+, m/z): found 921.4983, calcd. for $C_{59}H_{71}NO_6S$, $[M^*]^+$) 921.5002. *Signal found using ^{1}H-^{13}C HSQC NMR due to overlap with solvent residual signal.

(*E*)-3-(5-(4-(3,7-bis(2,4-bis(hexyloxy)phenyl)-10*H*-phenothiazin-10-yl)phenyl)furan-2-yl)-2-cyanoacrylic acid (10*H*-PTZ-Fu)

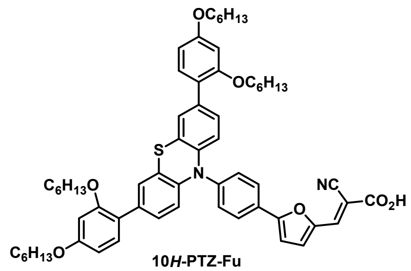

10*H*-PTZ-Fu

10*H*-PTZ-Fu was prepared as described for **TAA-Th** starting with the aldehyde **11** (54.0 mg, 58.6 μmol) and reacting for 1.5 h. Purification by silica-gel column chromatography (0-10% MeOH in CH_2Cl_2) gave **10*H*-PTZ-Fu** (49.5 mg, 50.0 μmol, 85%) as a red solid, mp. 173-178 °C. ^{1}H NMR (600 MHz, $CDCl_3$) δ: 11.11 (s, 1H), 8.03 (s, 1H), 7.95 (br.d, J = 6.3 Hz, 2H), 7.46-7.35 (m, 5H), 7.23-7.15 (m, 4H), 6.90 (s, 1H), 6.68 (d, J = 8.1 Hz, 2H), 6.53-6.49 (m, 4H), 4.00-3.90 (m, 8H), 1.83-1.68 (m, 8H), 1.52-1.23 (m, 24H), 0.92 (t, J = 7.0 Hz, 6H), 0.86 (t, J = 6.7 Hz, 6H); ^{13}C NMR (150 MHz, CDCl3) δ: 168.3, 160.7, 159.9 (2C), 157.1 (2C), 147.9, 144.9, 141.2 (2C), 140.2, 139.1, 134.5 (2C), 130.8 (2C), 128.6 (2C), 128.1 (2C), 127.6 (2C), 126.7, 125.8 (2C), 125.5, 125.2 (2C), 122.2 (2C), 119.6 (2C), 115.9, 109.3, 105.4 (2C), 100.5 (2C), 68.5 (2C), 68.3 (2C), 31.74 (2C), 31.64 (2C), 29.4 (2C), 29.2 (2C), 26.0 (2C), 25.9 (2C), 22.8 (4C), 14.2 (4C); IR (CH_2Cl_2, cm^{-1}): 2954, 2926, 2855, 1609, 1579, 1467, 1390, 1297, 1260, 1248, 1182, 1135, 1030, 797; HRMS (ASAP+, m/z): found 989.5113, calcd. for $C_{62}H_{72}N_2O_7S$, $[M^*]^+$ 989.5138.

1-(3-(2,4-bis(hexyloxy)phenyl)-7-bromo-10*H*-phenothiazin-10-yl)ethan-1-one (12)

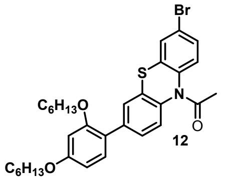

The compound **12** was prepared as described for compound **7** starting with the building block **5** (1.20 g, 3.01 mmol), except this time with a halved amount of 1-bromo-2,4-bis(hexyloxy)benzene (682 mg, 3.61 mmol) was used. The crude product was purified with column chromatography (*n*-pentane/EtOAc, 6:1, $R_f = 0.26$) to yield **12** as a colorless oil (800 mg, 1.34 mmol, 45%). ^{1}H NMR (600 MHz, DMSO-d_6) δ: 7.80 (d, J = 1.8 Hz, 1H), 7.65-7.54 (m, 4H), 7.46 (dd, J = 2.0 Hz, 8.3 Hz, 1H), 7.26 (d, J = 8.5 Hz, 1H), 6.63 (d, J = 2.4 Hz, 1H), 6.59 (dd, J = 2.0 Hz, 8.3 Hz, 1H), 4.03-3.94 (m, 4H), 2.16 (s, 3H), 1.71 (q, J = 7.7 Hz, 2H), 1.65-1.58 (m, 2H), 1.46-1.39 (m, 2H), 1.36-1.28 (m, 6H), 1.25-1.17 (m, 4H), 0.88 (t, J = 7.7 Hz, 3H), 0.79 (t, J = 7.7 Hz, 3H); ^{13}C NMR (150 MHz, DMSO-d_6) δ: 168.4, 159.9, 156.5, 138.0, 137.0, 136.5, 134.6, 130.9, 130.6, 130.0 (2C), 129.0, 128.2, 128.1, 126.7, 120.5, 119.1, 106.1, 100.1, 67.9, 67.6, 31.0, 30.9, 28.6, 28.5, 25.4, 25.2, 22.6, 22.07, 22.05, 13.9, 13.8; IR (CH_2Cl_2, cm^{-1}): 2927 (m), 2856 (m), 1684 (s), 1607 (s), 1480 (m), 1300 (m), 1269 (s), 1180 (m), 1012 (m); HRMS (ASAP+, m/z): found 596.1836, calcd. for $C_{32}H_{39}NO_3SBr$ $[M+H]^+$ 596.1834.

1-(3-(2,4-bis(hexyloxy)phenyl)-7-(furan-2-yl)-10*H*-phenothiazin-10-yl)ethan-1-one (13)

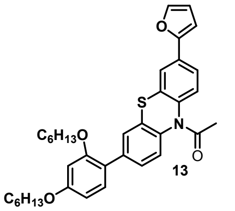

Compound **12** (475 mg, 0.80 mmol), $PdCl_2$(dppf) (30.7 mg, 42 µmol), and K_2CO_3 (463 mg, 3.35 mmol) were mixed with 2-furanylboronic acid (141 mg, 1.26 mmol), before the mixture was evacuated and a nitrogen atmosphere established. Degassed H_2O (7 mL) and 1,4-dioxane (7 mL) were added under a nitrogen atmosphere. This mixture was stirred at 80 °C for 1.5 h. After the mixture was cooled down to rt, H_2O (75 mL) was added, and the aqueous phase extracted with ethyl acetate (3 × 50 mL). The combined organic phase was washed with H_2O (2 × 50 mL), dried over Na_2SO_4 before the solvents were removed in vacuo. The crude product was purified with

column chromatography (CH_2Cl_2, $R_f = 0.16$) to yield **13** as a light-orange oil (298 mg, 0.51 mmol, 64%). ^{1}H NMR (400 MHz, DMSO-d_6) δ: 7.85 (d, J = 2.0 Hz, 1H), 7.78 (d, J = 2.0 Hz, 1H), 7.74-7.65 (m, 2H), 7.65-7.59 (m, 2H), 7.45 (dd, J = 2.0 Hz, 8.3 Hz, 1H), 7.26 (d, J = 8.6 Hz, 1H), 7.06 (d, J = 3.3 Hz, 1H), 6.65-6.56 (m, 3H), 4.03-3.94 (m, 4H), 2.18 (s, 3H), 1.76-1.67 (m, 2H), 1.66-1.57 (m, 2H), 1.47-1.38 (m, 2H), 1.37-1.27 (m, 6H), 1.26-1.19 (m, 4H), 0.88 (t, J = 6.5 Hz, 3H), 0.78 (t, J = 6.7 Hz, 3H); ^{13}C NMR (150 MHz, DMSO-d_6) δ: 168.5, 159.8, 156.5, 151.6, 143.5, 137.5, 136.8, 136.6, 130.9 (2C), 128.9, 128.1, 127.7 (2C), 126.7, 122.3, 122.1, 120.6, 112.3, 107.1, 106.1, 100.1, 97.2, 67.9, 67.6, 30.99, 30.95, 28.7, 28.5, 25.4, 25.2, 25.1, 22.07, 22.05, 13.9, 13.8; IR (CH_2Cl_2, cm^{-1}): 2928 (m), 2857 (m), 1682 (s), 1607 (s), 1464 (s), 1302 (s), 1274 (s), 1180 (m) 1101 (m); HRMS (ASAP+, m/z): found 584.2830, calcd. for $C_{36}H_{42}NO_4S$ $[M+H]^+$ 584.2835.

3-(2,4-bis(hexyloxy)phenyl)-7-(furan-2-yl)-10*H*-phenothiazine (14)

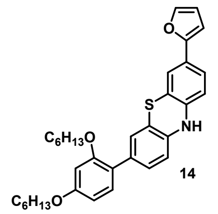

The compound **14** was prepared as described for compound **8** starting with the compound **13** (260 mg, 0.45 mmol). This produced **14** as a red resin (205 mg, 0.38 mmol, 85%). ^{1}H NMR (600 MHz, DMSO-d_6) δ: 8.76 (s, 1H), 7.65 (d, J = 1.3 Hz, 1H), 7.32 (dd, J = 2.1 Hz, 8.5 Hz, 1H), 7.23 (d, J = 2.1 Hz, 1H), 7.14 (d, J = 8.5 Hz, 1H), 7.07 (dd, J = 2.0 Hz, 8.2 Hz, 1H), 7.04 (d, J = 2.0 Hz, 1H), 6.74 (dd, J = 0.8 Hz, 3.4 Hz, 1H), 6.71 (d, J = 8.4 Hz, 1H), 6.68 (d, J = 8.2 Hz, 1H), 6.57 (d, J = 2.4 Hz, 1H), 6.55-6.51 (m, 2H), 4.00-3.91 (m, 4H), 1.70 (q, J = 8.0 Hz, 2H), 1.64 (q, J = 8.5 Hz, 2H), 1.46-1.35 (m, 4H), 1.35-1.21 (m, 8H), 0.91-0.83 (m, 6H); ^{13}C NMR (150 MHz, DMSO-d_6) δ: 159.1, 156.3, 152.5, 142.0, 141.1, 139.6, 131.8, 130.0, 128.2, 126.7, 124.3, 122.9, 121.4, 121.2, 116.9, 115.2, 114.5, 114.0, 111.9, 105.8, 104.0, 100.1, 67.7, 67.5, 31.00, 30.98, 28.7, 28.6, 25.4, 25.2, 22.12, 22.07, 13.91 (2C); IR (CH_2Cl_2, cm^{-1}): 3399 (w), 2954 (m), 2927 (m), 2857 (m), 1607 (m), 1488 (s), 1296 (m), 1271 (m), 1177 (m), 1010(m); HRMS (ASAP+, m/z): found 541.2644, calcd. for $C_{34}H_{39}NO_3S$ $[M+H]^+$ 541.2651.

4'-bromo-2,4-bis(hexyloxy)-1,1'-biphenyl (16)

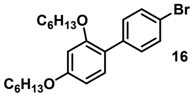

16

Compound 16 was prepared following a modified procedure reported by Seo et al.[1] 1-Bromo-4-iodobenzene **15** (200 mg, 0.71 mmol), $Pd(PPh_3)_4$ (40.9 mg, 35 µmol), and K_2CO_3 (391 mg, 2.83 mmol) were mixed with crude 2-(2,4-bis(hexyloxy)phenyl)-4,4,5,5-tetramethyl-1,3,2-dioxaborolane **6** (343 mg, 0.85 mmol), before the mixture was evacuated and a nitrogen atmosphere established. Degassed H_2O (7 mL) and 1,4-dioxane (7 mL) were added under a nitrogen atmosphere. This mixture was stirred at 80 °C for 1 h. After the mixture was cooled down to rt, H_2O (75 mL) was added and the aqueous phase extracted with ethyl acetate (3 × 50 mL). The combined organic phase was washed with H_2O (50 mL), dried over Na_2SO_4 before the solvents were removed in vacuo. The crude product was purified with column chromatography (*n*-pentane/CH_2Cl_2, 5:1, R_f = 0.16) to yield **16** as a yellow oil (189 mg, 0.44 mmol, 62%). ^{1}H NMR (400 MHz, DMSO-d_6) δ: 7.53 (d, J = 9.0 Hz, 2H), 7.39 (d, J = 9.0 Hz, 2H), 7.19, (d, J = 8.0 Hz, 1H), 6.62 (d, J = 2.5 Hz, 1H), 6.57 (dd, J = 2.5 Hz, 8.0 Hz, 1H), 4.02-3.92 (m, 4H), 1.71 (q, J = 7.7 Hz, 2H), 1.62 (q, J = 7.8 Hz, 2H), 1.47-1.37 (m, 2H), 1.37-1.27 (m, 6H) 1.27-1.18 (m, 4H), 0.88 (t, J = 7.8 Hz, 3H), 0.83 (t, J = 7.7 Hz, 3H); ^{13}C NMR (100 MHz, DMSO-d_6) δ: 159.7, 156.4, 137.3, 131.1 (2C), 130.68, 130.66 (2C), 121.0, 119.4, 106.0, 100.1, 67.9, 67.5, 31.0, 30.8, 28.7, 28.5, 25.20, 25.16, 22.1, 22.0, 13.9, 13.8.

10-(2',4'-bis(hexyloxy)-[1,1'-biphenyl]-4-yl)-3-(2,4-bis(hexyloxy)phenyl)-7-(furan-2-yl)-10*H*-phenothiazine (17)

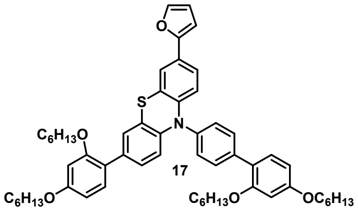

17

The compound **17** was prepared as described for compound **9** starting with the compound **14** (140 mg, 0.26 mmol) and compound **16** (135 mg, 0.31 mmol). The crude product was purified with column chromatography (*n*-pentane/CH_2Cl_2, 2:1, R_f = 0.68) to yield **17** as an orange wax (154 mg, 0.17 mmol, 65%). ^{1}H NMR (600 MHz, DMSO-d_6) δ: 7.77 (d, J = 8.6 Hz, 2H), 7.68 (d, J = 1.7 Hz,

1H), 7.44 (d, J = 8.6 Hz, 2H), 7.41 (d, J = 2.1 Hz, 1H), 7.36 (d, J = 8.6 Hz, 1H), 7.26 (dd, J = 2.2 Hz, 8.7 Hz, 1H), 7.22 (d, J = 2.1 Hz, 1H), 7.15 (d, J = 8.5 Hz, 1H), 7.03 (dd, J = 2.1 Hz, 8.6 Hz, 1H), 6.81 (d, J = 3.3 Hz, 1H), 6.68 (d, J = 2.3 Hz, 1H), 6.64 (dd, 2.3 Hz, 8.5 Hz, 1H), 6.59 (d, J = 2.2 Hz, 1H), 6.56-6.53 (m, 2H), 6.25 (d, J = 8.7 Hz, 1H), 6.21 (d, J = 8.5 Hz, 1H), 4.06-3.99 (m, 4H), 3.99-3.91 (m, 4H), 1.78-1.60 (m, 8H), 1.48-1.19 (m, 24H), 0.93-0.85 (m, 6H), 0.85-0.76 (m, 6H); ^{13}C NMR (150 MHz, DMSO-d_6) δ: 159.8, 159.3, 156.6, 156.4, 152.0, 142.7, 142.4, 141.5, 138.4, 138.0, 132.7, 131.6 (2C), 130.9, 130.1 (2C), 129.7, 129.1, 127.7, 127.0, 125.1, 122.3, 121.3, 120.8, 119.8, 117.9, 115.9, 115.5, 112.0, 106.1, 105.9, 104.8, 100.2, 100.1, 67.9, 67.8, 67.6, 67.5, 31.01, 30.99 (2C), 30.8, 28.69, 28.65, 28.6, 28.5, 25.4, 25.22 (2C), 25.19, 22.09 (2C), 22.07, 22.0, 13.92, 13.90, 13.85, 13.8; IR (CH_2Cl_2, cm^{-1}): 2926 (m), 2857 (m), 1607 (m), 1492 (s), 1296 (s), 1274 (s), 1178 (s), 1011 (m); HRMS (ASAP+, m/z): found 893.5048, calcd. for $C_{58}H_{71}NO_5S$ [M*]$^+$ 893.5053.

5-(10-(2',4'-bis(hexyloxy)-[1,1'-biphenyl]-4-yl)-7-(2,4-bis(hexyloxy)phenyl)-10*H*-phenothiazin-3-yl)furan-2-carbaldehyde (18)

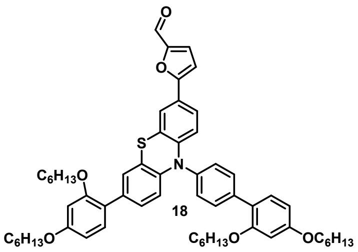

Compound **17** (130 mg, 0.15 mmol) was dissolved in anhydrous THF (4 mL) and cooled to -78 °C, a solution of *n*-BuLi in hexanes (1.4 M, 160 µL, 0.22 mmol) was added to this over 5 minutes. The reaction mixture was then stirred for 15 minutes before DMF (40 µL, 0.51 mmol) was added. After 15 more minutes of stirring, the reaction vessel was removed from the cooling mixture and left to reach r.t over 45 minutes. The reaction was quenched by addition of aqueous HCl (2 M, 4 mL), the aqueous phase was extracted with ethyl acetate (3 x 15 mL). The combined organic phase was washed with H_2O (15 mL), dried over Na_2SO_4 before the solvents were removed in vacuo. The crude product was purified with column chromatography (CH_2Cl_2, R_f = 0.58) to yield **18** as a red wax (46 mg, 50 µmol, 34%). ^{1}H NMR (600 MHz, CD_2Cl_2) δ: 9.55 (s, 1H), 7.81 (d, J = 8.8 Hz, 2H), 7.46 (d, J = 2.0 Hz, 1H), 7.41 (d, J = 8.8 Hz, 2H), 7.35, (d, J = 9.0 Hz, 1H), 7.30-7.26 (m, 2H), 7.22 (d, J = 2.0 Hz, 1H), 7.14 (d, J = 8.1 Hz, 1H), 7.02 (dd, J = 1.9 Hz, 8.7 Hz, 1H), 6.69 (d,

J = 4.1 Hz, 1H), 6.62-6.59 (m, 2H), 6.52-6.48 (m, 2H), 6.32 (d, J = 9.0 Hz, 1H), 6.27 (d, J = 9.0 Hz, 1H), 4.02 (app. t, J = 6.2 Hz, 4H), 3.96 (t, J = 6.7 Hz, 2H), 3.94 (t, J = 6.7 Hz, 2H), 1.84-1.69 (m, 8H), 1.52-1.41 (m, 8H), 1.40-1.28 (m, 16H), 0.96-0.84 (m, 12H); ^{13}C NMR (150 MHz, CD_2Cl_2) δ: 177.0, 160.8, 160.3, 159.2, 157.5, 157.3, 152.2, 145.9, 142.1, 139.6, 138.7, 133.9, 132.3 (3C), 131.5 (2C), 130.7, 130.5, 128.3, 127.8, 124.5, 123.5, 123.5, 122.4, 122.1, 121.0, 118.7, 116.2 (2C), 106.9, 106.0, 105.8, 100.64, 100.55, 68.9, 68.8, 68.64, 68.57, 32.04, 32.02, 31.99, 31.9, 29.71, 29.69, 29.6, 29.5, 26.3, 26.21, 26.15, 26.1, 23.1 (2C), 23.04, 23.02, 14.3, 14.24, 14.22 (2C); IR (CH_2Cl_2, cm^{-1}): 2953 (m), 2929 (m), 2858 (m), 1675 (m), 1609 (m), 1460 (s), 1298 (m), 1265 (m), 1182 (m); HRMS (ASAP+, m/z): found 921.4994, calcd. for $C_{59}H_{71}NO_6S$ [M*]$^+$ 921.5002.

(*E*)-3-(5-(10-(2',4'-bis(hexyloxy)-[1,1'-biphenyl]-4-yl)-7-(2,4-bis(hexyloxy)phenyl)-10*H*-phenothiazin-3-yl)furan-2-yl)-2-cyanoacrylic acid (3,7-PTZ-Fu)

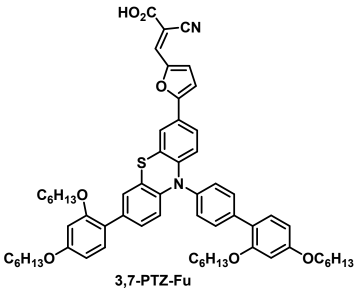

3,7-PTZ-Fu

3,7-PTZ-Fu was prepared as described for **TAA-Th** starting with the aldehyde **18** (45.0 mg, 48.8 *μ*mol) and reacting for 1.5 h. Purification by silica-gel column chromatography (0-10% MeOH in CH2Cl2) gave **3,7-PTZ-Fu** as a red solid (31.2 mg, 31.5 μmol, 65%), mp. 74.1-75.8 °C. ^{1}H NMR (600 MHz, acetone-d_6) δ: 8.01 (s, 1H), 7.86 (d, J = 8.3 Hz, 2H), 7.64 (d, J = 2.1 Hz, 1H), 7.51 (dd, J = 2.1 Hz, 8.9 Hz, 1H), 7.49 (d, J = 4.2 Hz, 1H), 7.46 (d, J = 8.3 Hz, 2H), 7.38 (d, J = 8.5 Hz, 1H), 7.26 (d, J = 2.1 Hz, 1H), 7.18 (d, J = 8.5 Hz, 1H), 7.10 (d, J = 3.6 Hz, 1H), 7.05 (dd, J = 2.1 Hz, 8.9 Hz, 1H), 6.69 (d, J = 2.5 Hz, 1H), 6.64 (dd, J = 2.5 Hz, 8.4 Hz, 1H), 6.60 (d, J = 2.5 Hz, 1H), 6.54 (dd, J = 2.5 Hz, 8.2 Hz, 1H), 6.33 (d, J = 8.7 Hz, 1H), 6.27 (d, J = 8.7 Hz, 1H), 4.09-4.04 (m, 4H), 4.02-3.97 (m, 4H), 1.84-1.68 (m, 8H), 1.54-1.43 (m, 8H), 1.41-1.24 (m, 16H), 0.95-0.81 (m, 12H); ^{13}C NMR (150 MHz, Acetone-d_6) δ: 164.3, 161.4, 160.9, 159.9, 158.1, 157.9, 148.5, 146.3, 142.3, 140.4, 139.1, 138.5, 134.7, 132.9 (2C), 132.0, 131.2, 130.9 (2C), 128.8, 128.2, 127.0, 125.3, 124.02 (2C), 122.8, 122.3, 121.4, 118.9, 117.0, 116.7, 116.7, 109.3, 106.9, 106.7, 101.1, 101.0, 97.1, 69.1, 69.0, 68.7, 68.6, 32.38, 32.36 (2C), 32.2, 29.9 (4C)*, 26.7, 26.6, 26.53,

26.51, 23.4, 23.33, 23.30 (2C), 14.43, 14.37, 14.34, 14.32; IR (CH_2Cl_2, cm^{-1}): 2953 (m), 2937 (m), 2869 (m), 2337 (w), 1686 (m), 1608 (m), 1493 (s), 1299 (m), 1263 (m), 1182 (m); HRMS (ASAP+, m/z): found 988.5042, calcd. for $C_{62}H_{72}N_2O_7S$ $[M^*]^+$ 988.5060. *Signal found using ^1H-^{13}C HSQC NMR due to overlap with solvent residual signal.

2,2'-((2-nitro-1,3-phenylene)bis(oxy))bis(bromobenzene) (21)

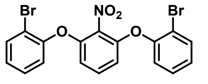

21

Sodium hydride (103 mg, 4.30 mmol) and 2-bromophenol **19** (0.3 mL, 2.60 mmol) was dissolved in DMSO (2 mL) before being heated to 50 °C. The reaction mixture was stirred for 15 minutes before 1,3-dichloro-2-nitrobenzene (100 mg, 0.52 mmol) was added, then the reaction was left stirring for 24 hours. Upon cooling to room temperature H_2O (10 mL) was added, and the aqueous phase was extracted using diethyl ether (3 x 25 mL). The combined organic phases were washed with water (3 x 25 mL), before being dried with brine and over Na_2SO_4. The crude product was concentrated in vacuo and purified by silica gel column chromatography (*n*-pentane/CH_2Cl_2, 10:1, R_f = 0.08) to give compound **21** as a yellow solid (136 mg, 0.29 mmol, 56%), mp. 128.4-129.6 °C. ^1H NMR (600 MHz, DMSO-d_6) δ: 7.81 (dd, J = 1.7 Hz, 8.1 Hz, 2H), 7.51 (td, J = 1.7 Hz, 8.2 Hz, 2H), 7.44 (t, J = 8.7 Hz, 1H), 7.35 (dd, J = 1.4 Hz, 8.2 Hz, 2H), 7.28 (td, J = 1.4 Hz, 7.9 Hz, 2H), 6.60 (d, J = 8.7 Hz, 2H); ^{13}C NMR (150 MHz, DMSO-d_6) δ: 150.7, 148.9, 134.1, 132.8, 132.6, 129.9, 127.7, 122.2, 114.5, 111.3; IR (CH_2Cl_2, cm^{-1}): 2895 (w), 1608 (m), 1464 (s), 1307 (m), 1244 (s), 1121 (s); HRMS (ASAP+, m/z): found 463.9138, calcd. for $C_{18}H_{12}NO_4Br_2$ $[M+H]^+$ 463.9133.

2,6-bis(2-bromophenoxy)aniline (22)

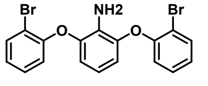

22

Compound **21** (100 mg, 0.22 mmol) was mixed with NH_4Cl (104 g, 1.94 mmol) and iron powder (36 mg, 0.65 mmol), before EtOH (2 mL) and water (0.5 mL) were added under nitrogen atmosphere. The reaction mixture was stirred at 78 °C for 4 h, cooled to rt, filtered through celite using ethyl acetate as eluent, and concentrated. This provided **22** as a colorless wax that was carried forward without further purification.

benzo[5,6][1,4]oxazino[2,3,4-*kl*]phenoxazine (23)

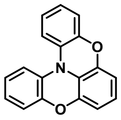

23

Compound **22** (94 mg, 0.22 mmol), Pd(OAc)2 (2.4 mg, 11 µmol), XPhos (10 mg, 22 µmol), and sodium tert-butoxide (42 mg, 0.43 mmol) was added to a Schlenk tube, and nitrogen atmosphere was established. Dry 1,4-dioxane (2 mL) was added under nitrogen and the reaction mixture was heated to 110 °C, the reaction was stirred for 18 hours. Upon cooling down to room temperature, an aqueous solution of NH_4Cl was added (saturated, 5 mL). The aqueous phase was extracted with ethyl acetate (3 x 25 mL), and the combined organic phase was dried with brine and over Na_2SO_4. The solvents were removed in vacuo, and the crude product was purified by silica gel column chromatography (*n*-pentane/CH_2Cl_2, 1:1, R_f = 0.66) to give compound **23** as a white solid (26 mg, 0.11 mmol, 50% over two steps from **21**), mp. 163.7-164.8 °C (lit. 165 °C).[2] ^{1}H NMR (600 MHz, DMSO-d_6) δ: 7.37 (d, J = 8.2 Hz, 2H), 7.08-6.95 (m, 6H), 6.87 (t, J = 8.3 Hz, 1H), 6.64 (d, J = 8.3 Hz, 2H); ^{13}C NMR (150 MHz, DMSO-d_6) δ: 146.0, 144.6, 128.3, 124.3, 124.2, 123.9, 120.2, 117.3, 114.8, 111.2; IR (CH_2Cl_2, cm^{-1}): 3425 (w), 2922 (w), 2850 (w), 1618 (m), 1468 (s), 1306 (m), 1281 (s), 1112 (m), 738 (s); HRMS (ASAP+, m/z): found 274.0867, calcd. for $C_{18}H_{12}NO_2$ $[M+H]^+$ 274.0868. ^{1}H NMR spectrum is accordance with previously reported data.[2]

7-bromobenzo[5,6][1,4]oxazino[2,3,4-kl]phenoxazine (24)

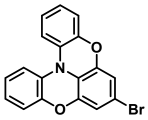

24

The synthesis of 7-bromobenzo[5,6][1,4]oxazino[2,3,4-kl]phenoxazine, **24**, was attempted through the following two procedures [3,4] with slight modifications. In our hands the resulting product mixture proved impossible to purify by column chromatography or recrystallization.

NBS-procedure: A solution of compound **23** (20 mg, 0.073 mmol) in chloroform (4 mL) is mixed with N-bromosuccinimide (11.7 g, 0.066 mmol) at 0 °C under exclusion of light, and the mixture was stirred at this temperature for 2 h. The reaction was quenched by addition of sodium sulfite solution (5w%, 10 mL), and the mixture was stirred at room temperature for a further 30 min. After phase separation, the organic phase was washed with water (20 mL), and the aqueous phase was extracted with dichloromethane (3 x 20 mL).

The combined organic phases were dried over sodium sulfate and concentrated in vacuum. The residue was dissolved in ethyl acetate and filtered over silica gel. The crude product was subsequently recrystallized from hexane.

Br_2-Procedure:

A stirred solution of compound **23** (50 mg, 0.18 mmol) in acetic acid (2 mL) was flushed with nitrogen then a solution of bromine (1 M, 0.20 mL, 1 mmol) in acetic acid was added dropwise, and stirring was continued overnight. The reaction was quenched by addition of sodium sulfite (5w%, 20 mL) solution, and the mixture was stirred at room temperature for a further 30 min. After phase separation, the organic phase was washed with water (20 mL), and the aqueous phase was extracted with dichloromethane (3 x 20 mL). The combined organic phases were dried over sodium sulfate and concentrated in vacuum. The crude product was subjected to silica gel column chromatography (*n*-pentane/CH_2Cl_2, 1:3, $R_f = 0.30$).

4-bromo-2',4'-bis(hexyloxy)-[1,1'-biphenyl]-3-ol (26)

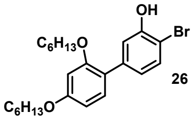

26

The compound **26** was prepared as described for compound **16** starting with the building block **6** (1.40 g, 3.45 mmol), except this time with it was coupled with 2-bromo-5-iodophenol **25** (860 mg, 2.88 mmol) in a 3 hour reaction. The crude product was purified with column chromatography (*n*-pentane/EtOAc, 10:1, $R_f = 0.37$) to yield **26** as a colorless oil (663 mg, 1.58 mmol, 51%). ^{1}H NMR (400 MHz, DMSO-d_6) δ: 10.09 (s, 1H), 7.41 (d, *J* = 8.9 Hz, 1H), 7.14 (d, *J* = 8.2 Hz, 1H), 7.06 (d, *J* = 2.0 Hz, 1H), 6.80 (dd, *J* = 2.0 Hz, 8.9 Hz, 1H), 6.60 (d, *J* = 2.5 Hz, 1H), 6.56 (dd, *J* = 2.5 Hz, 8.2Hz, 1H), 3.98 (t, *J* = 6.6 Hz, 2H), 3.94 (t, *J* = 6.1 Hz, 2H), 1.71 (q, *J* = 6.6 Hz, 2H), 1.63 (q, *J* = 6.1 Hz, 2H), 1.45-1.38 (m, 2H), 1.36-1.28 (m, 6H), 1.28-1.20 (m, 4H), 0.88 (t, *J* = 6.6 Hz, 3H), 0.83 (t, *J* = 6.1 Hz, 3H); ^{13}C NMR (100 MHz, DMSO-d_6) δ:159.6, 156.4, 153.5, 138.7, 131.9, 130.5, 121.5, 121.4, 117.1, 107.1, 105.9, 100.2, 68.0, 67.5, 31.0, 30.9, 28.7, 28.5, 25.2, 25.1, 22.1, 22.0, 13.9, 13.8; IR (CH_2Cl_2, cm^{-1}): 2928, 2857, 1682, 1608, 1499, 1301, 1277, 1181; HRMS (ASAP+, m/z): found 449.1657, calcd. for $C_{24}H_{34}BrO_3$ $[M+H]^+$ 449.1691.

2-(3,5-difluoro-4-nitrophenyl)thiophene (28)

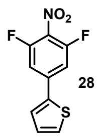

The compound **28** was prepared as described for compound **2** starting with the building block 5-bromo-1,3-difluoro-2-nitrobenzene **27** (250 mg, 1.05 mmol) and thiophen-2-ylboronic acid (202 mg, 1.58 mmol) The crude product was purified with column chromatography (*n*-pentane/toluene, 3:1, R_f = 0.28) to yield **28** as a green solid (188 mg, 0.78 mmol, 74%), mp. 96.3-97.1 °C. ^{1}H NMR (600 MHz, DMSO-*d*$_6$) δ: 7.91 (d, *J* = 4.0 Hz, 1H), 7.86-7.80 (m, 3H), 7.25 (dd, *J* = 4.0 Hz, 5.0 Hz, 1H); ^{13}C NMR (150 MHz, DMSO-*d*$_6$) δ: 154.8 (dd, *J* = 2.2 Hz, 258.7 Hz), 140.1 (t, *J* = 11.0 Hz), 138.9 (t, *J* = 2.6 Hz), 130.3 (s), 129.2 (s), 128.5 (s), 126.6 (t, *J* = 14.2 Hz), 109.7 (dd, *J* = 3.3 Hz, 21.9 Hz); IR (CH_2Cl_2, cm^{-1}): 3110 (w), 1610 (m), 1478 (m), 1369 (m), 1233 (m), 1083 (m); HRMS (ASAP+, m/z): found 241.0013, calcd. for $C_{10}H_5NO_2F_2S$ $[M+H]^+$ 241.0009.

2-(3,5-bis((4-bromo-2',4'-bis(hexyloxy)-[1,1'-biphenyl]-3-yl)oxy)-4-nitrophenyl)thiophene (29)

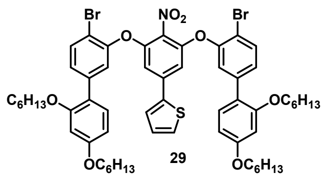

Compound **26** (407 mg, 0.91 mmol) and compound **28** (100 mg, 0.41 mmol) were dissolved in DMF (3 mL). K_2CO_3 (378 mg, 2.74 mmol) was added and the mixture was stirred at 100 °C for 4 h. After addition of 1 M HCl aq. (5 mL), the products were extracted with CH_2Cl_2 (3 × 25 mL). The organic phase was dried over Na_2SO_4, filtered off, and concentrated under reduced pressure. The obtained crude product was purified by silica gel column chromatography (CH_2Cl_2, R_f = 0.59) to give compound **29** as a yellow wax (269 mg, 0.24 mmol, 59%). ^{1}H NMR (400 MHz, DMSO-*d*$_6$) δ: 7.80 (d, *J* = 8.6 Hz, 2H), 7.61 (dd, *J* = 1.3 Hz, 5.0 Hz, 1H), 7.48 (d, *J* = 2.0 Hz, 2H), 7.46 (dd, *J* = 1.2 Hz, 3.8 Hz, 1H), 7.34 (dd, *J* = 2.3 Hz, 8.6 Hz, 2H), 7.30 (app. d, *J* = 9.4 Hz, 2H), 7.08 (dd, *J* = 3.8 Hz, 5.0 Hz, 1H), 6.91 (s, 2H), 6.61-6.57 (m, 4H), 3.99 (t, *J* = 6.6 Hz, 4H), 3.86 (t, *J* = 6.6 Hz, 4H), 1.70 (q, *J* = 7.7 Hz, 4H), 1.46-1.37 (m, 8H), 1.34-1.27 (m, 8H), 1.15-1.09 (m, 4H), 1.07-0.95 (m, 8H), 0.87 (t, *J* = 7.0 Hz, 6H), 0.67 (t, *J* = 7.7 Hz, 6H); ^{13}C NMR (150 MHz, DMSO-

d_6) δ: 160.1 (2C), 156.4 (2C), 150.4 (2C), 149.5 (2C), 139.7 (3C), 138.0, 133.5 (2C), 132.3, 130.8 (2C), 129.0, 128.6 (2C), 127.6, 126.9, 121.7 (2C), 119.6 (2C), 111.4 (2C), 108.7 (2C), 106.2 (2C), 100.0 (2C), 67.9 (2C), 67.6 (2C), 31.0 (2C), 30.7 (2C), 28.6 (2C), 28.4 (2C), 25.2 (2C), 25.0 (2C), 22.1 (2C), 21.9 (2C), 13.9 (2C), 13.7 (2C); IR (CH_2Cl_2, cm^{-1}): 2928 (m), 2857 (m), 1604 (m), 1509 (m), 1299 (m), 1244 (s), 1180 (s), 700 (m); HRMS (ASAP+, m/z): found 1097.3098, calcd. for $C_{58}H_{69}NO_8SBr_2$ $[M^*]^+$ 1097.3111.

2,6-bis((4-bromo-2',4'-bis(hexyloxy)-[1,1'-biphenyl]-3-yl)oxy)-4-(thiophen-2-yl)aniline (30)

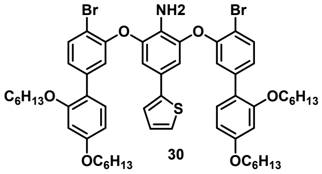

A solution of **29** (250 mg, 0.23 mmol) in acetone (20 mL) was treated with saturated aqueous NH_4Cl (5 mL), followed by Zn^0 (297 mg, 4.5 mmol). The reaction mixture was stirred at room temperature for 3 h before the reaction mixture was filtered through a plug of Celite (EtOAc). Removal of solvents in vacuo provided **30** as a colorless wax that was carried forward without further purification.

3,11-bis(2,4-bis(hexyloxy)phenyl)-7-(thiophen-2-yl)benzo[5,6][1,4]oxazino[2,3,4-*kl*]phenoxazine (31)

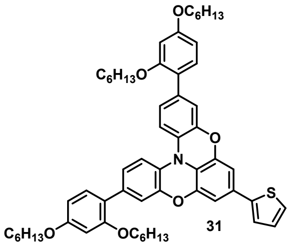

The compound **31** was prepared as described for compound **23** starting with the crude **30** (243 mg, 0.23 mmol). The crude **31** was purified with column chromatography (*n*-pentane/CH_2Cl_2, 10:1, R_f = 0.43) to yield **31** as a slight green oil (80 mg, 0.09 mmol, 39% over two steps from **29**). ^{1}H NMR (600 MHz, CD_2Cl_2) δ:7.38 (d, J = 7.8 Hz, 2H), 7.27-7.21 (m, 4H), 7.17-7.12 (m, 4H), 7.05 (dd, J = 3.6 Hz, 5.0 Hz, 1H), 6.80 (s, 2H), 6.57-6.51 (m, 4H), 4.03-3.95 (m, 8H), 1.84-1.74 (m, 8H), 1.53-1.44 (m, 8H), 1.42-1.32 (m, 16H), 0.98-0.88 (m, 12H); ^{13}C NMR (150 MHz, CD_2Cl_2) δ: 160.4 (2C), 157.4 (2C), 146.4 (2C), 145.8 (2C), 143.5, 134.5 (2C), 130.9 (2C), 130.7, 128.4, 127.3 (2C), 124.9 (2C), 124.8, 123.2, 122.2 (2C), 120.6, 118.7 (2C), 114.5 (2C), 108.8 (2C), 105.9 (2C), 100.6

(2C), 68.9 (2C), 68.6 (2C), 32.1 (2C), 32.0 (2C), 29.7 (2C), 29.6 (2C), 26.3 (2C), 26.2 (2C), 23.1 (4C), 14.28 (2C), 14.26 (2C); IR (CH_2Cl_2, cm^{-1}): 2928, 2857, 1682, 1608, 1499, 1301, 1277, 1181; HRMS (ASAP+, m/z): found 907.3835, calcd. for $C_{58}H_{69}NO_6S$ $[M*]^+$ 907.3846.

5-(3,11-bis(2,4-bis(hexyloxy)phenyl)benzo[5,6][1,4]oxazino[2,3,4-*kl*]phenoxazin-7-yl)thiophene-2-carbaldehyde (32)

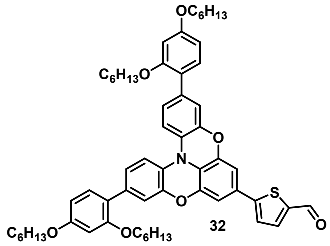

Compound **31** (80 mg, 0.09 mmol) was dissolved in anhydrous THF (2 mL) and cooled to -78 °C, a solution of lithium diisopropylamide in THF (2.0 M, 66 μL, 0.13 mmol) was added to this over 5 minutes. The reaction mixture was then stirred for 15 minutes before DMF (20 μL, 0.26 mmol) was added. After 15 more minutes of stirring, the reaction vessel was removed from the cooling bath and left to reach r.t over 45 minutes. The reaction was quenched by addition of aqueous HCl (2 M, 3 mL), the aqueous phase was extracted with ethyl acetate (3 x 15 mL). The combined organic phase was washed with H_2O (15 mL), dried over Na_2SO_4 before the solvents were removed in vacuo. The crude product was purified with column chromatography (CH_2Cl_2, R_f = 0.44) to yield **32** as a red wax (40 mg, 43 μmol, 48%). ^1H NMR (600 MHz, CD_2Cl_2) δ: 9.83 (s, 1H), 7.69 (d, J = 4.2 Hz, 1H), 7.37 (d, J = 8.9 Hz, 2H), 7.30 (d, J = 4.2 Hz, 1H), 7.22 (d, J = 7.4 Hz, 2H), 7.17-7.12 (m, 4H), 6.84 (s, 2H), 6.56-6.51 (m, 4H), 4.01-3.95 (m, 8H), 1.83-1.73 (m, 8H), 1.52-1.44 (m, 8H), 1.41-1.30 (m, 16H), 0.96-0.87 (m, 12H); ^{13}C NMR (150 MHz, CD_2Cl_2) δ: 182.9, 160.4 (2C), 157.4 (2C), 153.0, 146.3 (2C), 145.9 (2C), 142.3 (2C), 137.8, 134.9 (2C), 130.9 (2C), 129.1, 126.9, 125.0 (2C), 124.1, 122.7, 122.1 (2C), 118.7 (2C), 114.6 (2C), 109.5 (2C), 105.9 (2C), 100.6 (2C), 68.9 (2C), 68.6 (2C), 32.1 (2C), 32.0 (2C), 29.7 (2C), 29.6 (2C), 26.3 (2C), 26.2 (2C), 23.07 (2C), 23.06 (2C), 14.3 (2C), 14.2 (2C); IR (CH_2Cl_2, cm^{-1}): 2926 (m), 2857 (m), 1667 (s), 1608 (s), 1491 (s), 1299 (s), 1282 (s), 1179 (m), 797 (m); HRMS (ASAP+, m/z): found 935.4779, calcd. for $C_{59}H_{69}NO_7S$ $[M*]^+$ 935.4795.

(*E*)-3-(5-(3,11-bis(2,4-bis(hexyloxy)phenyl)benzo[5,6][1,4]oxazino[2,3,4-*kl*]phenoxazin-7-yl)thiophen-2-yl)-2-cyanoacrylic acid (POZPOZ-Th)

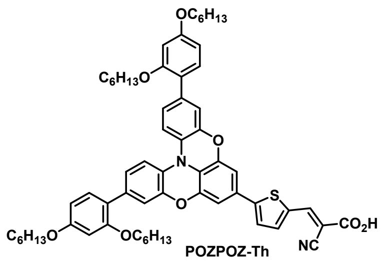

POZPOZ-Th was prepared as described for **TAA-Th** starting with the aldehyde **32** (40.0 mg, 42.7 *μ*mol) and reacting for 2.5 h. Purification by silica-gel column chromatography (0-10% MeOH in CH2Cl2) gave **POZPOZ-Th** as a dark purple solid (36.2 mg, 36.0 μmol, 84%); mp. 82.4-84.1 °C. ^{1}H NMR (600 MHz, DMSO-d_6) δ: 8.35 (s, 1H), 7.86 (d, J = 4.2 Hz, 1H), 7.60 (d, J = 4.2 Hz, 1H), 7.23 (d, J = 8.6 Hz, 2H), 7.12 (d, J = 8.4 Hz, 2H), 7.07-7.00 (m, 4H), 6.89 (s, 2H), 6.55 (s, 2H), 6.47 (d, J = 8.4 Hz, 2H), 3.98-3.88 (m, 8H), 1.74-1.61 (m, 8H), 1.43-1.35 (m, 8H), 1.34-1.23 (m, 16H), 0.88 (t, J = 6.9 Hz, 6H), 0.83 (t, J = 6.7 Hz, 6H); ^{13}C NMR (150 MHz, DMSO-d_6) δ: 163.6, 159.4 (2C), 156.4 (2C), 150.6, 145.6, 144.9 (2C), 144.7 (2C), 140.5, 134.3, 134.0 (2C), 130.2 (2C), 128.2, 125.5 (2C), 124.8, 124.3, 121.2 (2C), 120.6 (3C), 117.7 (2C), 116.7, 114.1 (2C), 108.6 (2C), 105.7 (2C), 100.0 (2C), 67.8 (2C), 67.5(2C), 31.04 (2C), 30.98 (2C), 28.7 (2C), 28.6 (2C), 25.4 (2C), 25.2 (2C), 22.1 (4C), 13.9 (2C), 13.8 (2C); IR (CH_2Cl_2, cm^{-1}): 3064 (m), 3041(m), 2928 (m), 2859 (m), 2220 (w), 1692 (m), 1609 (m), 1494 (s), 1300 (m), 1282 (m), 1182 (m); HRMS (ASAP+, m/z): found 1002.4837, calcd. for $C_{62}H_{70}N_2O_8S$ [M*]$^+$ 1002.4853.

References

1. Seo, K. D.; Choi, I. T.; Kim, H. K., D–π–A organic dyes with various bulky amine-typed donor moieties for dye-sensitized solar cells employing the cobalt electrolyte. *Org. Electron.* **2015,** *25*, 1-5. DOI:10.1016/j.orgel.2015.06.011

2. Kuratsu, M.; Kozaki, M.; Okada, K., Synthesis, Structure, and Electron-Donating Ability of 2,2′:6′,2″-Dioxatriphenylamine and Its Sulfur Analogue. *Chem. Lett.* **2004,** *33* (9), 1174-1175. DOI:10.1246/cl.2004.1174

3. Brocke, C.; Plumm, C.; Parham, H.A.; Fortte, R., Heteroaromatic compounds and their preparation and use in electronic devices. WO2011107186A2. **2011**.

4. Parham, A.; Kroeber, J.; Joosten, D.; Ludemann, A.; Grossmann, T., Bridged triarylamine derivatives, their preparation, use in electronic devices, and the devices. WO2018095839A1. **2018**.

NMR

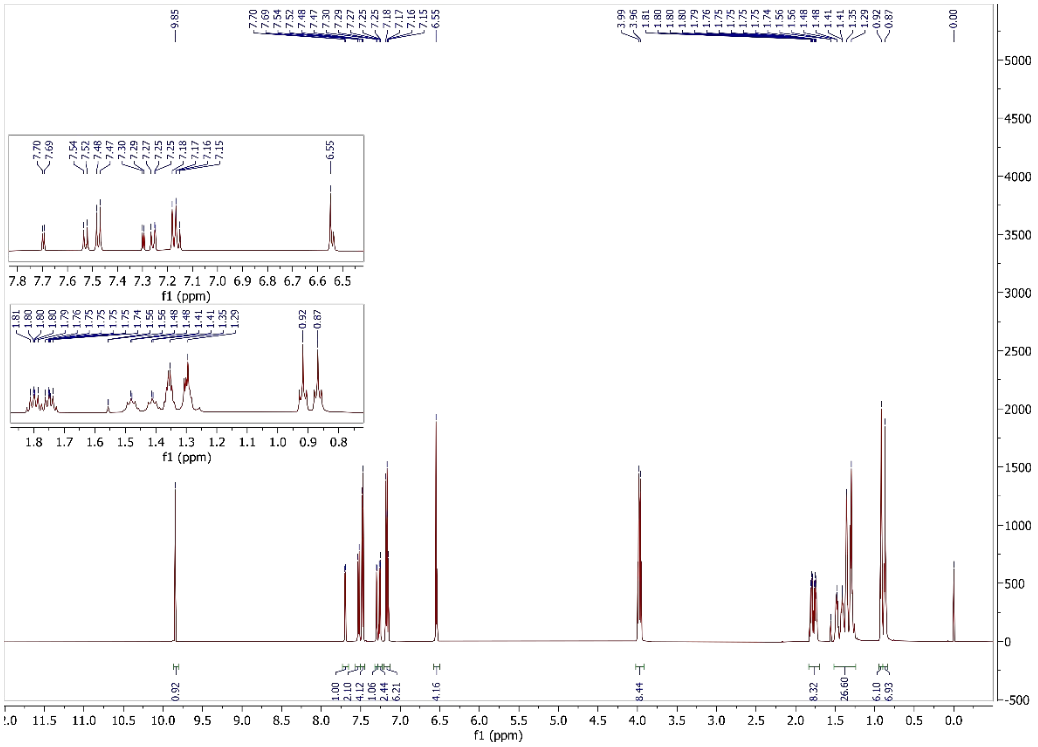

Figure S5 ^{1}H NMR (600 MHz, $CDCl_3$) of compound **2**.

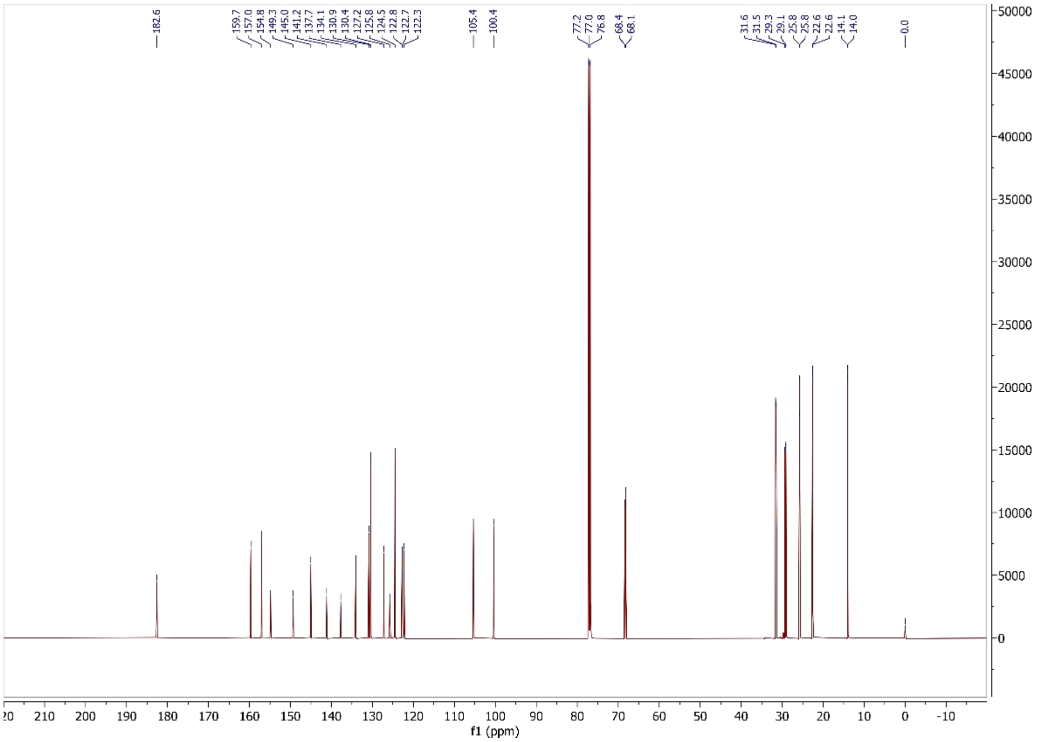

Figure S6 ^{13}C NMR (150 MHz, $CDCl_3$) of compound **2**.

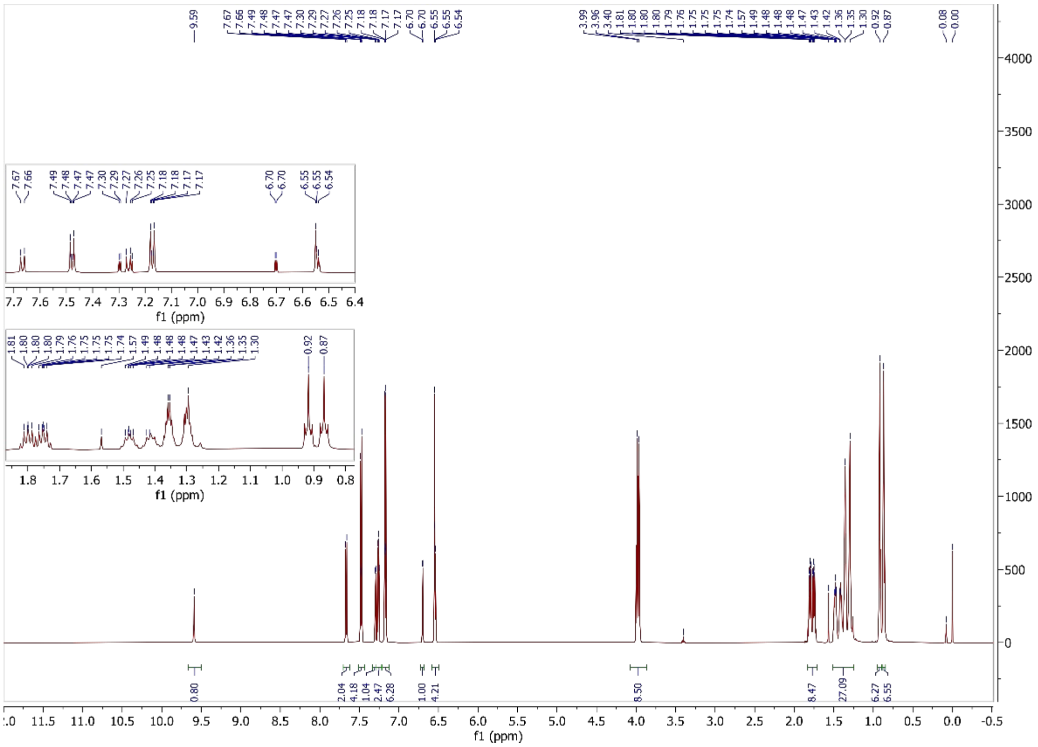

Figure S7 ^{1}H NMR (600 MHz, $CDCl_3$) of compound **3**.

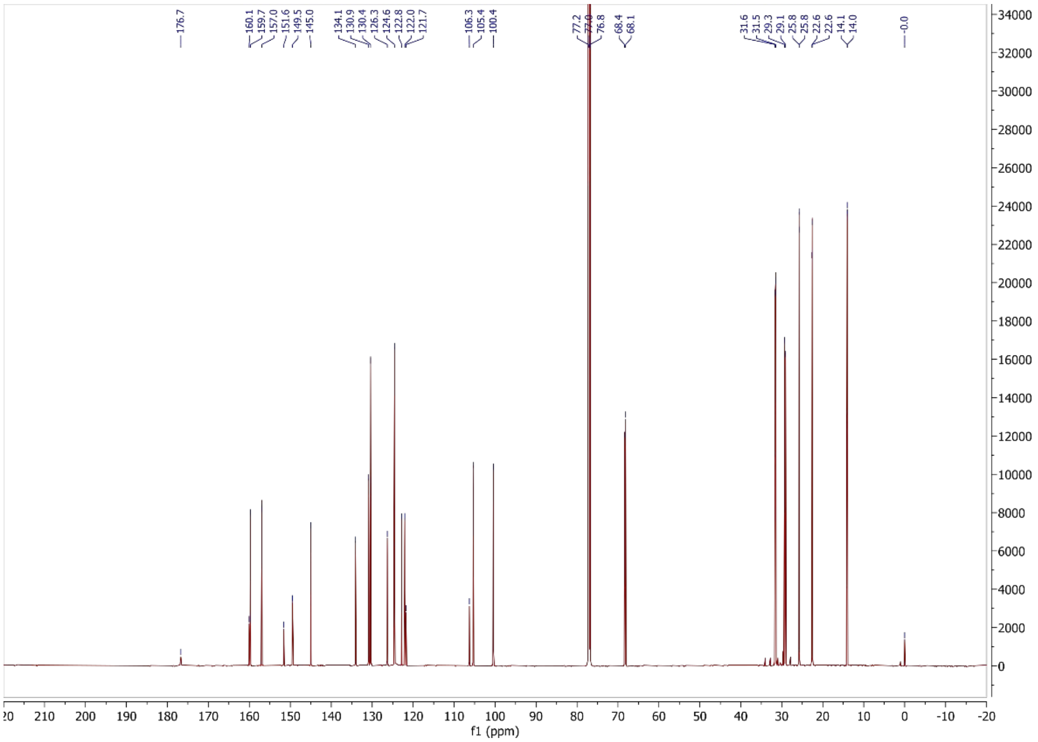

Figure S8 ^{13}C NMR (150 MHz, $CDCl_3$) of compound **3**.

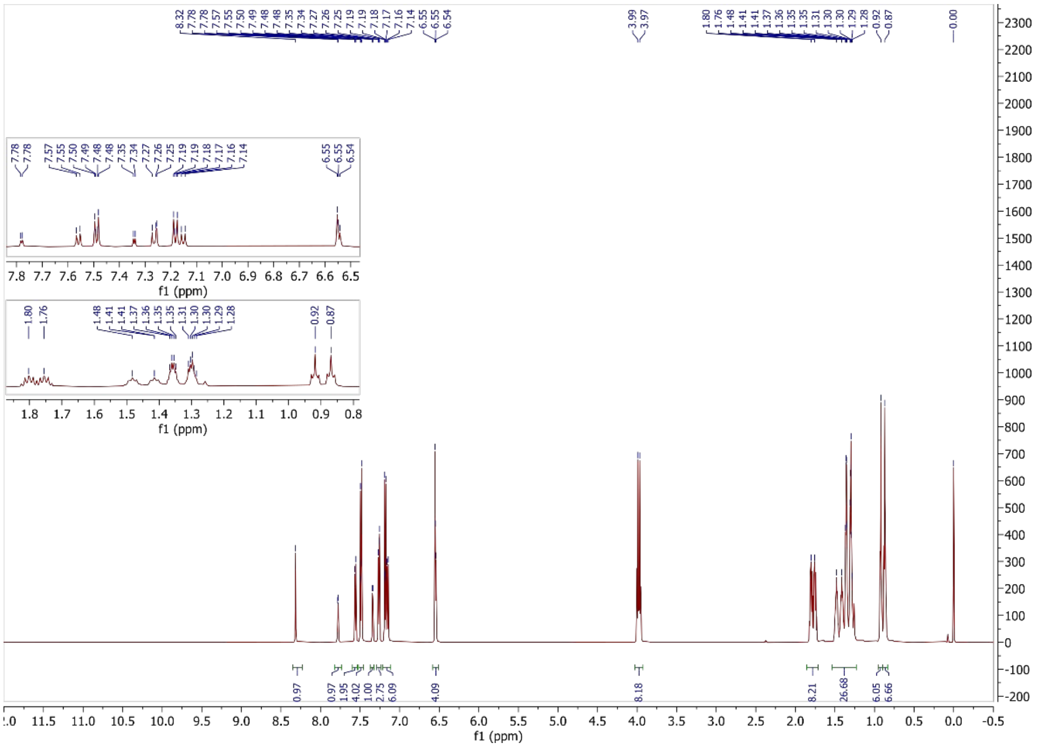

Figure S9 ^{1}H NMR (600 MHz, $CDCl_3$) of dye **TAA-Th**.

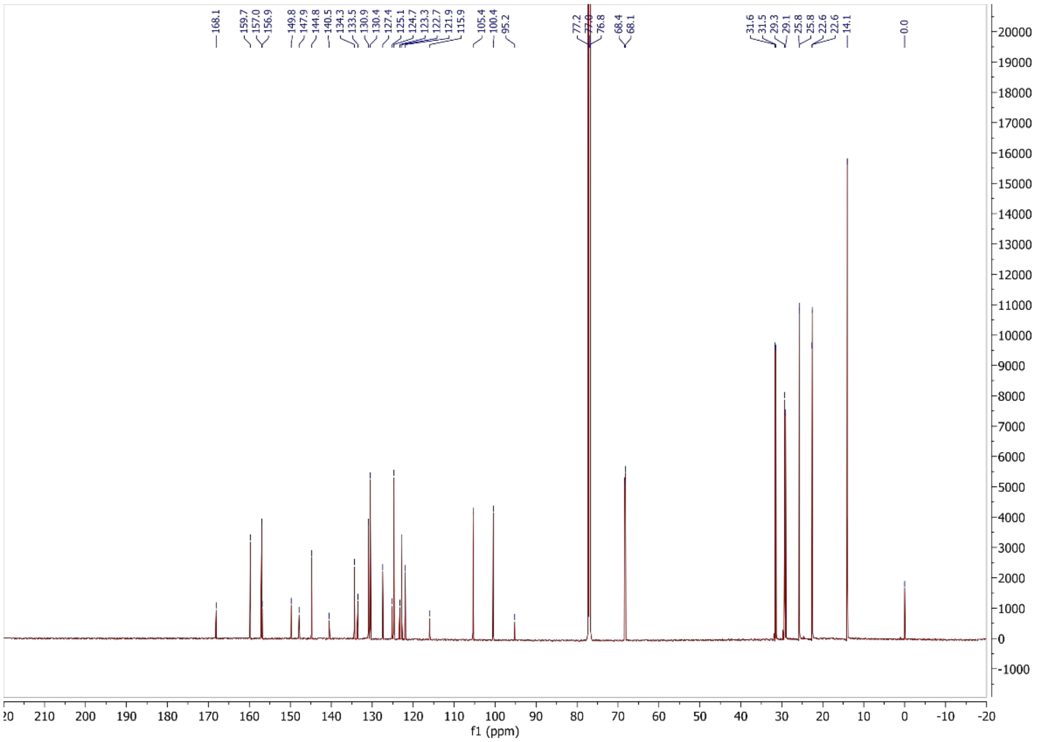

Figure S10 ^{13}C NMR (150 MHz, $CDCl_3$) of dye **TAA-Th**.

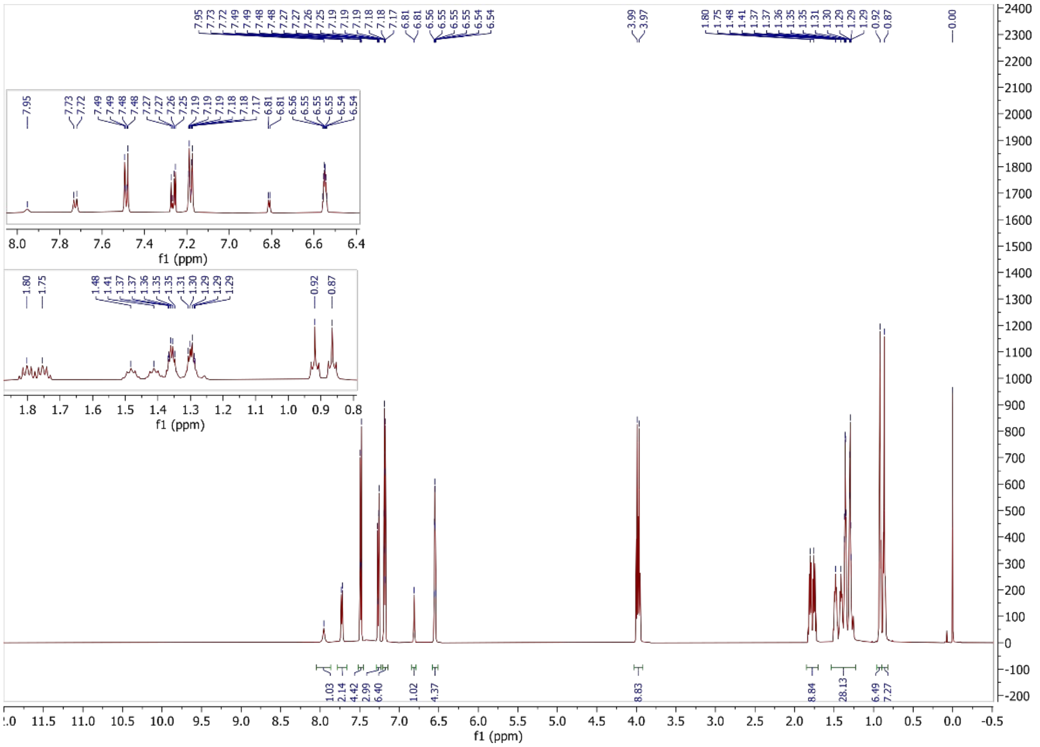

Figure S11 ^{1}H NMR (600 MHz, $CDCl_3$) of dye **TAA-Fu**.

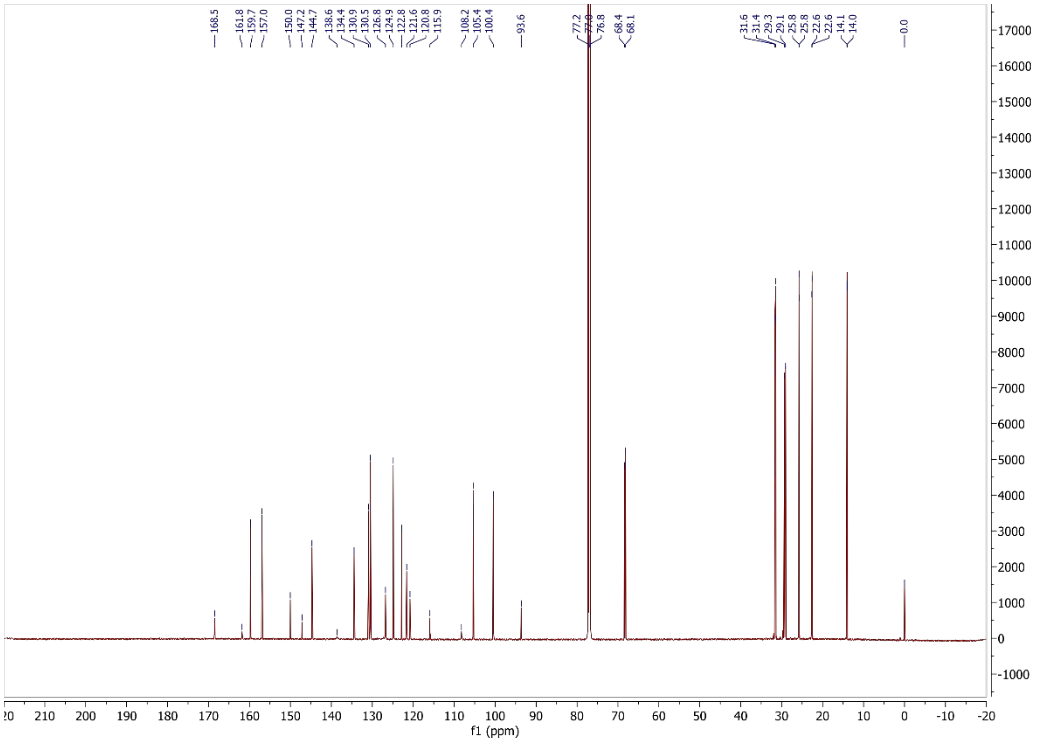

Figure S12 ^{13}C NMR (150 MHz, $CDCl_3$) of dye **TAA-Fu**.

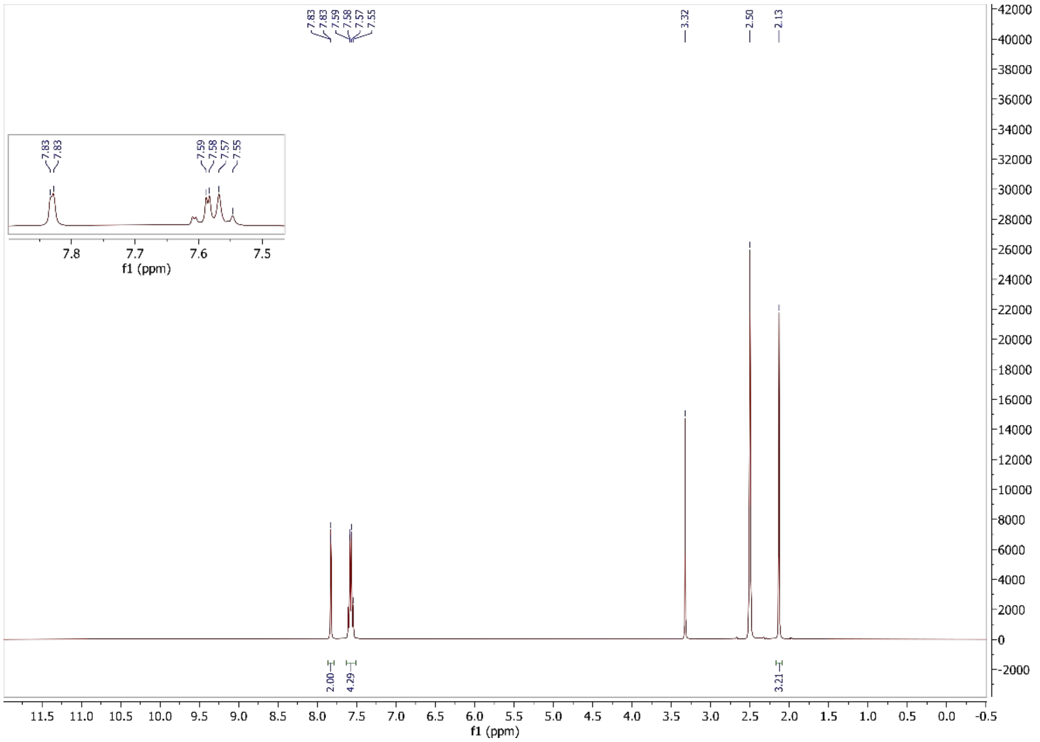

Figure S13 ^{1}H NMR (600 MHz, DMSO-d_6) of compound **5**.

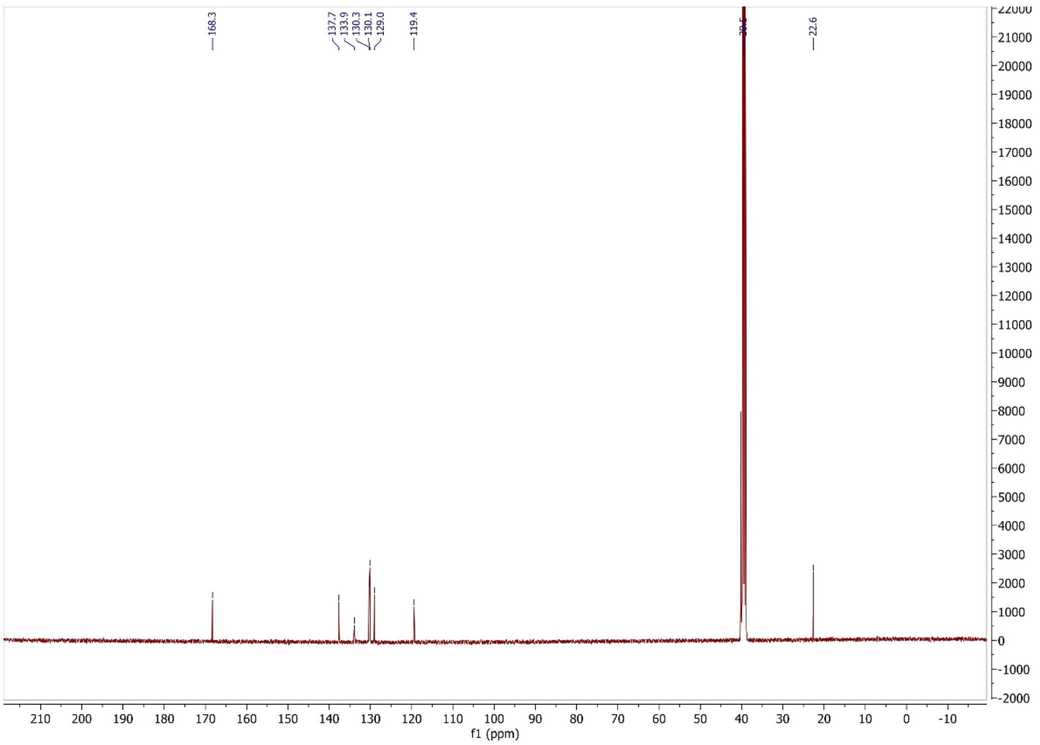

Figure S14 ^{13}C NMR (150 MHz, DMSO-d_6) of compound **5**.

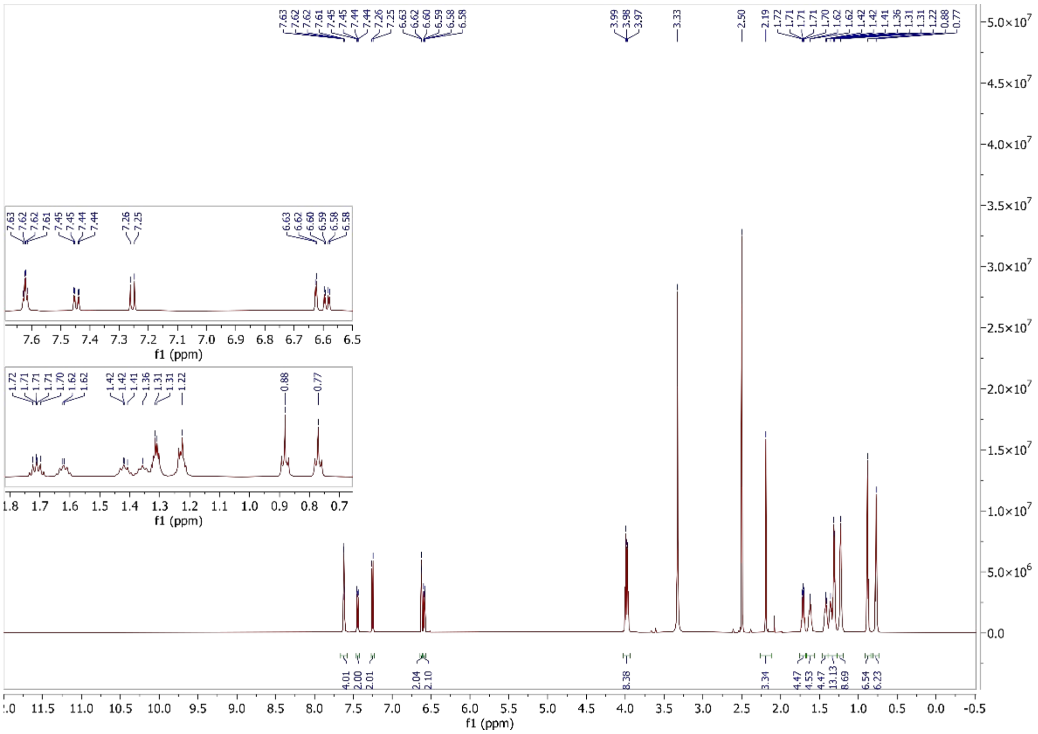

Figure S15 ^{1}H NMR (600 MHz, DMSO-d_6) of compound **7**.

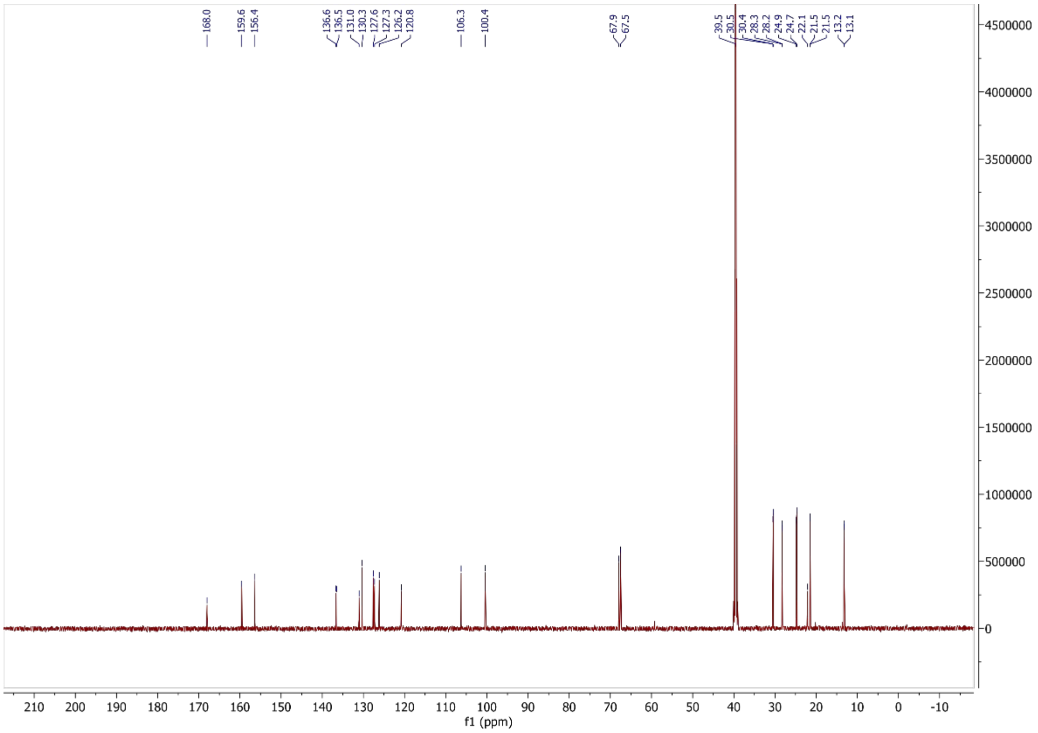

Figure S16 ^{13}C NMR (150 MHz, DMSO-d_6) of compound **7**.

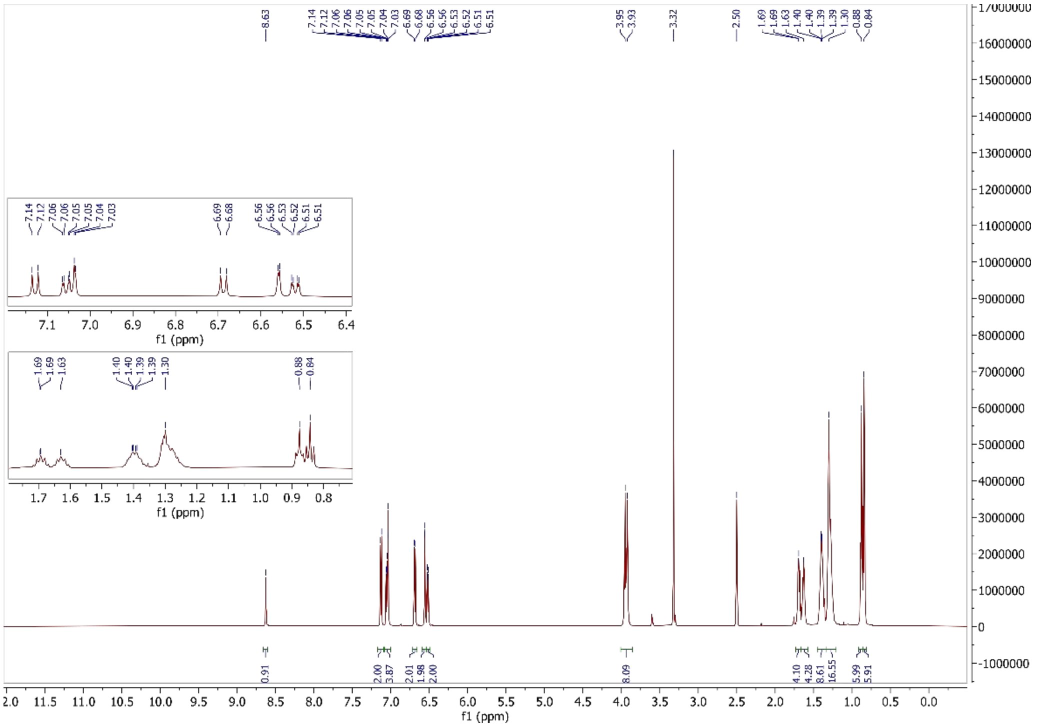

Figure S17 ^{1}H NMR (600 MHz, DMSO-d_6) of compound **8**.

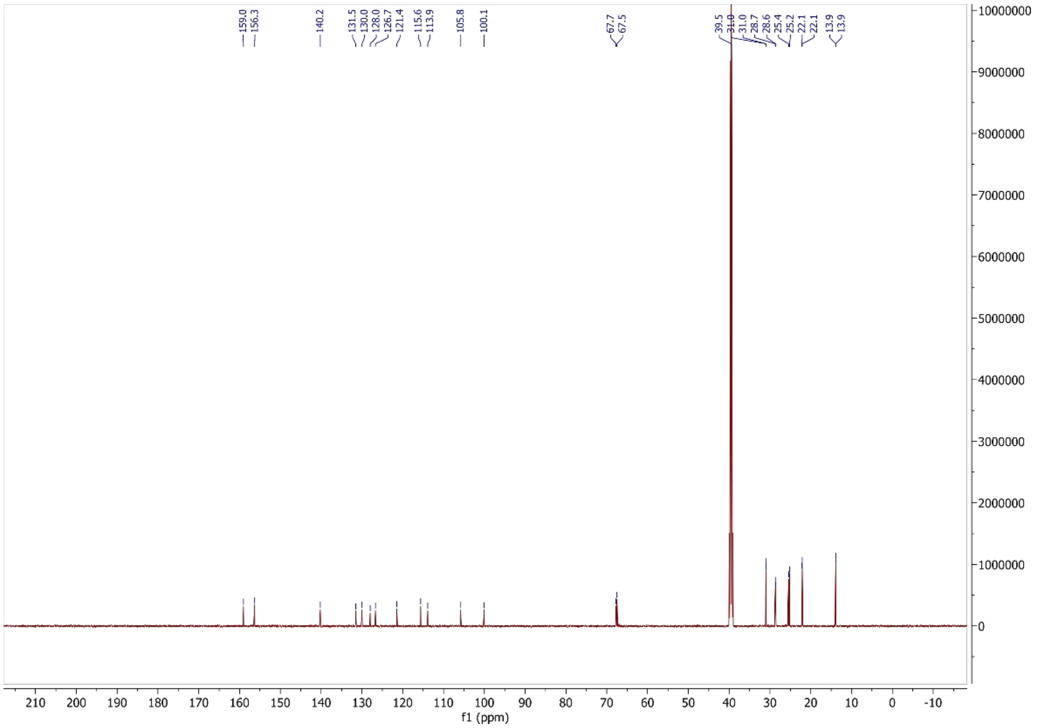

Figure S18 ^{13}C NMR (150 MHz, DMSO-d_6) of compound **8**.

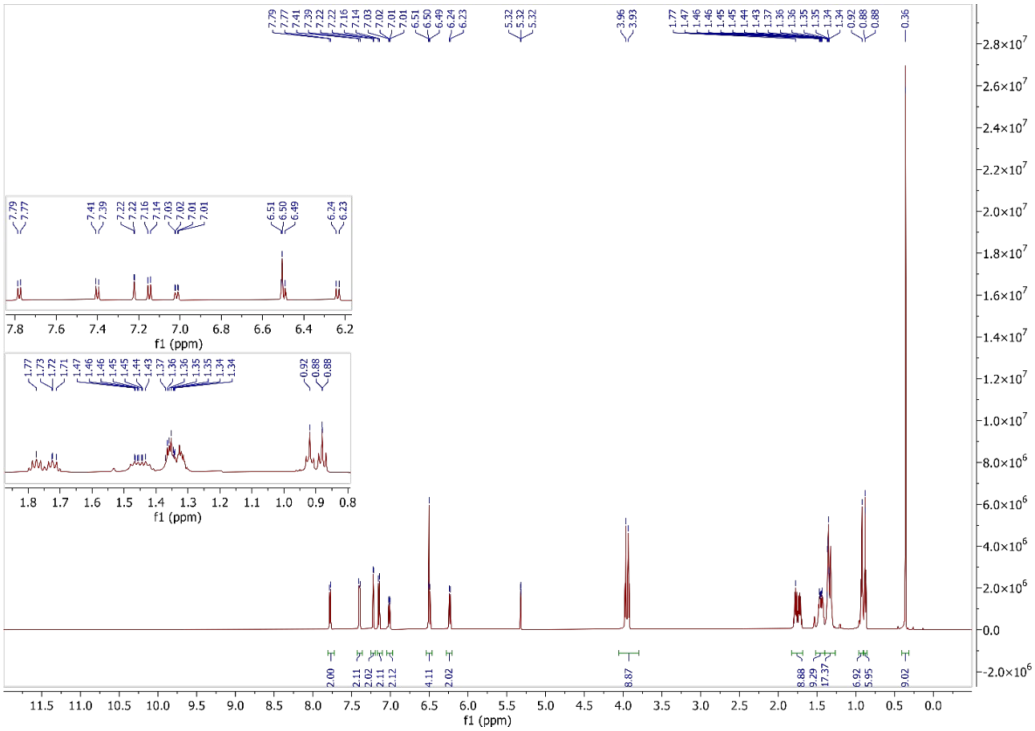

Figure S19 ^{1}H NMR (600 MHz, CD_2Cl_2) of compound **9**.

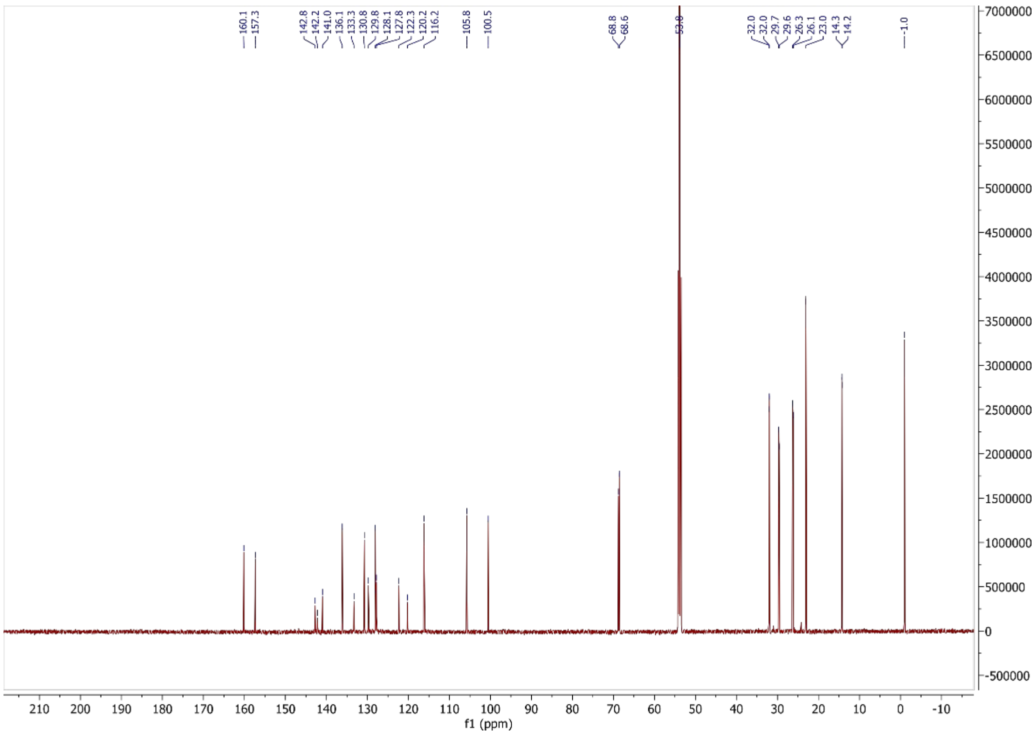

Figure S20 ^{13}C NMR (150 MHz, CD_2Cl_2) of compound **9**.

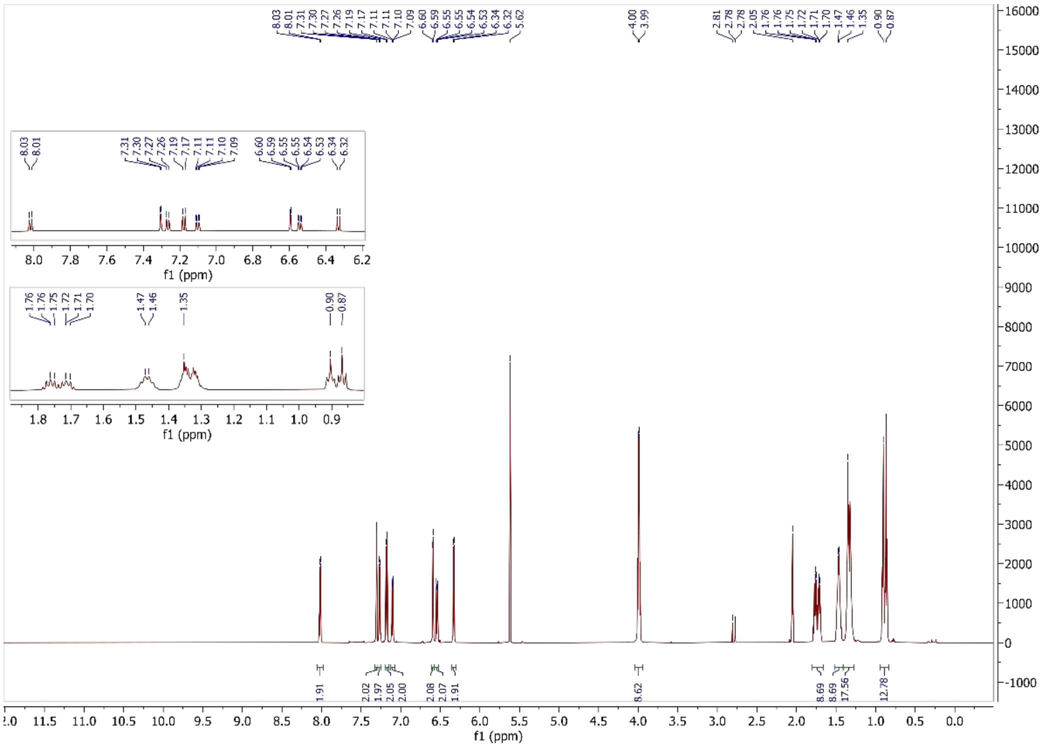

Figure S21 ^{1}H NMR (600 MHz, acetone-d_6) of compound **10**.

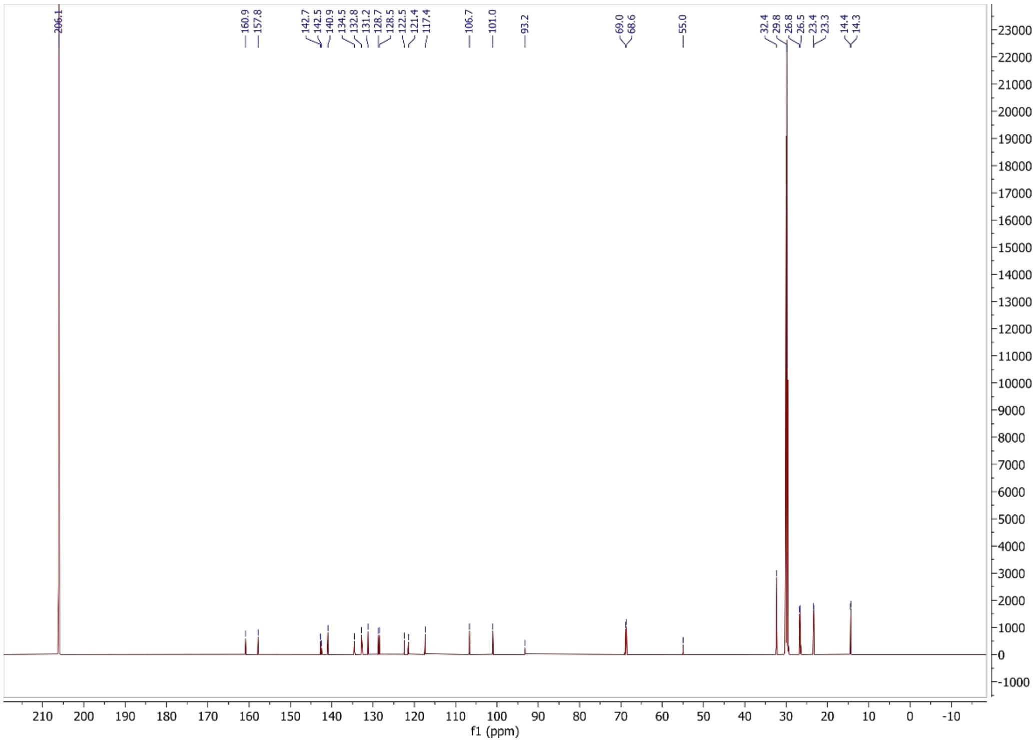

Figure S22 ^{13}C NMR (150 MHz, acetone-d_6) of compound **10**.

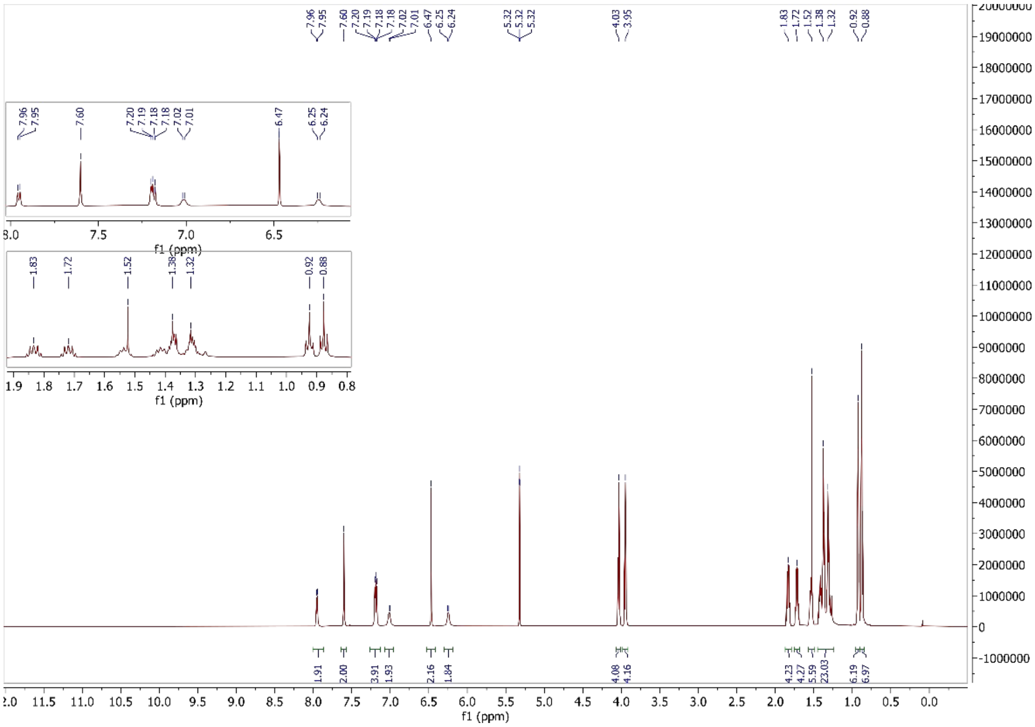

Figure S23 ^{1}H NMR (600 MHz, CD_2Cl_2) of compound **10-by**.

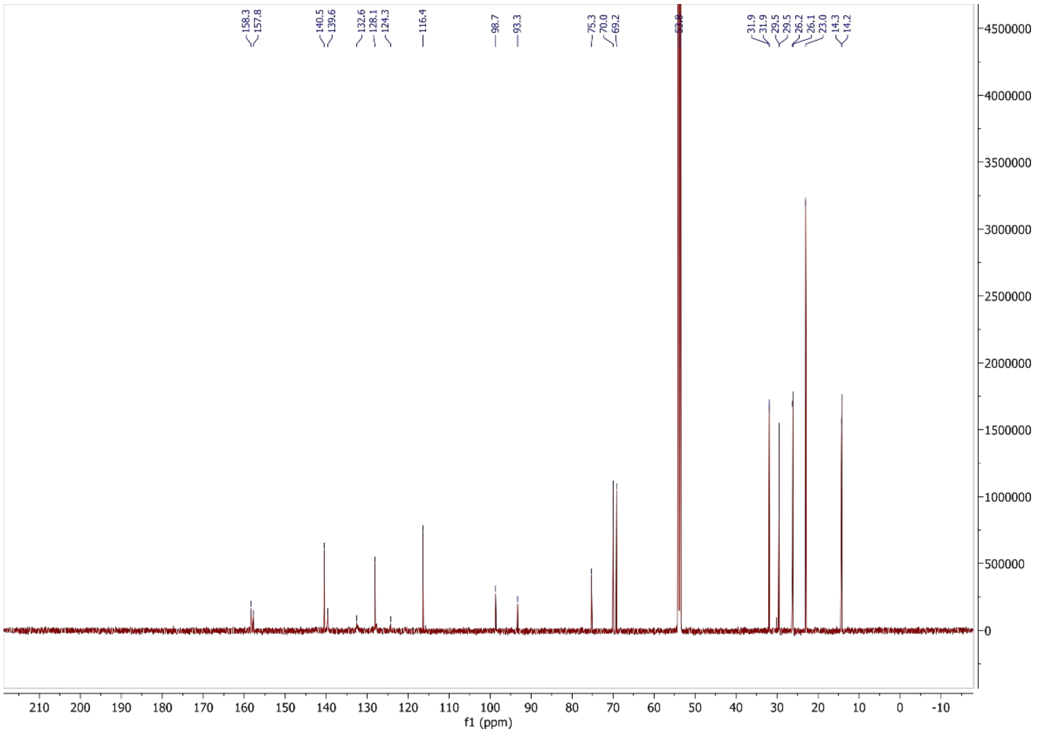

Figure S24 ^{13}C NMR (150 MHz, CD_2Cl_2) of compound **10-by**.

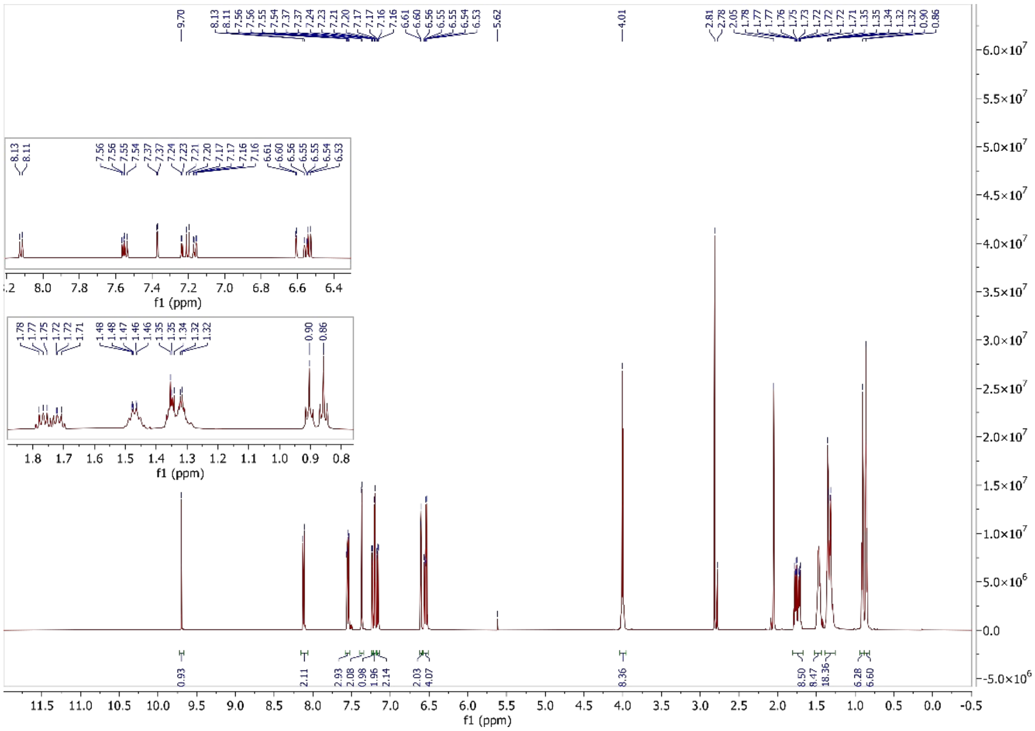

Figure S25 ^{1}H NMR (600 MHz, acetone-d_6) of compound **11**.

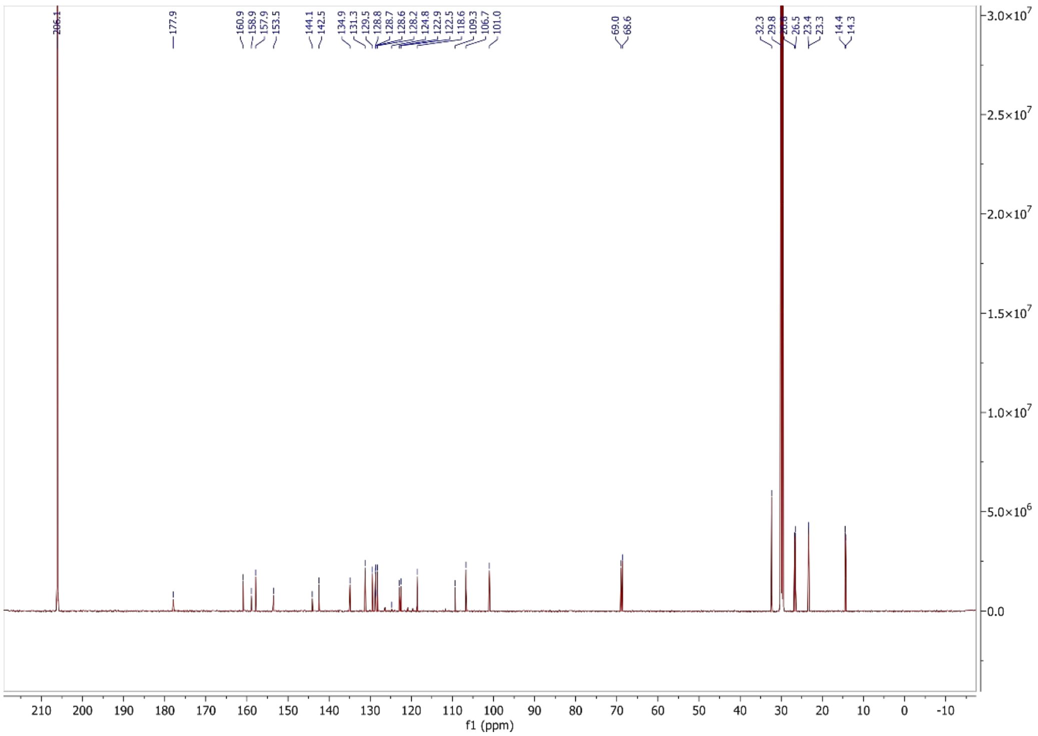

Figure S26 ^{13}C NMR (150 MHz, acetone-d_6) of compound **11**.

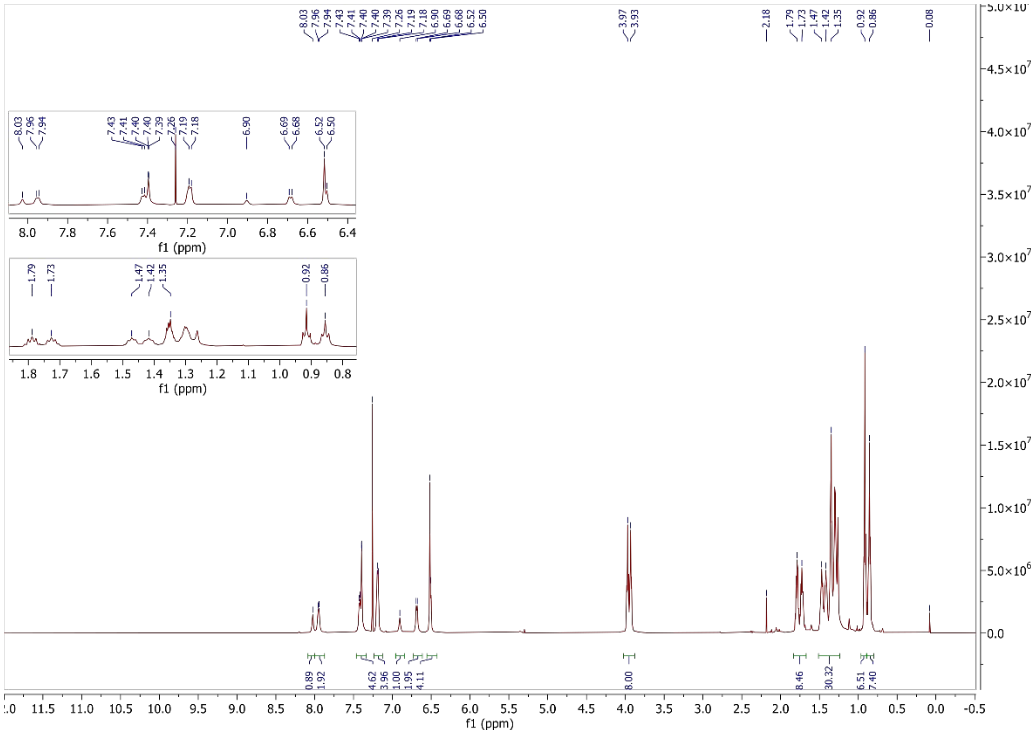

Figure S27 ^{1}H NMR (600 MHz, $CDCl_3$) of dye **10*H*-PTZ-Fu**.

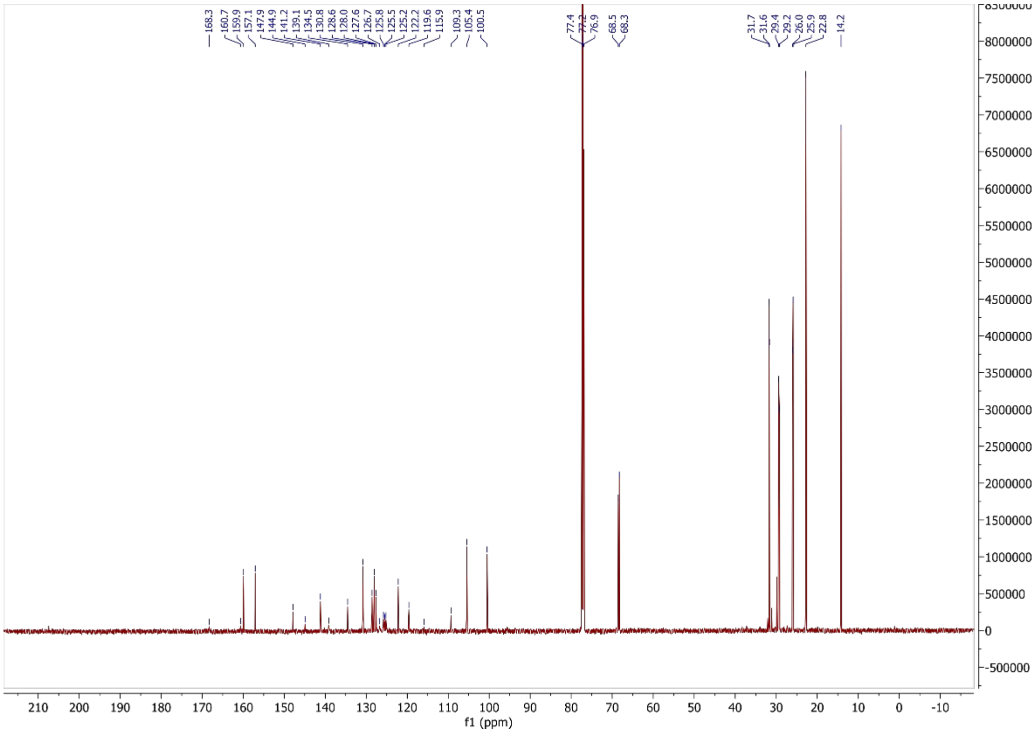

Figure S28 ^{13}C NMR (150 MHz, $CDCl_3$) of dye **10*H*-PTZ-Fu**.

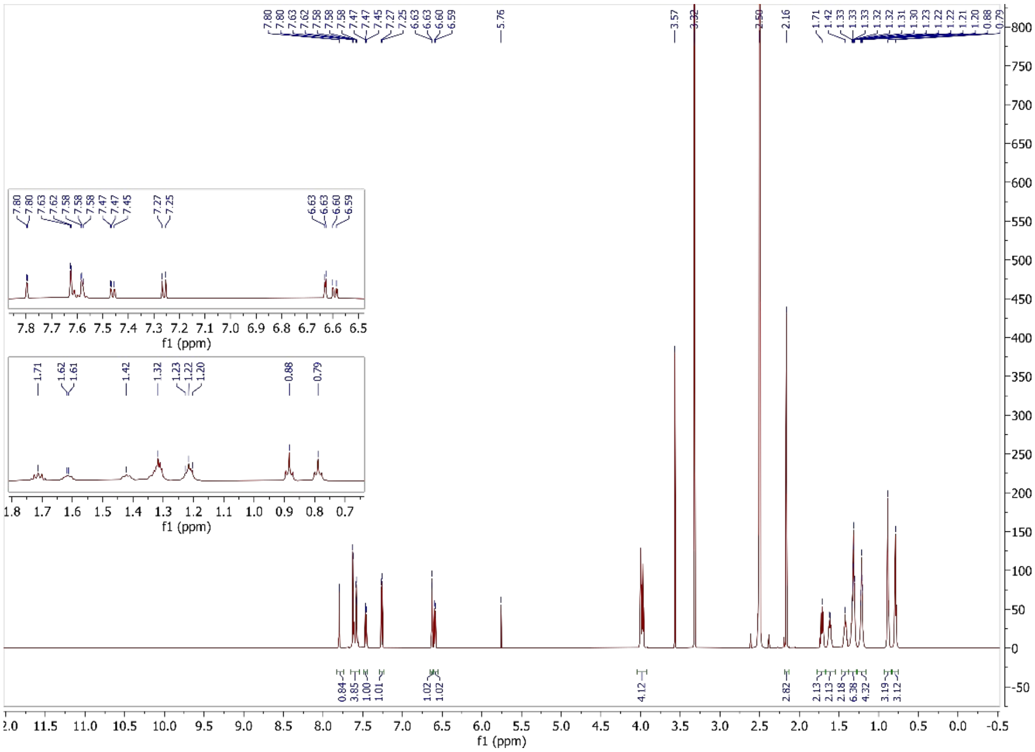

Figure S29 ^{1}H NMR (600 MHz, DMSO-d_6) of compound **12**.

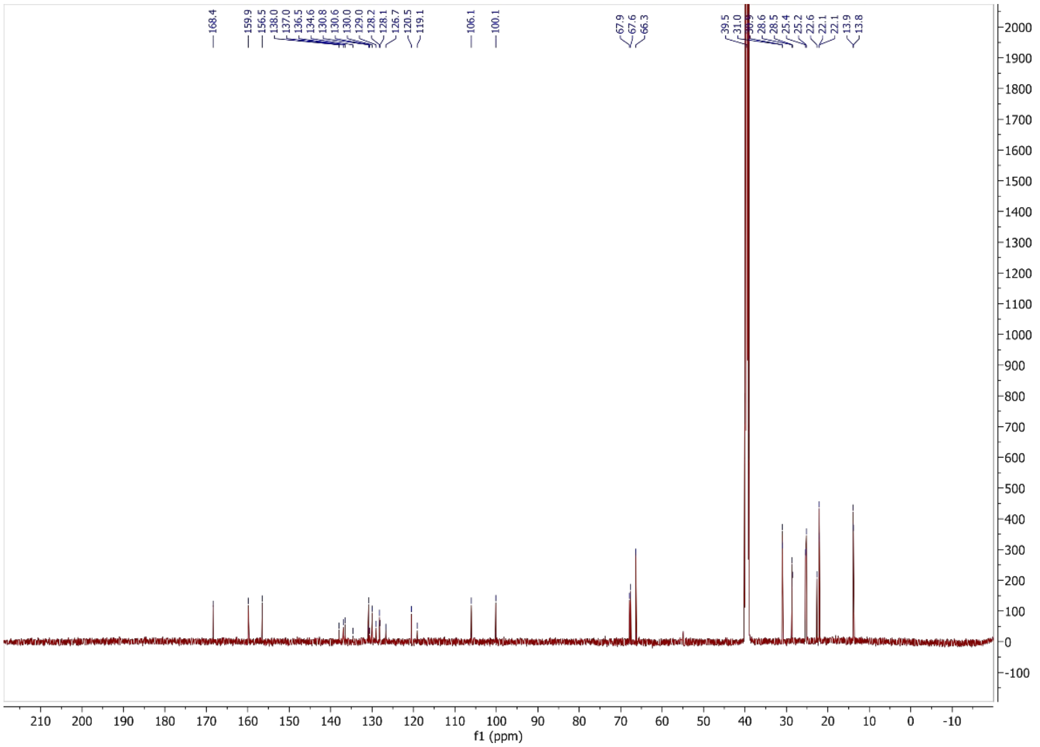

Figure S30 ^{13}C NMR (150 MHz, DMSO-d_6) of compound **12**.

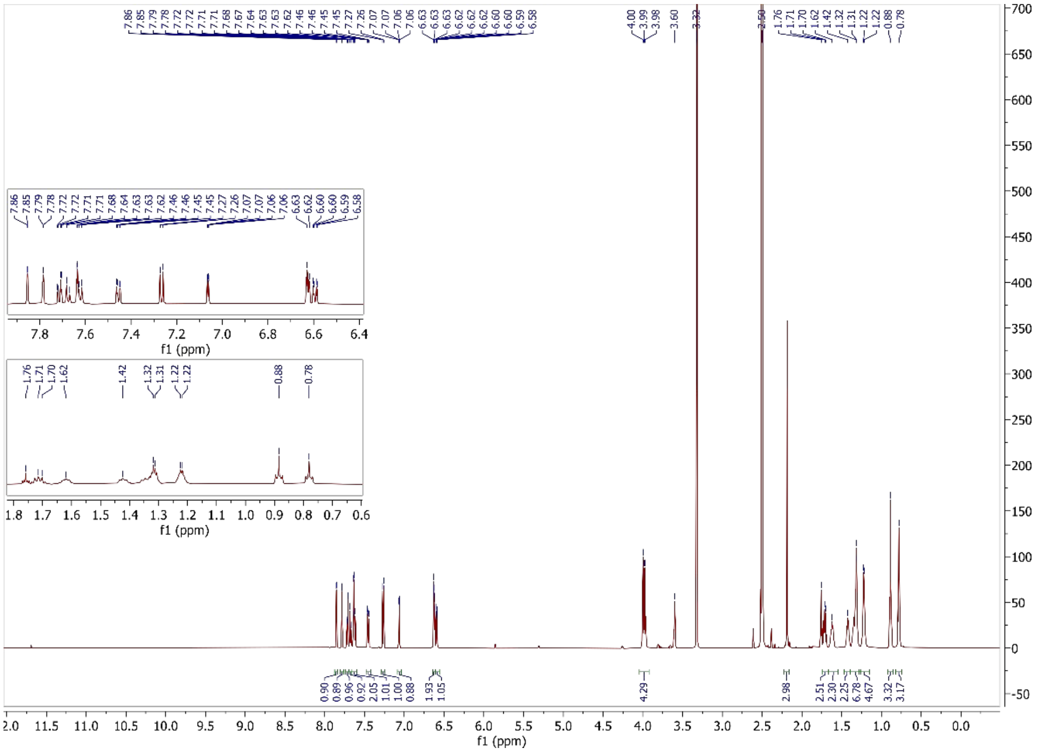

Figure S31 ^{1}H NMR (600 MHz, DMSO-d_6) of compound **13**.

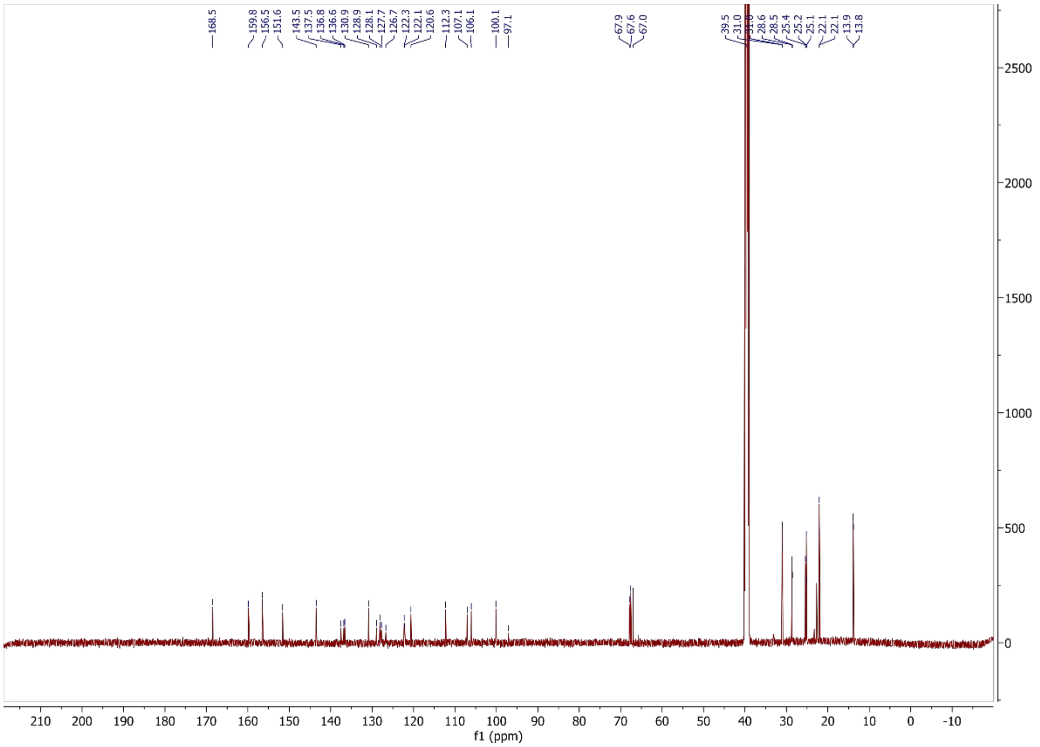

Figure S32 ^{13}C NMR (150 MHz, DMSO-d_6) of compound **13**.

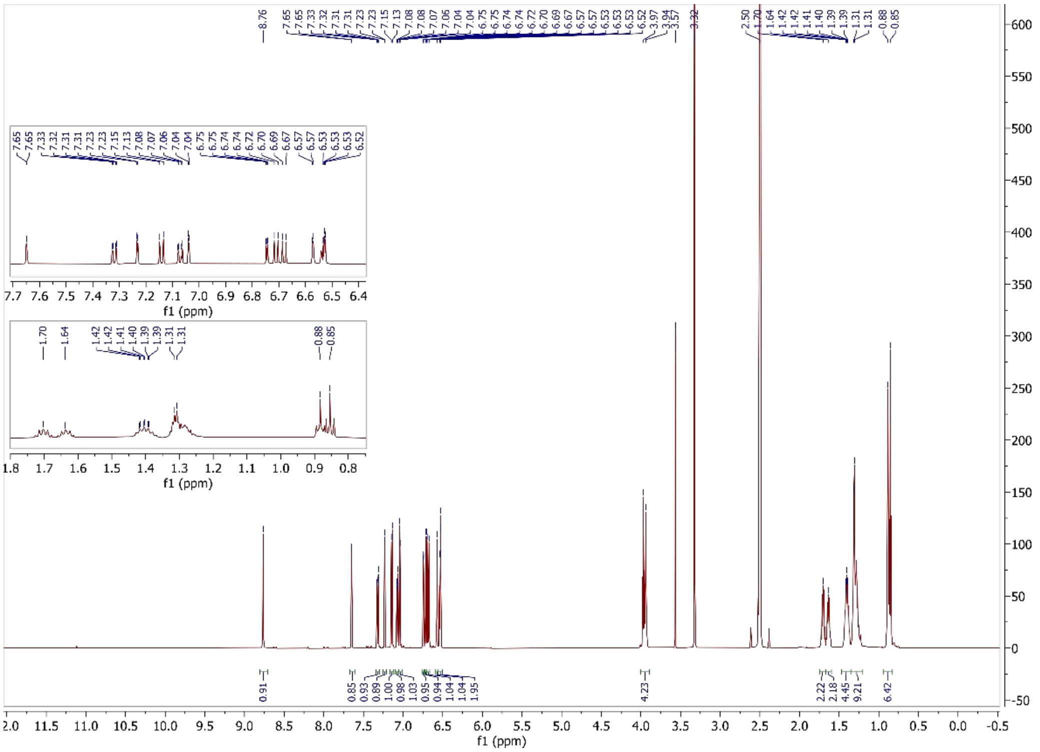

Figure S33 ^{1}H NMR (600 MHz, DMSO-d_6) of compound **14**.

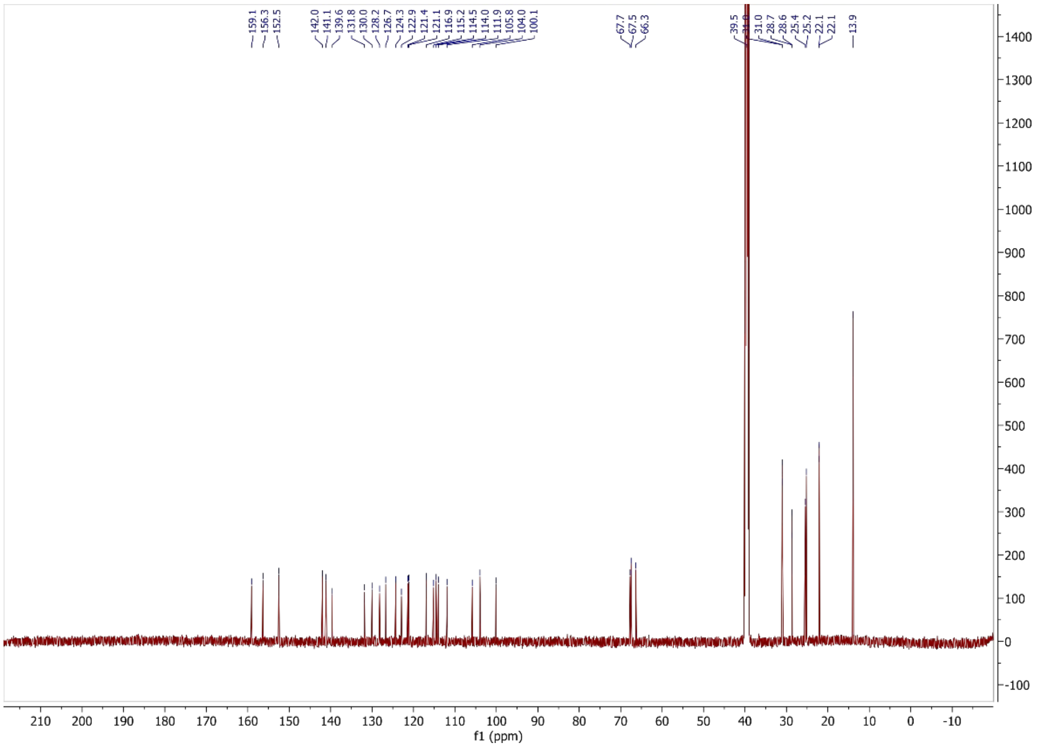

Figure S34 ^{13}C NMR (150 MHz, DMSO-d_6) of compound **14**.

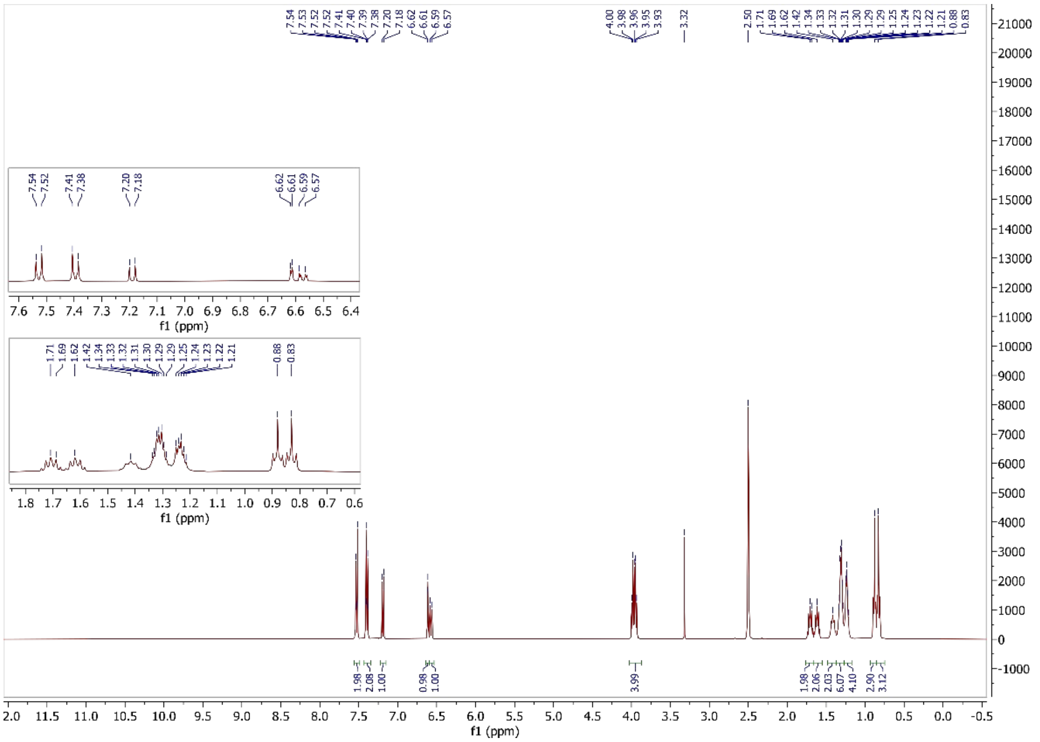

Figure S35 ^{1}H NMR (600 MHz, DMSO-d_6) of compound **16**.

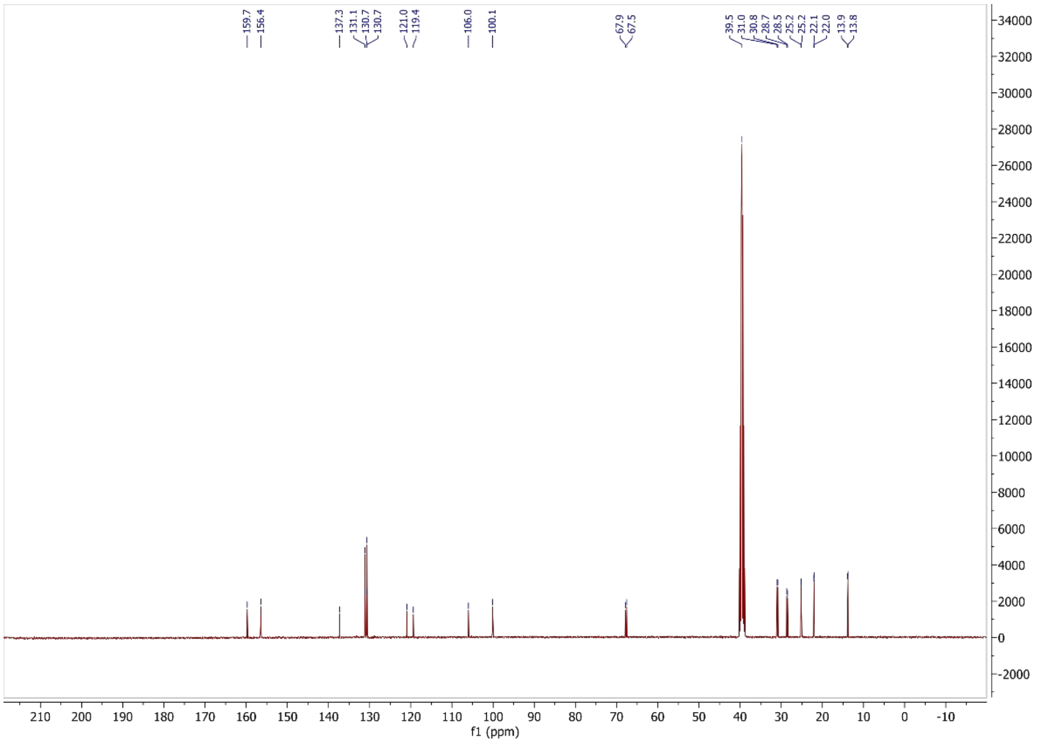

Figure S36 ^{13}C NMR (150 MHz, DMSO-d_6) of compound **16**.

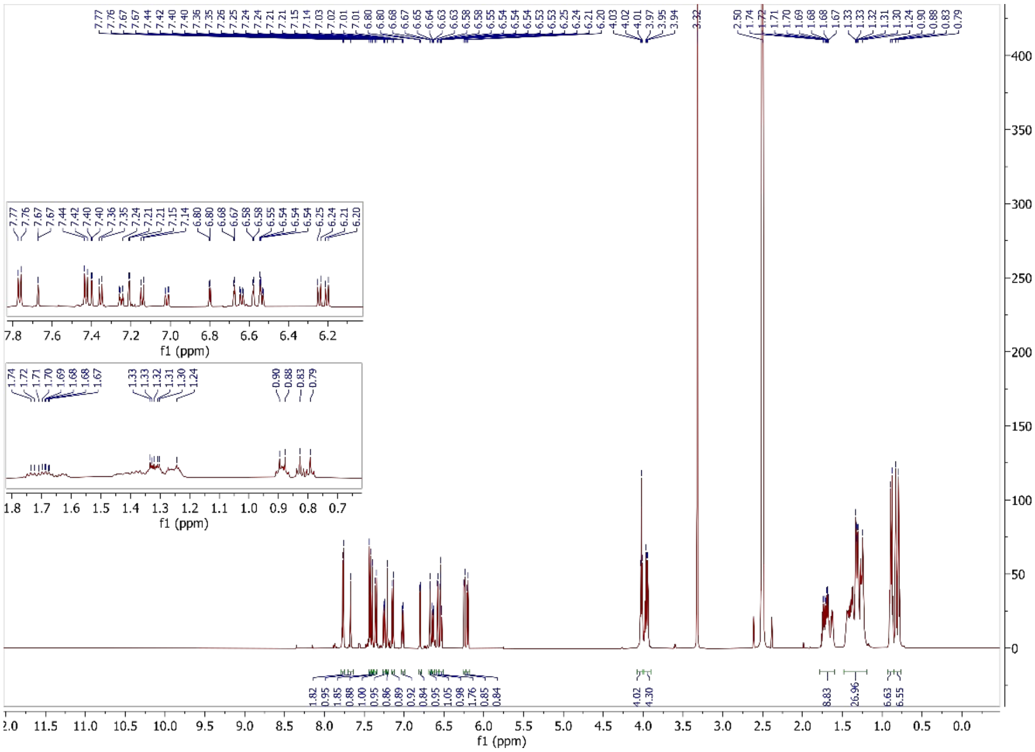

Figure S37 ^{1}H NMR (600 MHz, DMSO-d_6) of compound **17**.

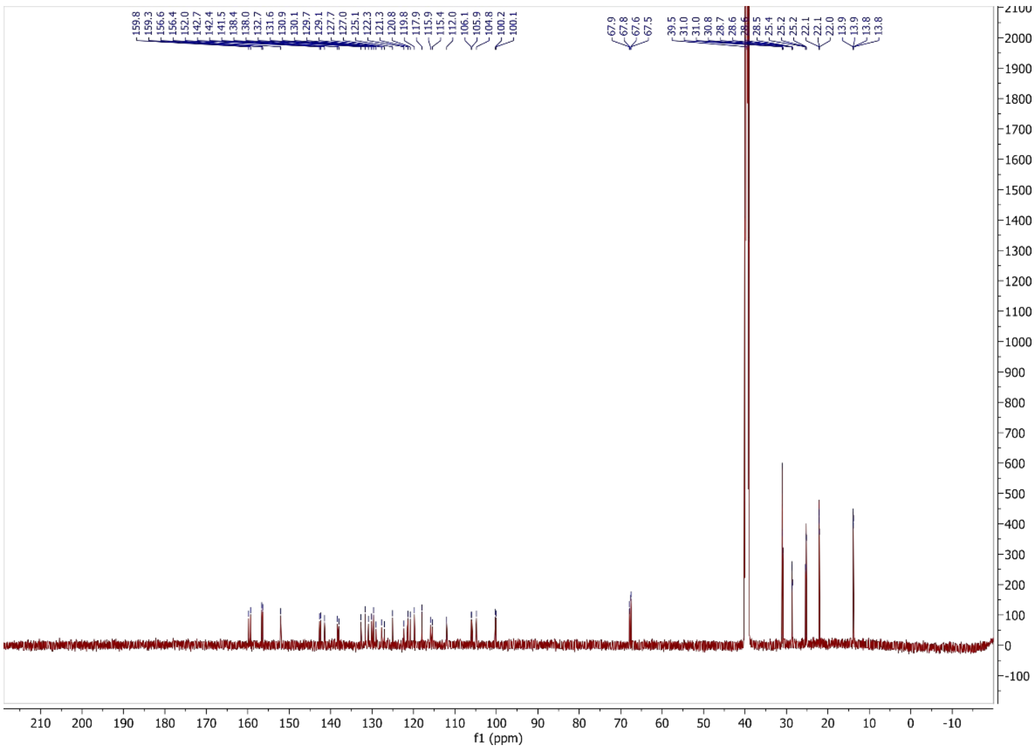

Figure S38 ^{13}C NMR (150 MHz, DMSO-d_6) of compound **17**.

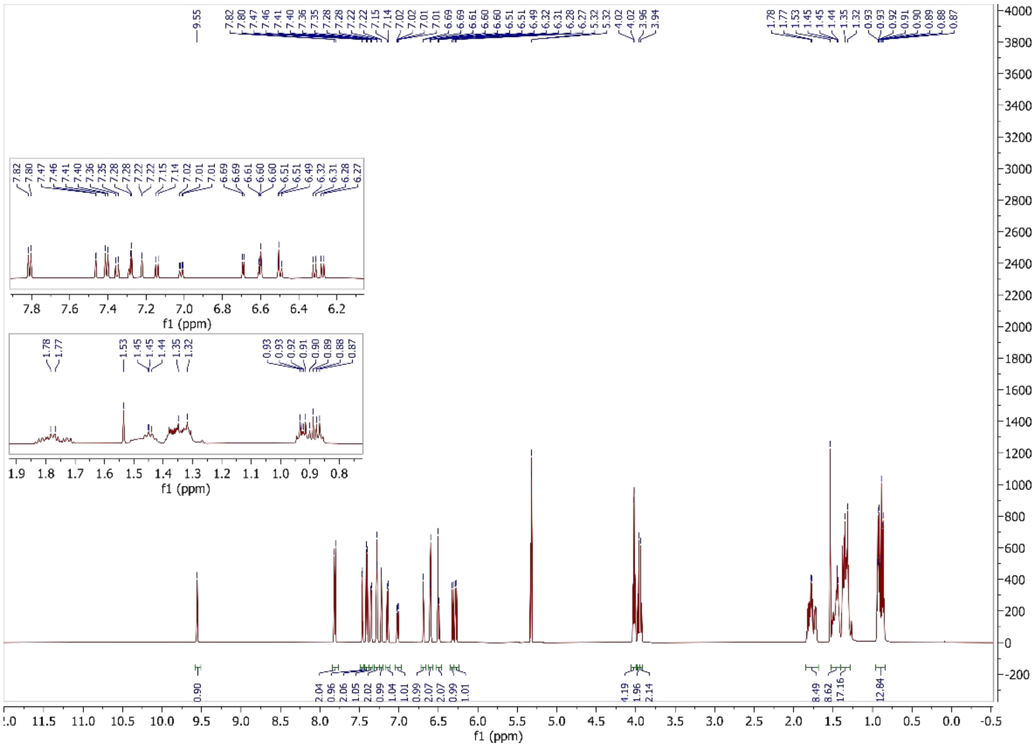

Figure S39 ^{1}H NMR (600 MHz, CD_2Cl_2) of compound **18**.

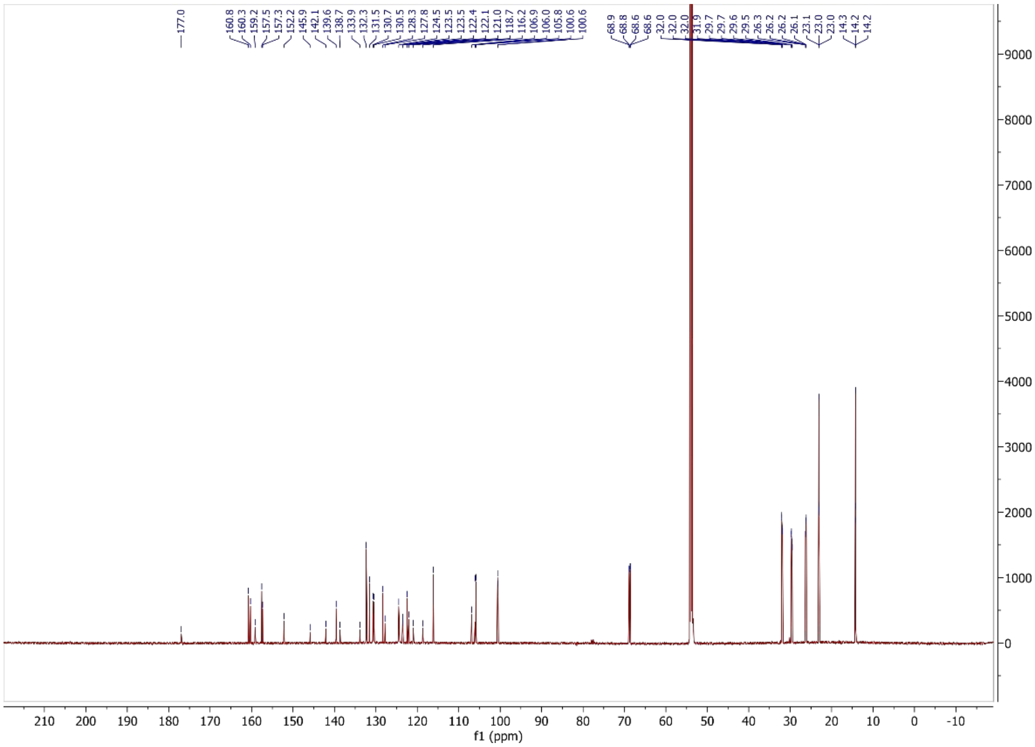

Figure S40 ^{13}C NMR (150 MHz, CD_2Cl_2) of compound **18**.

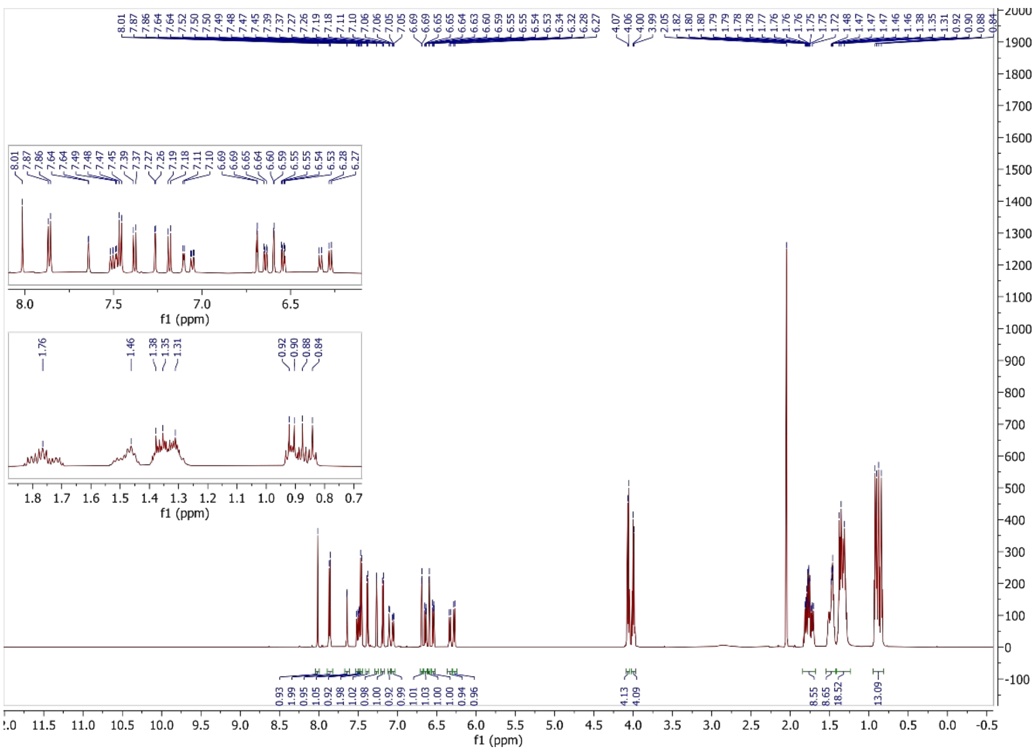

Figure S41 ^{1}H NMR (600 MHz, acetone-d_6) of dye **3,7-PTZ-Fu**.

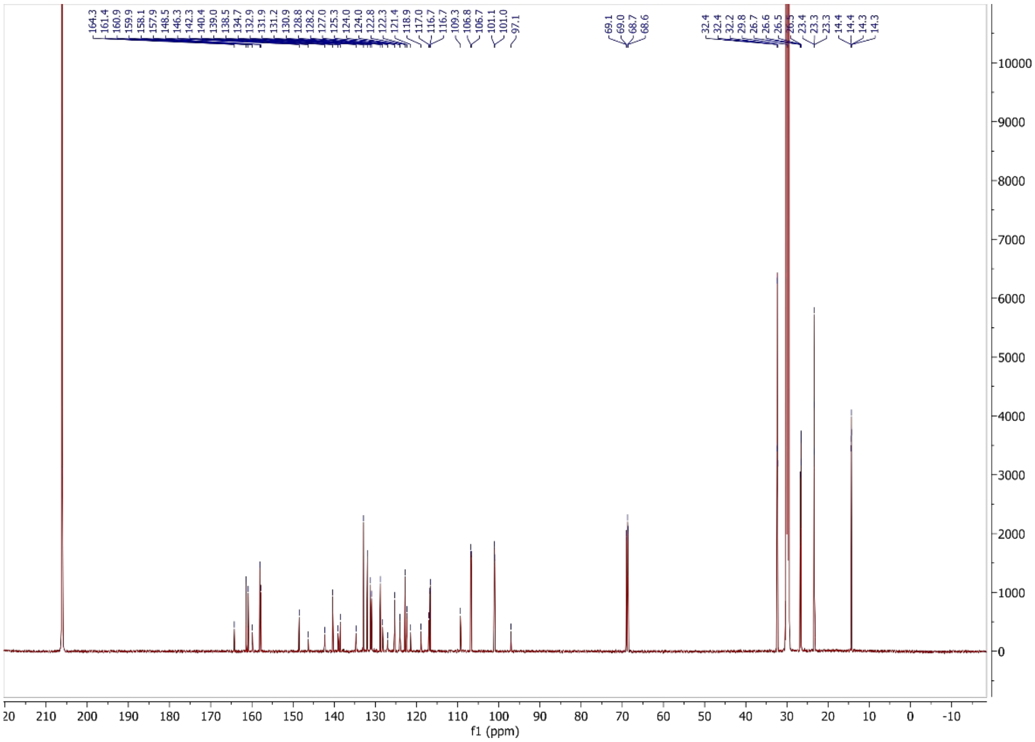

Figure S42 ^{13}C NMR (150 MHz, acetone-d_6) of dye **3,7-PTZ-Fu**.

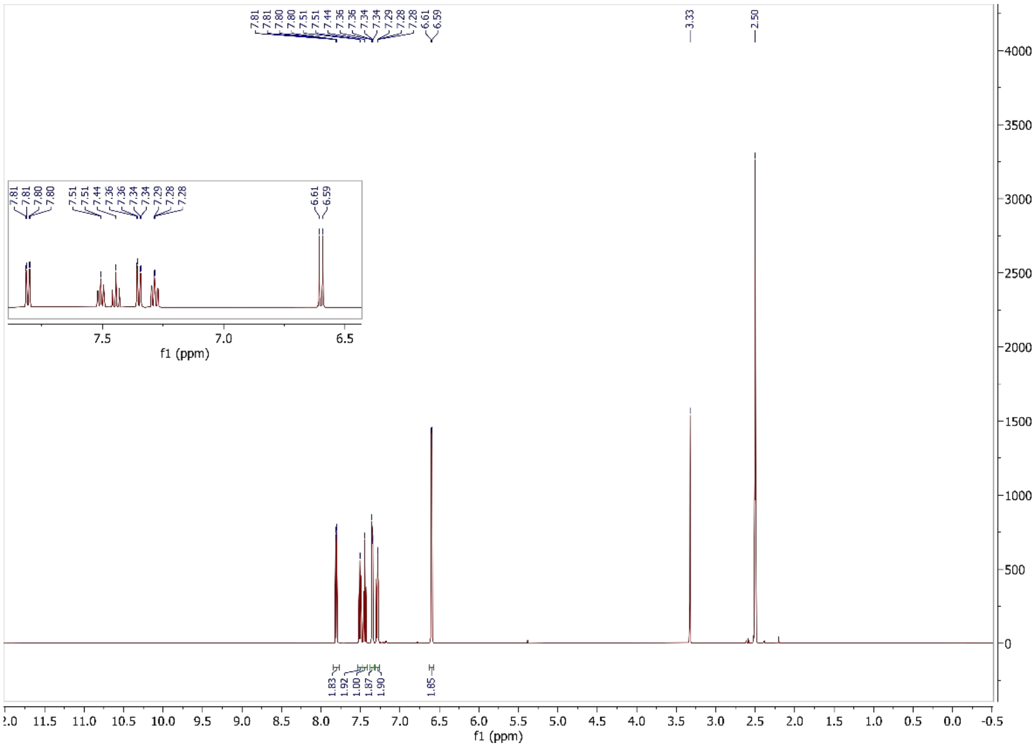

Figure S43 ^{1}H NMR (600 MHz, DMSO-d_6) of compound **21**.

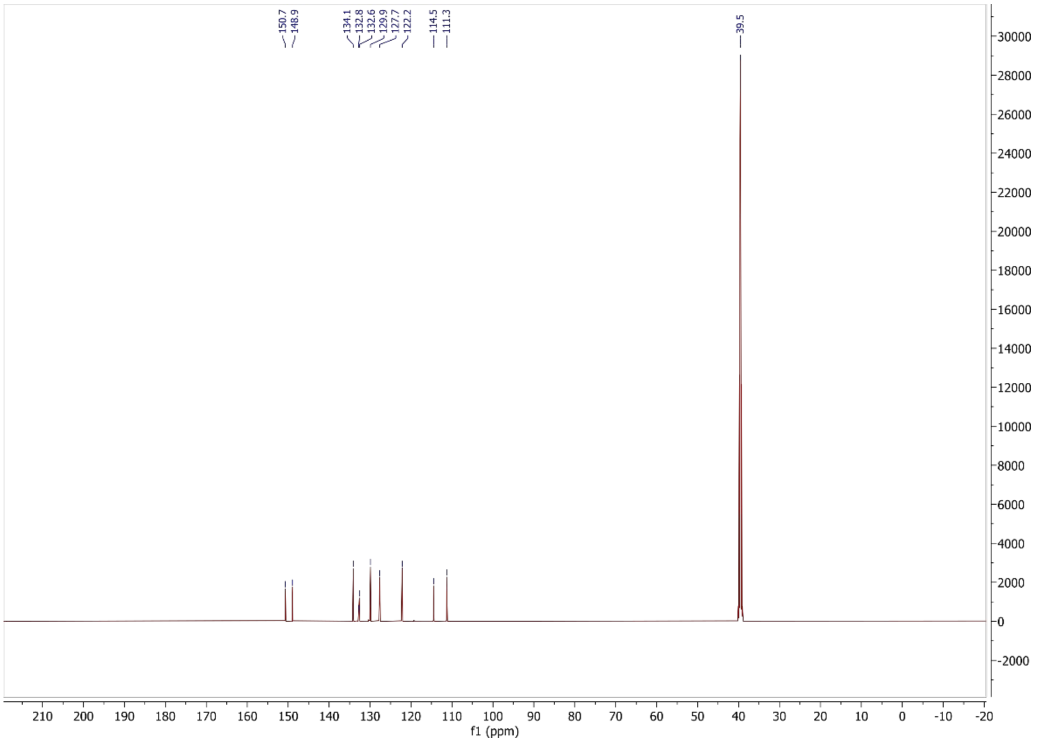

Figure S44 ^{13}C NMR (150 MHz, DMSO-d_6) of compound **21**.

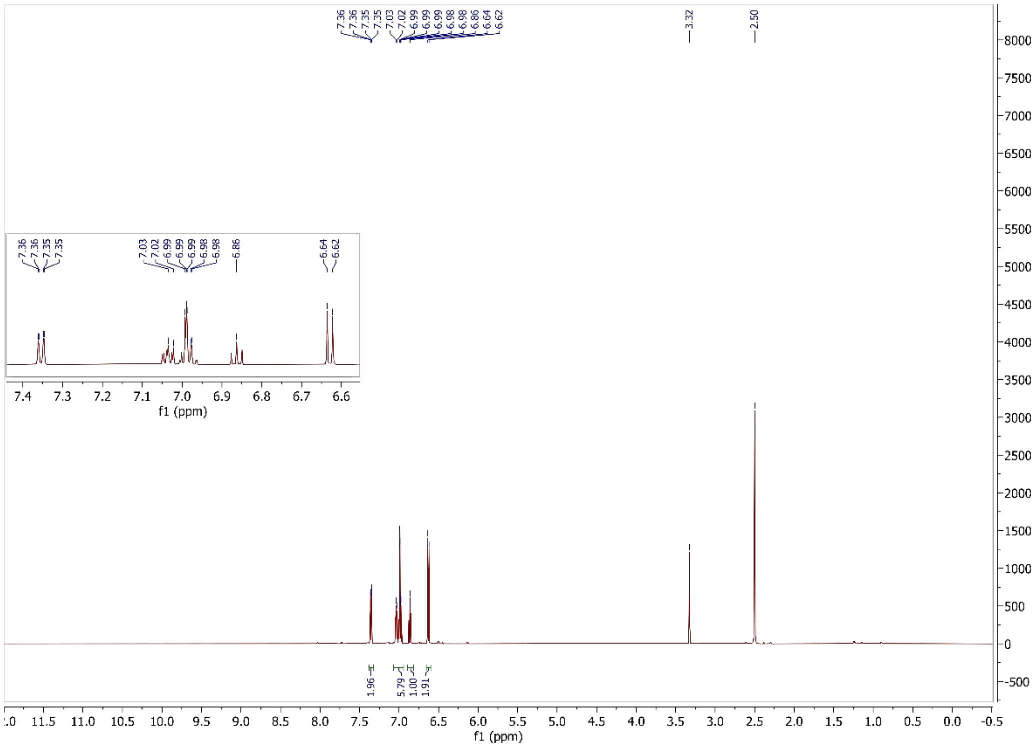

Figure S45 ^{1}H NMR (600 MHz, DMSO-d_6) of compound **23**.

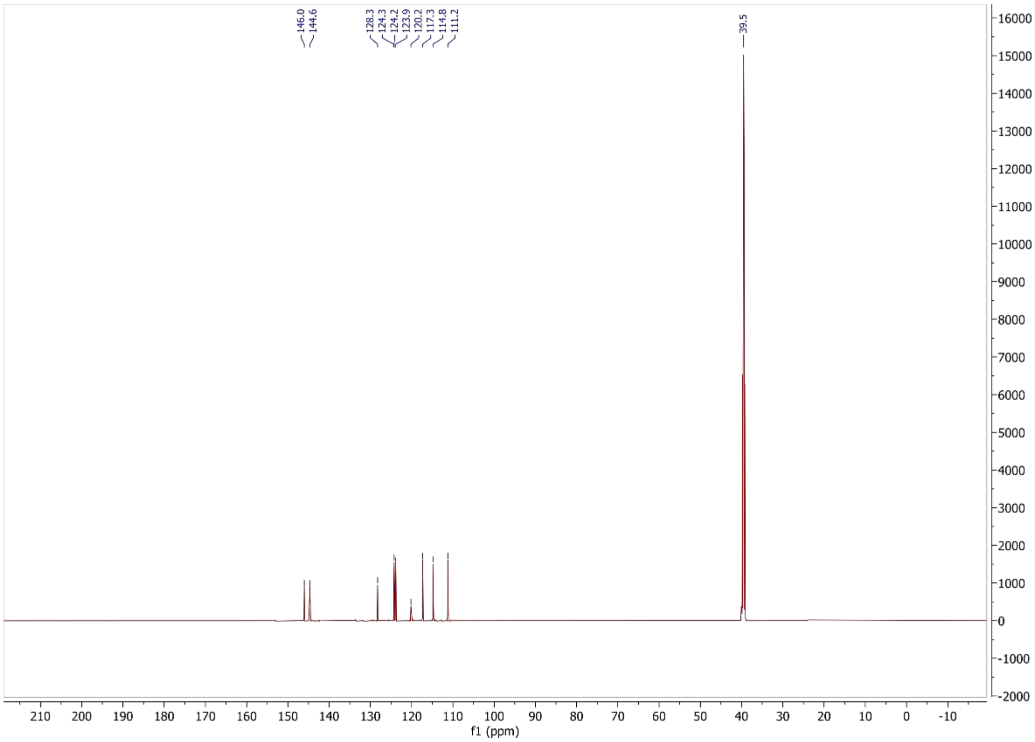

Figure S46 ^{13}C NMR (150 MHz, DMSO-d_6) of compound **23**.

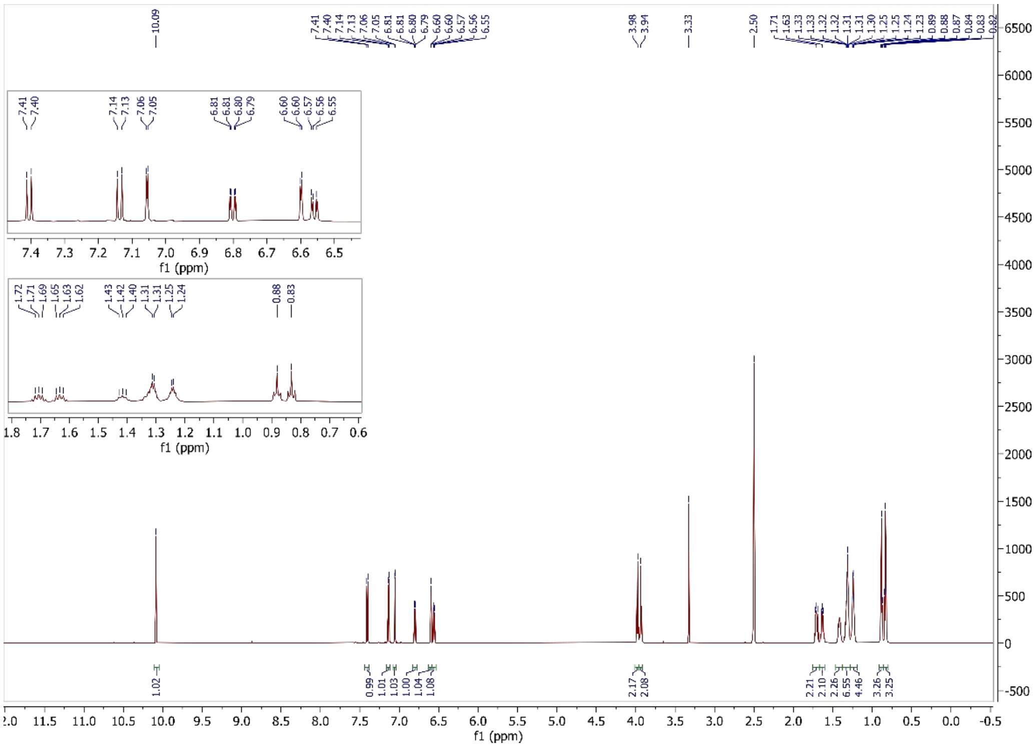

Figure S47 ^{1}H NMR (600 MHz, DMSO-d_6) of compound **26**.

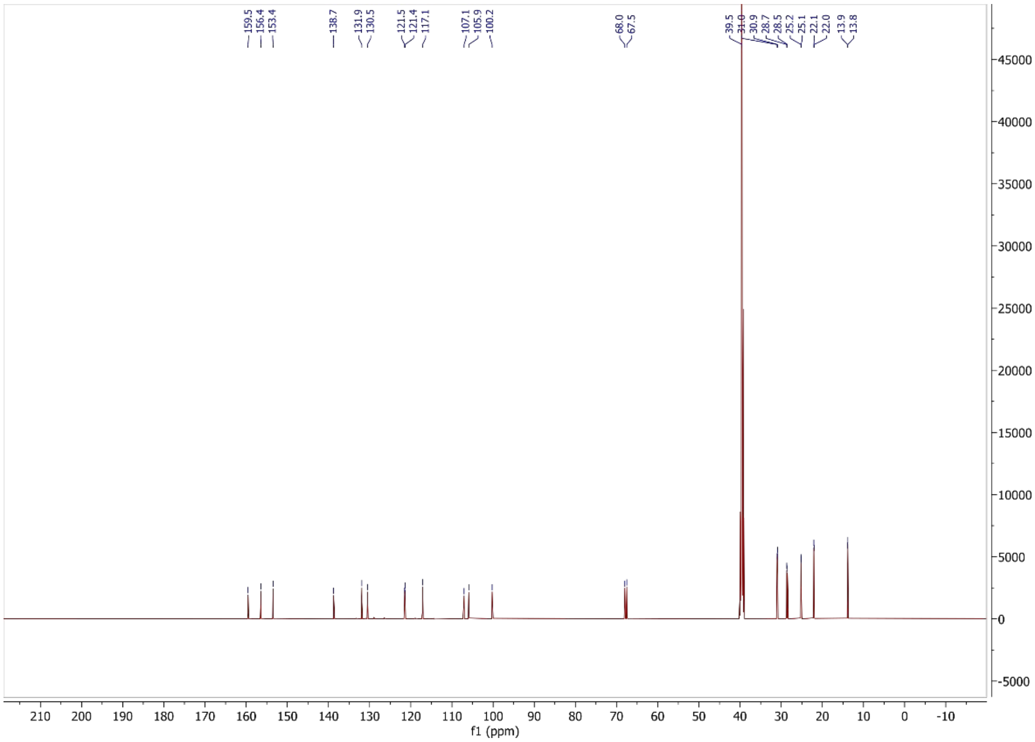

Figure S48 ^{13}C NMR (150 MHz, DMSO-d_6) of compound **26**.

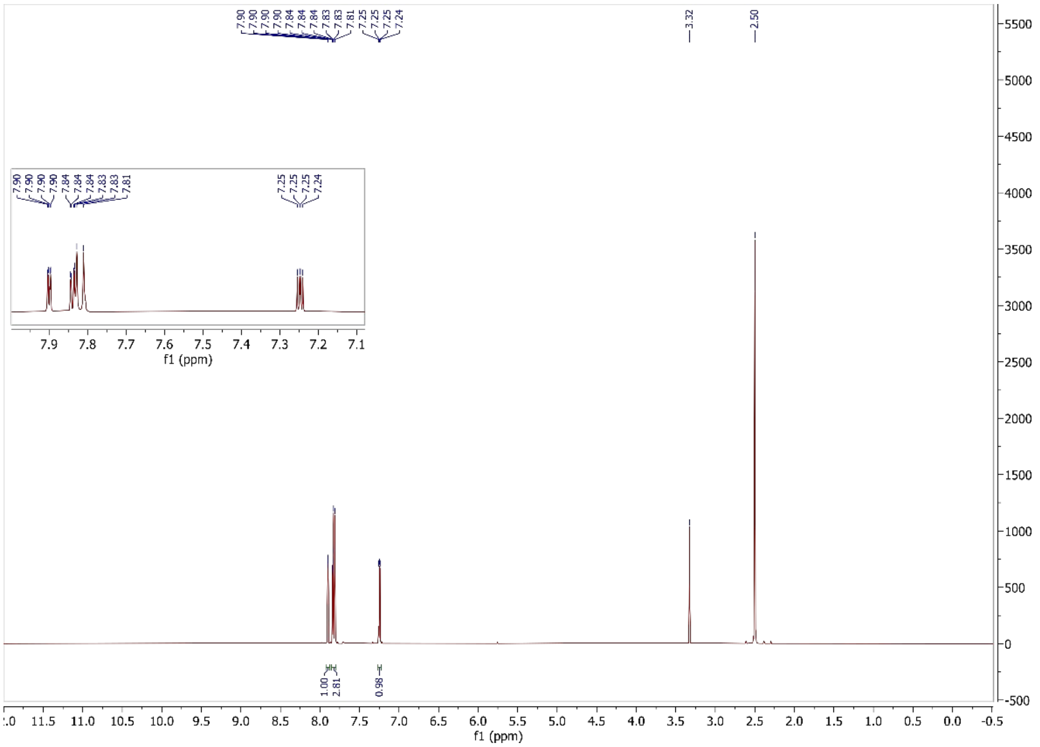

Figure S49 ^{1}H NMR (600 MHz, DMSO-d_6) of compound **28**.

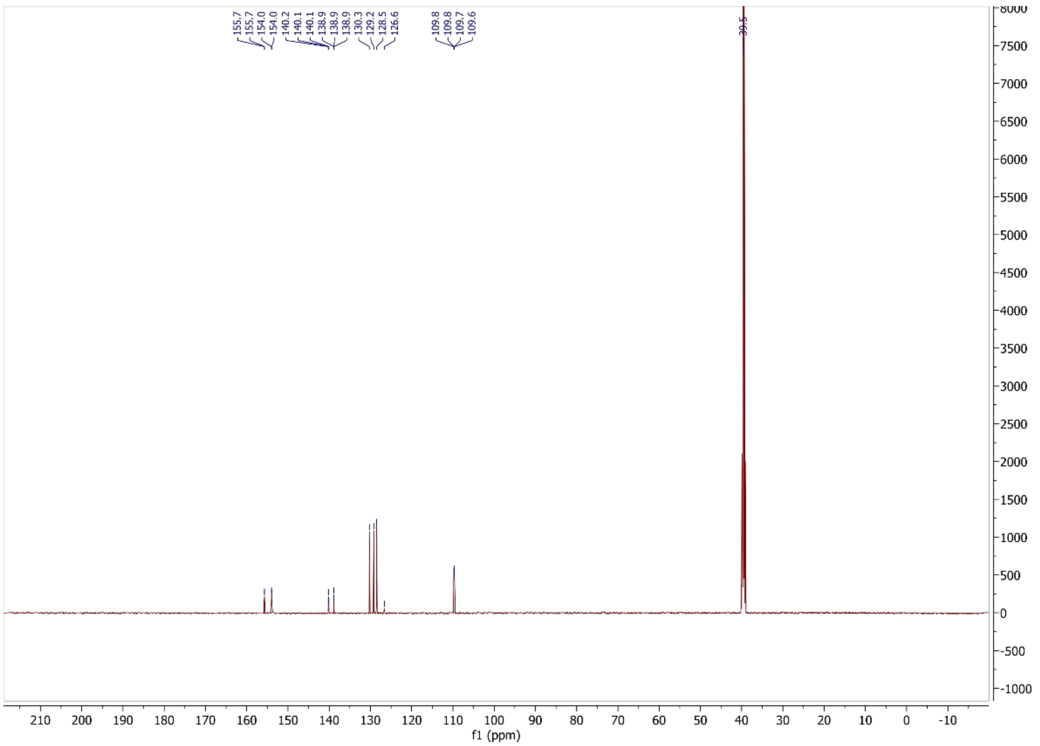

Figure S50 ^{13}C NMR (150 MHz, DMSO-d_6) of compound **28**.

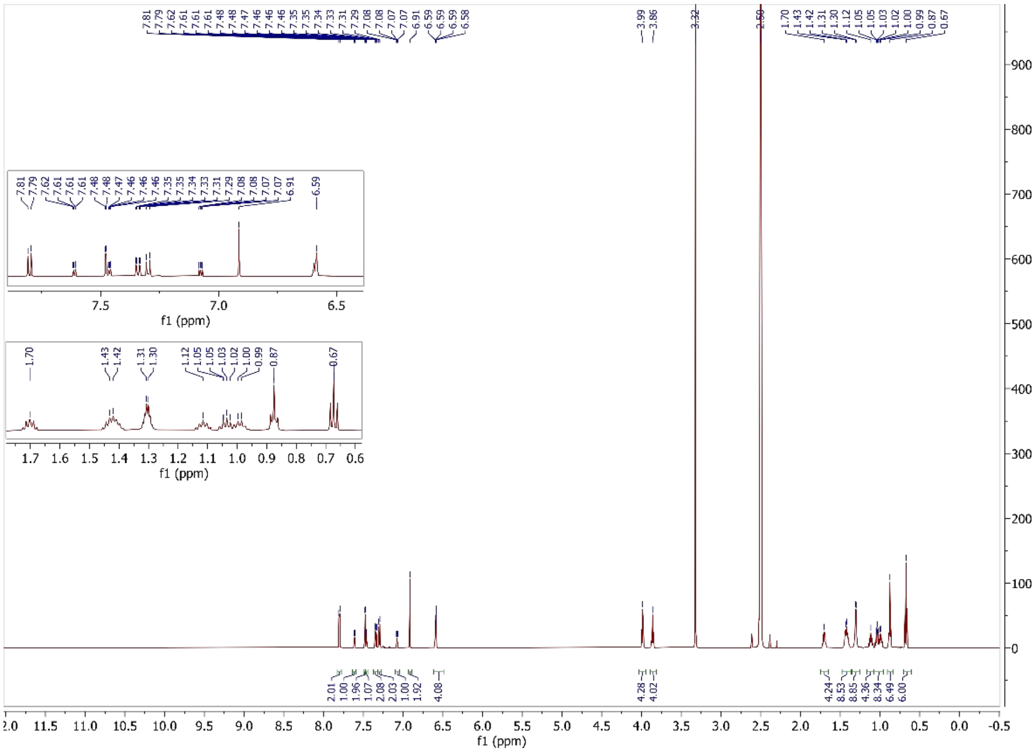

Figure S51 ^{1}H NMR (600 MHz, DMSO-d_6) of compound **29**.

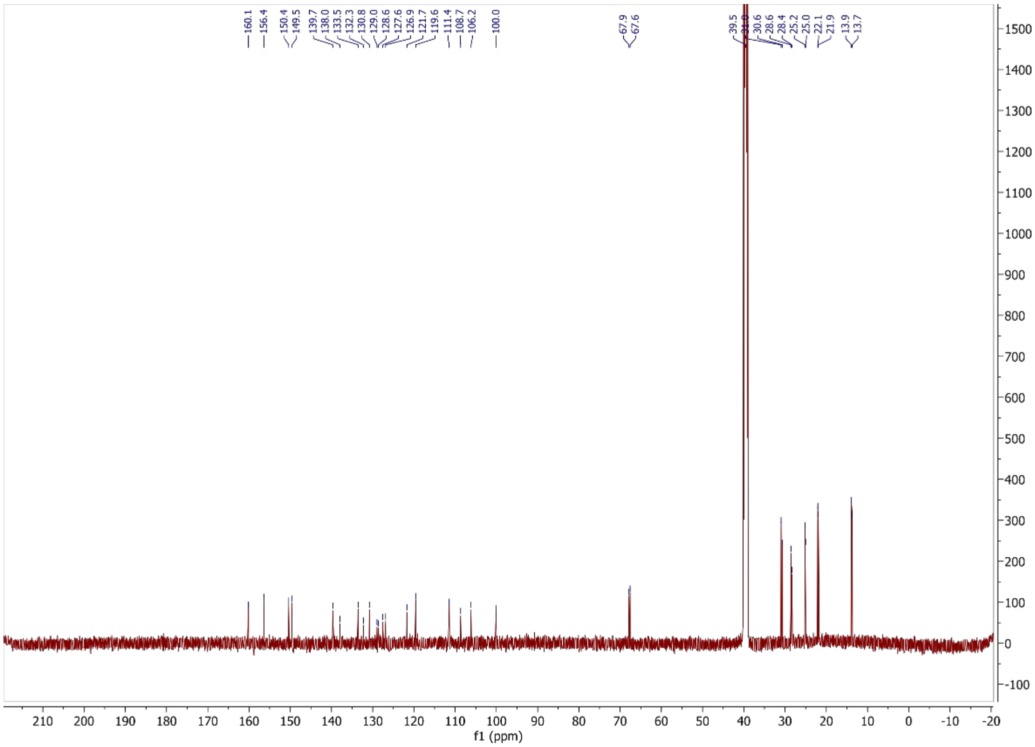

Figure S52 ^{13}C NMR (150 MHz, DMSO-d_6) of compound **29**.

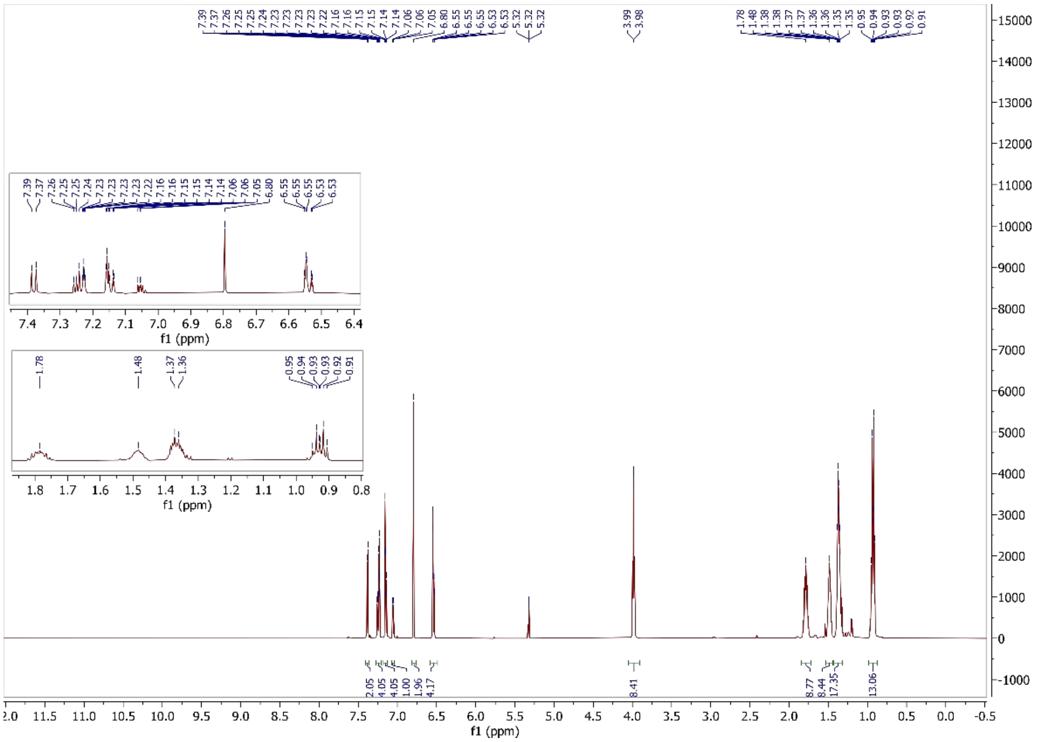

Figure S53 ^{1}H NMR (600 MHz, CD_2Cl_2) of compound **31**.

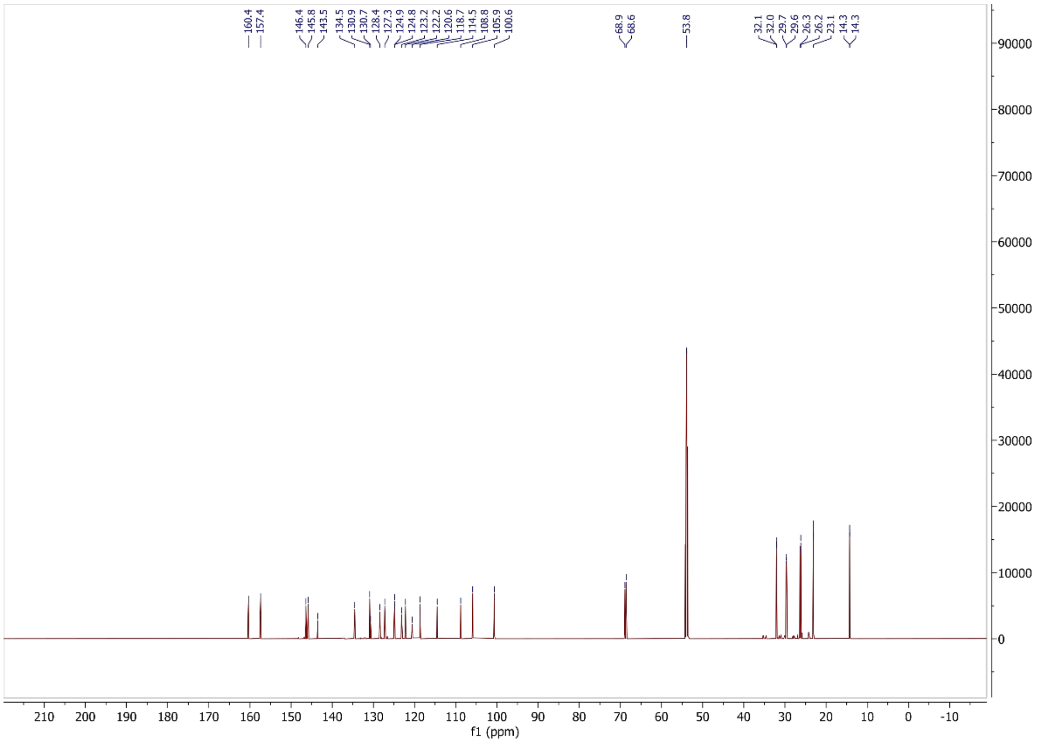

Figure S54 ^{13}C NMR (150 MHz, CD_2Cl_2) of compound **31**.

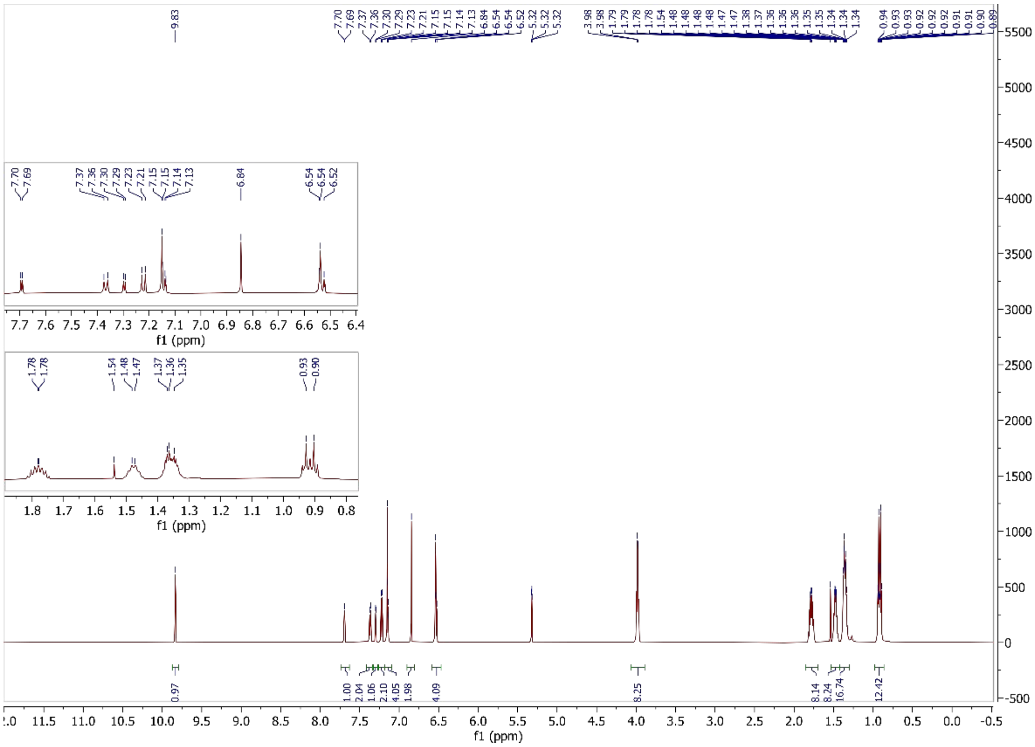

Figure S55 ^{1}H NMR (600 MHz, CD_2Cl_2) of compound **32**.

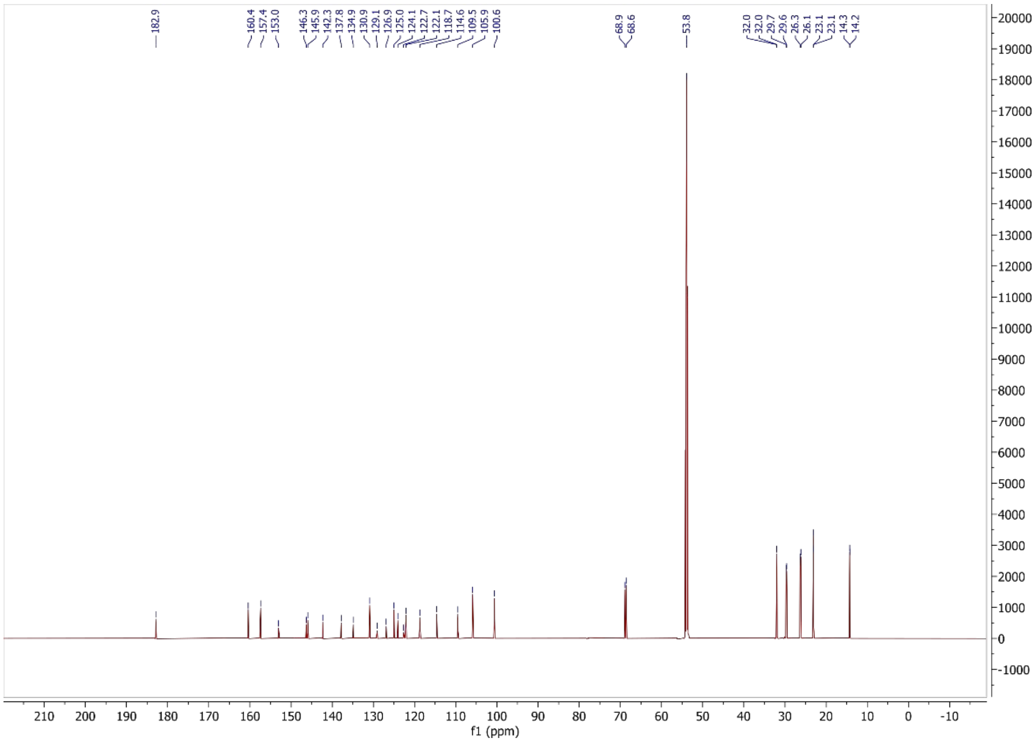

Figure S56 ^{13}C NMR (150 MHz, CD_2Cl_2) of compound **32**.

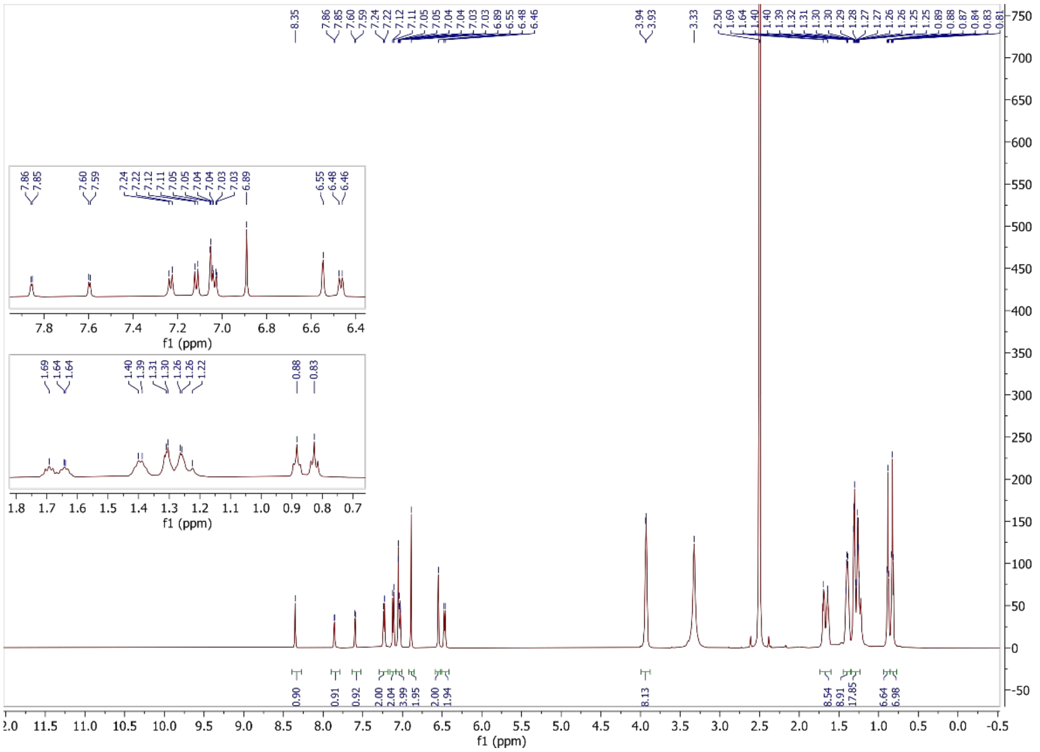

Figure S57 ^{1}H NMR (600 MHz, DMSO-d_6) of compound **POZPOZ-Th**.

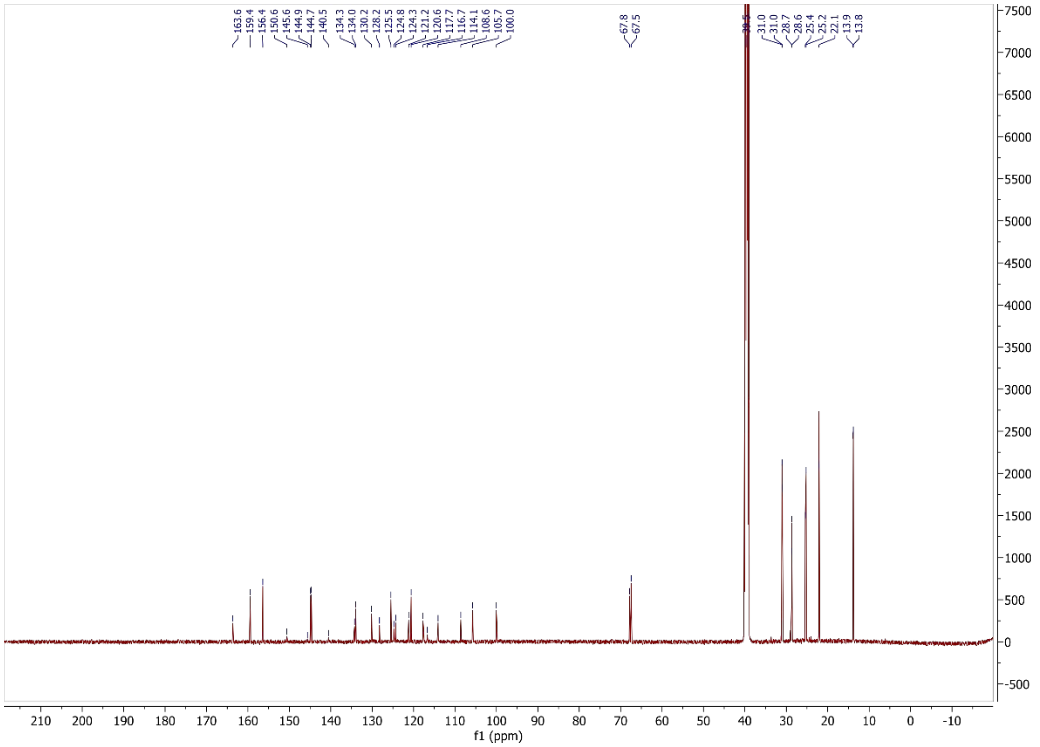

Figure S58 ^{13}C NMR (150 MHz, DMSO-d_6) of compound **POZPOZ-Th**.
